# Representation of people with comorbidity and multimorbidity in clinical trials of novel drug therapies: an individual-level participant data analysis

**DOI:** 10.1186/s12916-019-1427-1

**Published:** 2019-11-12

**Authors:** Peter Hanlon, Laurie Hannigan, Jesus Rodriguez-Perez, Colin Fischbacher, Nicky J. Welton, Sofia Dias, Frances S. Mair, Bruce Guthrie, Sarah Wild, David A. McAllister

**Affiliations:** 1 Institute for Health and Wellbeing, University of Glasgow, 1 Lilybank Gardens, Glasgow, G12 8RZ UK; 2 Information Services Division, NHS Scotland, Edinburgh, UK; 3 Population Health Sciences, Bristol Medical School, University of Bristol, Bristol, UK; 4 Usher Institute for Population Health Sciences, University of Edinburgh, Edinburgh, UK

**Keywords:** Randomised controlled trials, Comorbidity, Multimorbidity

## Abstract

**Background:**

Clinicians are less likely to prescribe guideline-recommended treatments to people with multimorbidity than to people with a single condition. Doubts as to the applicability of clinical trials of drug treatments (the gold standard for evidence-based medicine) when people have co-existing diseases (comorbidity) may underlie this apparent reluctance. Therefore, for a range of index conditions, we measured the comorbidity among participants in clinical trials of novel drug therapies and compared this to the comorbidity among patients in the community.

**Methods:**

Data from industry-sponsored phase 3/4 multicentre trials of novel drug therapies for chronic medical conditions were identified from two repositories: Clinical Study Data Request and the Yale University Open Data Access project. We identified 116 trials (*n* = 122,969 participants) for 22 index conditions. Community patients were identified from a nationally representative sample of 2.3 million patients in Wales, UK. Twenty-one comorbidities were identified from medication use based on pre-specified definitions. We assessed the prevalence of each comorbidity and the total number of comorbidities (level of multimorbidity), for each trial and in community patients.

**Results:**

In the trials, the commonest comorbidities in order of declining prevalence were chronic pain, cardiovascular disease, arthritis, affective disorders, acid-related disorders, asthma/COPD and diabetes. These conditions were also common in community-based patients.

Mean comorbidity count for trial participants was approximately half that seen in community-based patients. Nonetheless, a substantial proportion of trial participants had a high degree of multimorbidity. For example, in asthma and psoriasis trials, 10–15% of participants had ≥ 3 conditions overall, while in osteoporosis and chronic obstructive pulmonary disease trials 40–60% of participants had ≥ 3 conditions overall.

**Conclusions:**

Comorbidity and multimorbidity are less common in trials than in community populations with the same index condition. Comorbidity and multimorbidity are, nevertheless, common in trials. This suggests that standard, industry-funded clinical trials are an underused resource for investigating treatment effects in people with comorbidity and multimorbidity.

## Background

Drug treatments that have been recommended in evidence-based clinical guidelines are less likely to be prescribed to people with multimorbidity (defined as people with two or more conditions) [1–5]. One reason for this difference in prescribing is that the populations included in clinical trials, which underpin evidence-based guidelines, are believed to be unrepresentative of people with multimorbidity [6, 7].

Comorbidity (the presence of other conditions in addition to a specified index condition) [8] may influence the effectiveness of treatments for specific conditions through competing risks, drug-drug, drug-disease and disease-disease interactions, altering the balance of risks and benefits [9–11]. Underrepresentation of people with multimorbidity in clinical trials is therefore concerning.

However, most studies examining clinical trial representativeness have done so by analysing routine clinical practice data (e.g. from disease registers and electronic health records) to which trial eligibility criteria have been applied [12–17]. Since factors other than eligibility criteria are likely to influence which people are recruited to clinical trials [18], such approaches provide only indirect evidence about the prevalence of comorbidity and multimorbidity in trial participants.

We examined the prevalence of comorbidity and multimorbidity among 122,969 participants from 116 industry-funded trials of novel drug therapies for 22 index conditions and compared these results with comorbidity and multimorbidity prevalence in 2.3 million patients living in the community.

## Methods

### Study design

This cross-sectional analysis compares the distribution of comorbidity and multimorbidity in participants enrolled in 116 industry-sponsored trials and a representative community sample from the UK. All analyses were pre-specified (Additional file 1).

### Data sources and participants

#### Trials

**Fig. 1:**
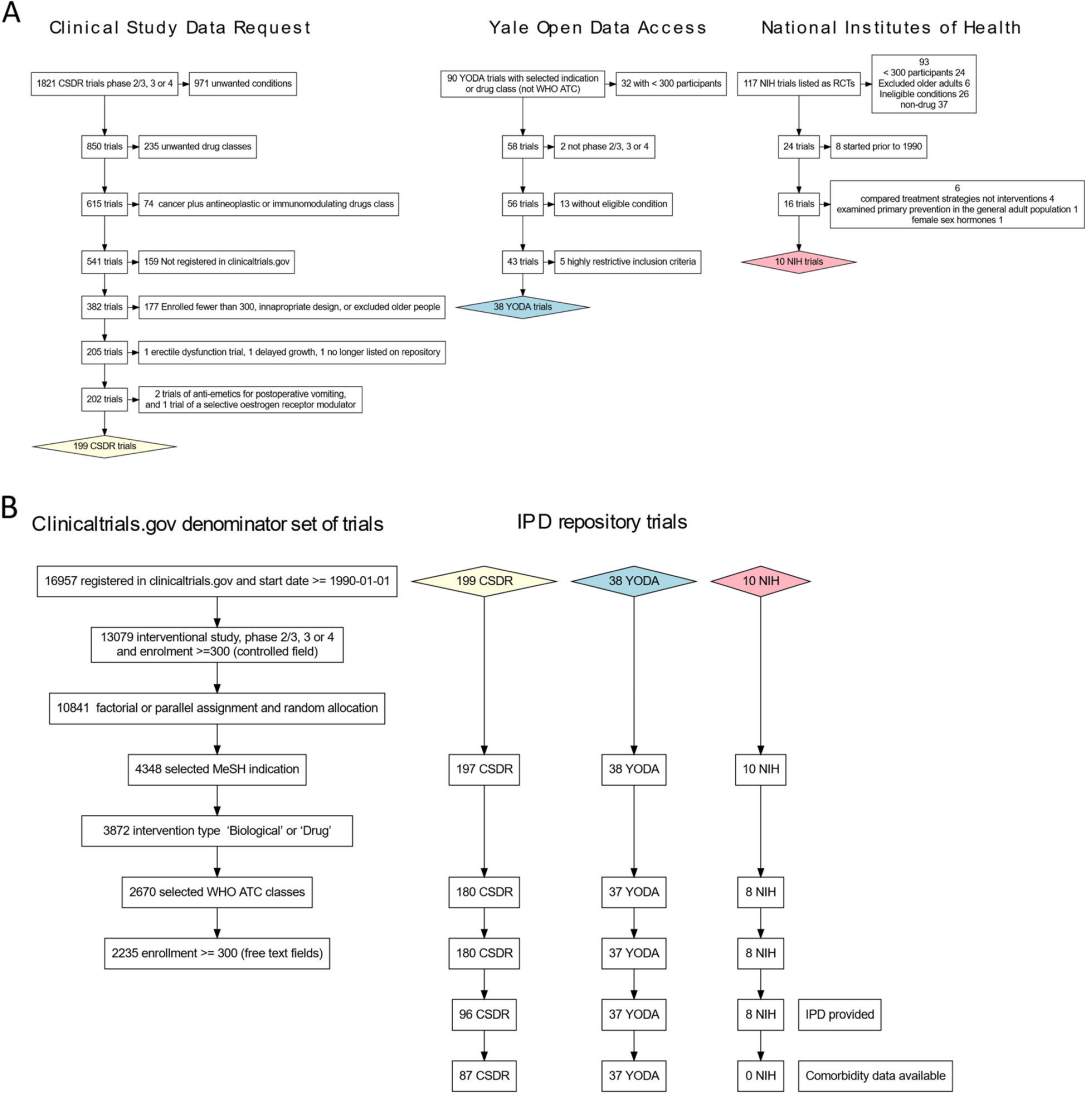
**a** Initial identification of individual-level participant data trials from trial repositories. See Additional file 1 for a detailed description of the selection process. Abbreviations are as follows: MeSH, Medical Subject Headings; WHO-ATC, World Health Organization Anatomic Therapeutic Chemical classification scheme; CSDR, Clinical Study Data Request repository; YODA, Yale Open Data Access repository; NIH, National Institutes of Health Biologic Specimen and Data Repository Information Coordinating Center repository; and IPD, individual-level participant data. **b** Definition of “denominator trials” using the US clinical trials registry (clinicaltrials.gov) and the effect of restricting the individual-level participant data trials to this denominator set. The height of each box on the horizontal axis corresponds to the simultaneous effect of applying the eligibility criteria to the denominator set of trials (the leftmost chart) and the three numerator sets of IPD trials. For brevity, the leftmost flowchart shows both the eligibility criteria and the trial counts whereas the other three flowcharts show only the trial counts. Of the final set of 124 trials, further 8 trials were excluded either because the index condition was either difficult or impossible to accurately identify within the primary care record or because we judged that concomitant medication may be difficult to interpret in the context of the index condition (see Additional file 1, section 1.7 for details)

We accessed individual-level participant data (IPD) from industry-sponsored trials from two repositories: the Clinical Study Data Request (CSDR) and the Yale University Open Data Access (YODA) project (on 21 November 2016 and 18 May 2018, respectively). From this set, trials were selected according to a pre-specified protocol (Prospero CRD42018048202) [19]. Briefly, eligible trials were registered with the US Clinical Trials register (clincialtrials.gov), had a start date on or after 1 January 1990 (based on scoping showing that trials where IPD was available had started on or after this date), were phase 2/3, 3 or 4, recruited ≥ 300 participants, had an upper age limit ≥ 60 years (or no maximum) and evaluated drugs for a selected set of chronic conditions (Fig. 1). Conditions were chosen on the basis that they require long-term pharmacological therapy. We selected a range of cardiovascular, respiratory, gastrointestinal, musculoskeletal, metabolic, autoimmune and connective tissue, and urological and otolaryngological disorders. A full list of eligible conditions is shown in Additional file 1: Table S1.8. Trials for neoplastic, infectious, affective, psychotic or developmental disorders were excluded, as were trials of primary prevention in general populations without an index condition (see Additional file 1). Only randomised participants were included in analyses. We also searched the National Institutes of Health (NIH) Biologic Specimen and Data Repository Information Coordinating Center (BioLINCC) repository in August 2017, but no trials from this source were eligible because of lack of reported data on comorbidities.

#### Community sample

A community sample was identified using the Secure Anonymised Information Linkage (SAIL) Databank, which is a repository of health and administrative data covering 70% of Wales’s population of three million [20]. This sample is nationally representative in terms of age, sex and socioeconomic status (Additional file 2). We included people registered with a participating primary care practice between 1 January 2011 and 1 January 2012 (2,289,583 people). This time period was chosen after accessing the primary care data, prior to further analysis, as coding of prescribing data was most complete from this point onwards.

### Index conditions

For trial data, index conditions were defined by the treatment indication, described in the trial registration. Trials were then grouped by index condition.

For the community sample, we used codes from the Read classification system to identify people with each index condition. Read codes are a coding scheme used in UK primary care electronic health records [21]. The index condition definitions were adapted from published literature and from definitions used in the Quality & Outcomes Framework, a pay-for-performance programme which has incentivised coding for common chronic conditions (Additional file 3) [1, 22, 23]. For defining asthma, hypertension, type 2 diabetes, migraine and thromboembolic disease, prescribed medications were also used, alongside diagnostic codes, to confirm that conditions were receiving active pharmacological treatment [1].

### Quantifying comorbidities

Medical history data was frequently redacted in the trial datasets to maintain patient confidentiality, and even when provided, different terminologies were used. In contrast, all the trials providing data on concomitant medication used the World Health Organization Anatomic Therapeutic Chemical (WHO-ATC) system, the de facto standard for drug coding in clinical trials [24]. We therefore used concomitant medication data to identify 21 comorbidities in both the trial and community datasets.

Trials either reported the ATC codes directly or reported preferred terms often along with the drug route. In the latter case, we used RxNorm (the US drug metathesaurus) [25], the UK British National Formulary [21] and manual review to assign ATC codes. Trial concomitant medications were defined as any drug started on or before the randomisation date.

For the community sample, we used the NHS Business Authority ATC to Read code lookup table (as processed by the OpenPrescribing project) [26]. For drugs not found in the lookup table, we manually mapped Read code-defined drugs to ATC codes. Any drug prescribed during 2011 was included.

The following comorbidities (detailed in Additional file 4) were identified based on medication use: cardiovascular disease, chronic pain, arthritis, affective disorders, acid-related disorders, asthma/chronic obstructive pulmonary disease, diabetes mellitus, osteoporosis, thyroid disease, thromboembolic disease, inflammatory conditions, benign prostatic hyperplasia, gout, glaucoma, urinary incontinence, erectile dysfunction, psychotic disorders, epilepsy, migraine, parkinsonism and dementia. These drug-based definitions were developed in consultation with a steering committee comprising clinicians, epidemiologists and statisticians and were finalised before the analysis of the primary care data.

For each patient/participant, and within each index condition, we summed the number of individual comorbidities, not including the index condition, to obtain a comorbidity count.

### Statistical analysis

Individual-level participant data were held on the YODA repository for one trial sponsor, on the CSDR secure platform for the other trial sponsors and on the SAIL secure platform for the community sample. These platforms only allow export of non-disclosive aggregate-level data. We could not, therefore, include all individual-level data in a single model.

Therefore, for each trial, we summed the number of participants with each comorbidity count and exported this from each secure environment, along with the age-sex distribution of participants. For each indication, we obtained the number of community patients with each comorbidity count within age-sex-specific strata and directly standardised these to a weighted average of the trial age-sex distributions.

We used simulation to obtain uncertainty intervals. For single trials and community patients, we sampled from Dirichlet distributions [27]. For indications with multiple trials, we fitted a Poisson regression model, similar to a random effects meta-analysis, to the mean count. Taking posterior samples from this model, we applied the probability mass function for the Poisson distribution to obtain the proportion with comorbidity counts ranging from 0 to 12. In both cases, we obtained 1000 samples, from which we calculated the following pre-specified statistics: the ratio of mean counts of conditions, the ratio of the proportion with a count ≥ 2 and the proportion of community patients with a count greater than the trial median count. For each statistic, lower and upper uncertainty intervals were obtained as the 2.5th and 97.5th rank percentiles.

Data were prepared using Structured-query Language (SQL) and R (Vienna, Austria). The Dirichlet sampling was performed using R, and the Poisson model was fitted in Just Another Gibbs Sampler (JAGS - http://mcmc-jags.sourceforge.net/). Aggregated data and code required to run these models, along with full model descriptions, are available in Additional file 5. The statistical analysis plan, with version history, is available at https://github.com/dmcalli2/dynamic_protocols/blob/master/defining_comorbidities_SAIL.md.

Additionally, we compared data elements obtained from clinicaltrials.gov for trials where we had access to IPD and included in our analysis, to other trials for which no individual-level participant data was obtained (other trials) using descriptive statistics.

### Ethical approval

This project had approval from the University of Glasgow, College of Medicine, Veterinary and Life Sciences ethics committee (200160070). SAIL analyses were approved by SAIL Information Governance Review Panel (Project 0830).

## Results

Of the 124 trials meeting our inclusion criteria and made available via the CSDR and YODA repositories, 116 (including 122,969 participants for 22 index conditions) provided concomitant medication data allowing us to identify comorbidities. We had initially planned to include trials from the NIH BioLINCC repository, but found that none of the 8 trials which met our eligibility criteria provided sufficient data on comorbidities to be included in the analysis (Fig. 1). Index conditions are summarised in Table 1. Additional file 6 contains a summary of the characteristics of each trial. Additional file 7 shows summary statistics of the community sample for each index condition. Trials included in this analysis and trials which met our eligibility criteria but were not included (either because we did not obtain IPD or because the data we needed to perform these analyses had been redacted) were broadly similar in terms of the trial start dates, study design, excluding conditions and the number of participants enrolled as well as the clinical indications and drug classes studied (Additional file 8). However, we found that trials for inflammatory bowel disease and rheumatoid arthritis, as well as trials of immunosuppressant drugs, were somewhat overrepresented. We also found that while 11.3% of the IPD trials were phase 4 trials, 20.9% of non-IPD trials were phase 4, and that a lower proportion of IPD trials than non-IPD trials were very large (Additional file 8: Figure S8.1).

**Table 1:** Trial participants and community patients with each index condition

Index condition	Trials	Trial participants	Community-based sample
Cardiometabolic			
Type 2 diabetes mellitus	21	23,750	82,473
Atrial fibrillation	1	18,033	43,330
Hypertension	8	5151	310,691
Thromboembolism	4	9362	9162
Respiratory			
Asthma	4	1623	191,160
COPD	7	5256	57,378
Pulmonary fibrosis	2	1063	1465
Pulmonary hypertension	1	406	759
Inflammatory			
Axial spondyloarthritis	2	458	1982
Inflammatory bowel disease	12	9241	12,514
Psoriasis	7	7568	52,810
Psoriatic arthritis	3	1331	3523
Rheumatoid arthritis	11	7662	13,809
Systemic lupus erythematosus	2	1693	1033
Musculoskeletal			
Osteoarthritis	1	1320	124,521
Osteoporosis	7	14,497	38,212
Neurological			
Dementia	7	6253	13,871
Migraine	5	3069	19,562
Parkinson’s disease	3	1368	4727
Restless legs syndrome	2	676	11,480
Urological			
Benign prostatic hyperplasia	5	2210	19,906
Erectile dysfunction	1	606	65,736

**Fig. 2:**
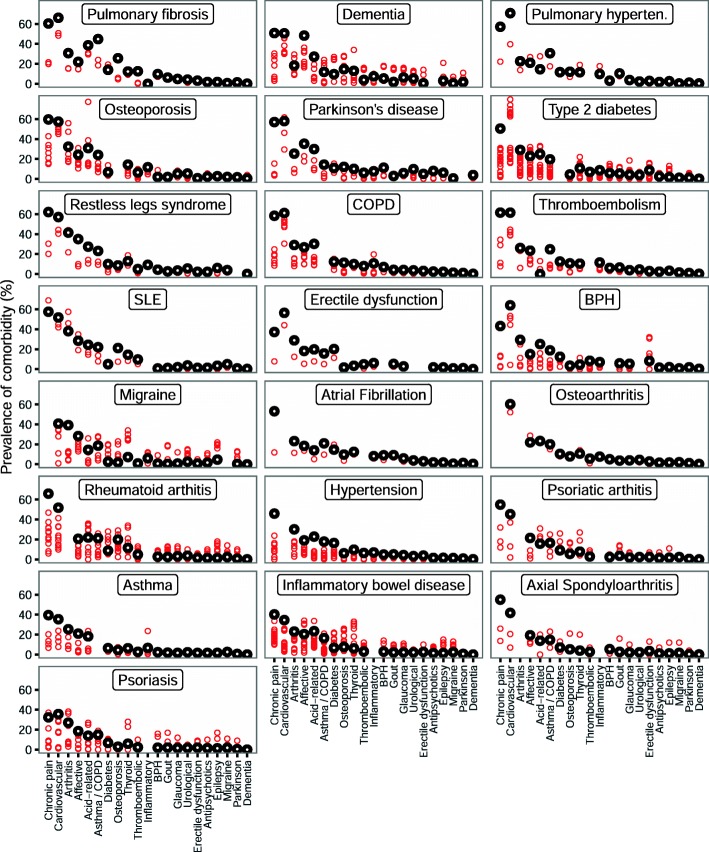
Scatterplot of the prevalence for each comorbidity for each index condition, for the community-based sample and for clinical trial participants. Black circles indicate the community-based cohort and red circles trials. The *x*-axis is sorted according to the prevalence of the comorbidities in the community-based sample. The sort order was obtained by ranking the comorbidities from commonest to least common for each index condition, then by taking the median across all index conditions. The individual panels are sorted by the mean comorbidity count for each index condition, from highest to lowest. Where the index condition was judged to be the same as the comorbid condition, the comorbidity was not defined, which accounts for apparently missing points on the graph. So, for example, for people in the community sample who had migraine, the most common comorbidity was chronic pain with the next most common being cardiovascular disease

For each index condition, most comorbidities were more common in community patients than in the trials (Fig. 2). In community patients, the seven commonest comorbidities, from most to least common, were chronic pain, cardiovascular disease, arthritis, affective disorders, acid-related disorders, asthma or COPD, and diabetes. These conditions were common across all index conditions, although the ordering varied somewhat. For example, cardiovascular disease was commoner than chronic pain for both type 2 diabetes and COPD. This difference in ordering was evident for *both* the community sample and the trials. Indeed, for most index conditions, those comorbidities which were commonest in the community were also commonest for the trials.

For each of the comorbidities assessed, prevalence varied between trials. Some trials had a prevalence close to that of the primary care population, while in other trials the prevalence was much lower (Fig. 2 and Additional file 9). This pattern was similar across all index conditions, and for all comorbidities assessed. No specific comorbidities stood out as being consistently underrepresented. Conversely, none was found to be well represented across all trials.

**Fig. 3:**
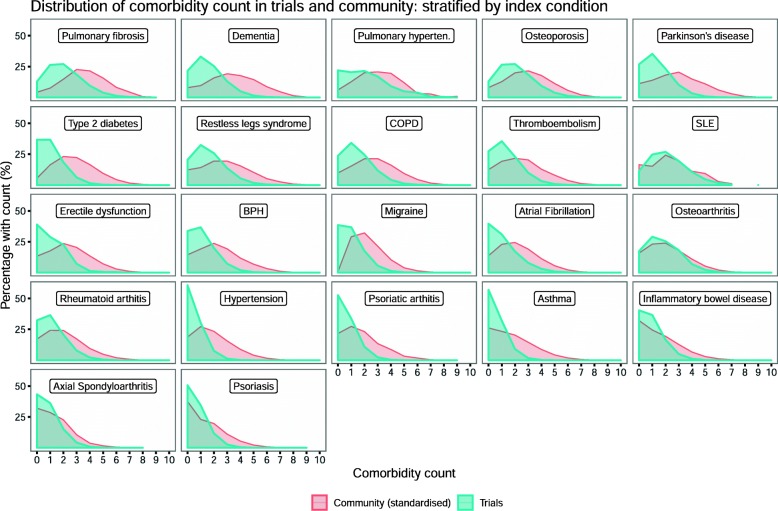
Proportion with each comorbidity count in trials and community: stratified by index condition. This plot indicates the proportion of comorbidity counts for each index condition. The height of the plot indicates the percentage of participants/patients with a particular count for each index condition. For community-based patients, the proportion of patients with each comorbidity count has been standardised to the trial populations; this was done by applying age-sex-specific proportions to the age-sex distributions of the trial participants. For the trial participants, where there were multiple trials per condition, the proportion with each comorbidity was obtained from the modelled mean comorbidity counts for each index condition (see Table 2), under the assumption that the proportion of trial participants with each comorbidity count follows a Poisson distribution. Where there was only a single trial for a given condition (e.g. osteoarthritis), raw proportions are given. See Additional file 5 for further details of these analyses

Figure 3 shows the distribution of the comorbidity counts for trial participants and community-based patients. For each index condition, the comorbidity distribution for community-based patients lay to the right of the trial distribution (i.e. more comorbidities in community patients compared to trial participants). The community-based counts were *standardised* to the age-sex distributions of the trial participants for the relevant condition. However, the standardisation made little difference to the estimates (Additional file 10) so only the age-sex standardised results are presented. For the trial participants, where there were multiple trials per condition, the proportions were obtained from the *modelled* mean comorbidity counts for each index condition (see Table 2), under the assumption that the proportion of trial participants with each comorbidity count follows a Poisson distribution. Where there was only a single trial for a given condition (e.g. osteoarthritis), raw proportions are shown (see Additional file 5 for details). Comorbidity counts varied by index condition. Lower counts were evident for conditions such as asthma, inflammatory bowel disease and psoriasis. Conditions with higher comorbidity counts were those with a later age of onset. For most index conditions, the mean comorbidity counts were between 1.5-fold higher and 3-fold higher for community-based patients than for trial participants (Table 2).

**Table 2:** Comorbidity counts in trial participants and in the community, ordered according to the mean comorbidity counts in the community

Indication	Mean comorbidity count			% with comorbidity count > 2	
	Community (standardised to trials)	Trials	Ratio between mean counts (community:trials)	Community	Trials
Pulmonary fibrosis	3.61 (3.49–3.75)	2.17 (1.03–3.92)	1.87 (0.90–3.49)	0.88 (0.86–0.91)	0.61 (0.28–0.90)
Dementia	3.44 (3.38–3.49)	1.56 (1.10–2.18)	2.27 (1.58–3.11)	0.82 (0.81–0.83)	0.46 (0.30–0.64)
Pulmonary hypertension	3.27 (3.01–3.53)	2.09 (1.93–2.26)	1.57 (1.39–1.75)	0.80 (0.75–0.84)	0.58 (0.53–0.63)
Osteoporosis	3.01 (2.99–3.03)	2.07 (1.43–2.95)	1.50 (1.02–2.10)	0.79 (0.79–0.80)	0.60 (0.42–0.79)
Parkinson’s disease	3.00 (2.90–3.10)	1.36 (0.78–2.18)	2.37 (1.37–3.86)	0.75 (0.73–0.77)	0.39 (0.18–0.64)
Type 2 diabetes mellitus	2.95 (2.94–2.96)	0.79 (0.43–1.32)	4.06 (2.23–6.83)	0.78 (0.78–0.78)	0.27 (0.19–0.36)
Restless legs syndrome	2.85 (2.81–2.89)	1.68 (0.83–2.98)	1.89 (0.96–3.42)	0.74 (0.73–0.75)	0.48 (0.20–0.80)
Pulmonary disease, chronic obstructive	2.76 (2.75–2.78)	1.46 (1.00–2.07)	1.96 (1.33–2.78)	0.75 (0.74–0.75)	0.43 (0.26–0.61)
Thromboembolism	2.52 (2.48–2.56)	1.33 (0.80–2.00)	2.00 (1.24–3.13)	0.68 (0.67–0.70)	0.38 (0.19–0.59)
Systemic lupus erythematosus	2.51 (2.35–2.68)	2.30 (1.08–4.12)	1.22 (0.60–2.32)	0.69 (0.65–0.73)	0.63 (0.29–0.92)
Erectile dysfunction	2.44 (2.43–2.45)	1.08 (0.99–1.17)	2.27 (2.08–2.47)	0.69 (0.69–0.69)	0.32 (0.29–0.36)
Benign prostatic hyperplasia	2.37 (2.34–2.40)	1.11 (0.70–1.69)	2.24 (1.40–3.36)	0.66 (0.66–0.67)	0.30 (0.16–0.50)
Migraine	2.33 (2.31–2.35)	0.98 (0.63–1.42)	2.50 (1.65–3.73)	0.70 (0.69–0.71)	0.26 (0.13–0.42)
Atrial fibrillation	2.22 (2.20–2.23)	1.08 (1.07–1.10)	2.05 (2.01–2.08)	0.63 (0.63–0.64)	0.29 (0.29–0.30)
Osteoarthritis	2.14 (2.13–2.15)	1.79 (1.72–1.86)	1.20 (1.15–1.24)	0.61 (0.61–0.61)	0.54 (0.51–0.56)
Rheumatoid arthritis	2.07 (2.03–2.10)	1.14 (0.87–1.51)	1.84 (1.37–2.39)	0.59 (0.58–0.60)	0.32 (0.22–0.44)
Hypertension	1.91 (1.90–1.91)	0.50 (0.36–0.69)	3.88 (2.76–5.28)	0.54 (0.54–0.54)	0.09 (0.05–0.15)
Psoriatic arthropathy	1.84 (1.78–1.89)	0.67 (0.38–1.09)	2.96 (1.71–4.88)	0.51 (0.50–0.53)	0.15 (0.06–0.30)
Asthma	1.81 (1.81–1.82)	0.58 (0.35–0.92)	3.32 (1.97–5.24)	0.51 (0.50–0.51)	0.12 (0.05–0.23)
Inflammatory bowel disease	1.55 (1.52–1.58)	0.92 (0.68–1.18)	1.73 (1.32–2.28)	0.44 (0.43–0.45)	0.23 (0.15–0.33)
Axial spondyloarthritis	1.42 (1.34–1.50)	0.88 (0.43–1.58)	1.79 (0.90–3.31)	0.40 (0.37–0.43)	0.22 (0.07–0.47)
Psoriasis	1.35 (1.34–1.37)	0.69 (0.48–0.99)	2.02 (1.36–2.80)	0.39 (0.39–0.40)	0.15 (0.08–0.26)

Nonetheless, in absolute terms, comorbidity was common in both settings (Table 2). Most community-based patients had two or more comorbidities (i.e. three or more conditions overall) and would therefore be considered to have a high degree of multimorbidity under many definitions [28]. In trials, a significant proportion also had two or more comorbidities. This ranged from 10 to 15% for conditions such as asthma and psoriasis to around 40–60% for conditions with an older age of onset such as osteoporosis, dementia and pulmonary fibrosis.

**Fig. 4:**
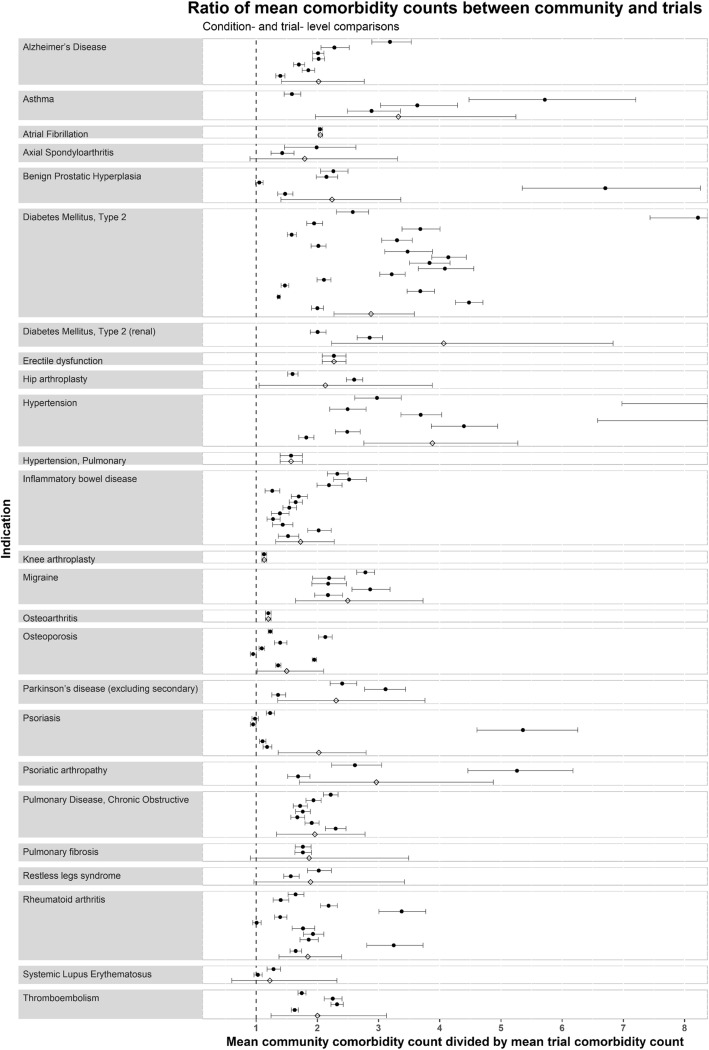
Ratio of mean comorbidity counts between community and trials: condition- and trial-level comparisons. Points represent the ratio of mean count between community patients and trials, and the bars represent 95% credible intervals. Trial estimates are represented by solid circles, and index-condition-level meta-estimates are represented with empty diamonds. The ratio represents the mean community comorbidity count for that index condition, divided by the mean trial comorbidity count, i.e. value of 1 indicates no difference in mean comorbidity count, value of 2 indicates the mean comorbidity count is twofold higher in community than in trials, etc. An interactive version of this figure, with links to the clinicaltrials.gov registration for each trial, is shown in Additional file 12

On examining individual trials, the mean comorbidity count was the same or higher in the community than for every trial (Fig. 4). Nonetheless, there was considerable variation, even within the same index conditions. For some trials, the mean comorbidity counts were almost the same as in the community; for others, there was more than a twofold difference. In additional analyses, to explore this variation, we plotted the mean comorbidity count for each trial against trial-level characteristics such as the start date, phase, sponsor and total number of excluding conditions within the eligibility criteria, without observing any associations (Additional file 11).

## Discussion

We examined comorbidity and multimorbidity using individual-level participant data from 116 trials (122,969 participants) from seven industry sponsors of novel drug treatments for 22 common index conditions. We assessed the same comorbidities for the same index conditions from a nationally representative community sample of 2.3 million people. Comorbidity and multimorbidity were consistently lower in trial populations than in community patients, but were nonetheless common in both.

Our estimates of comorbidity in the community are consistent with previous findings: comorbidity was common, and for some conditions (e.g. COPD and osteoporosis), it was almost ubiquitous [1, 28]. To our knowledge, however, ours is the first study to compare comorbidity and multimorbidity patterns in the community to those in clinical trial populations by directly analysing comorbidity counts using individual-level trial participant data. In so doing, we confirmed that the mean comorbidity count for trials was approximately half that observed in the community.

We also found that, although patients with comorbidity or multimorbidity were underrepresented in many trials, comorbidity and multimorbidity were nonetheless common. For around half of the index conditions, the proportion of trial participants with ≥ 2 comorbidities (i.e. with three conditions and therefore highly multimorbid [28]) was above 30%. Given the ubiquity of multimorbidity among patients in the community [1, 28], it is perhaps unsurprising that comorbidity and multimorbidity are so common in industry-funded trials of novel drugs. However, we do not think that this unexpectedly high prevalence has previously been noted.

This finding is important because of current uncertainty as to the treatment of people with multimorbidity. Guidelines on the treatment of multimorbidity express reservations about the applicability of trial evidence to people with multimorbidity [29]. Moreover, in clinical practice, people with comorbidity (who, by definition, have multimorbidity) are less likely to receive certain drug treatments recommended across a range of disease-specific guidelines [2–5]. Our findings that comorbidity and multimorbidity are underrepresented in clinical trials would support a cautious approach by guideline developers to the routine extrapolation of evidence. However, the finding that comorbidity and multimorbidity are common in clinical trials is important, because it suggests that trial data could potentially provide an important resource to allow treatment effects to be estimated in people with multimorbidity. These findings have implications for both triallists and for guideline developers.

The first implication for triallists and guideline developers relates to making better use of existing evidence. One way of doing so is via individual participant-level data meta-analyses. For this reason, we agree with the Alltrials initiative, and others, that sharing of IPD from clinical trials is crucial. Such analyses have helped resolve previous controversies about the efficacy of drugs in different sub-groups, showing, for example, that aspirin is similarly efficacious in men and women [30–34]. Similar analyses have the potential to resolve similar controversies concerning comorbidity and multimorbidity [29, 35], potentially changing clinical practice, either by providing reassurance that trial findings can be applied to people with multimorbidity or by providing robust evidence to the contrary.

However, compared to meta-analysis of published results, IPD meta-analysis is costly and challenging. If trials are to be widely used to inform clinicians and guideline developers as to the efficacy of different treatments in the presence of comorbidity or multimorbidity, trials must publish results according to comorbidity sub-groups. Doing so will be challenging, however, because there are multiple different potential patterns of comorbidity. This is true even if only a small number of comorbid diseases are considered. There are, for example, 64 different possible ways that six conditions can occur together. Whether important and clinically relevant patterns of comorbidity can be identified from among such combinations remains an active and unresolved research question [36]. Nonetheless, we found that those comorbidities which were common in the community were also common in trials. Consequently, if clinically meaningful patterns of comorbidity and multimorbidity can be identified among people in the community, it may be possible to identify similar sub-groups among trial participants.

In the absence of consensus on which patterns of comorbidity should be grouped together, we propose that trials report treatment effects according to the presence/absence of common comorbidities, as well as by multimorbidity counts. Ideally, comorbidities would be defined using medical history data collected in a systematic and standardised manner across trials. In the absence of standardised medical histories [9, 37], some insights may be obtained from existing trials using drug-defined comorbidities, particularly where the focus is on conditions closely associated with particular drug classes (e.g. diabetes and glucose-lowering drugs) or on overall measures of multimorbidity, such as a count.

Despite these challenges, using clinical trial data to estimate treatment effects in people with comorbidity or multimorbidity remains appealing because of limitations in the alternatives. For example, observational datasets rich in multimorbidity, such as electronic health records, are used to estimate treatment effects. However, despite methodological advances in this use of observational data, it remains controversial, as unmeasured confounding can result in apparent treatment benefits when none really exist [9, 38].

The second implication for triallists relates to eligibility criteria and recruitment. For many indications, there was little difference in comorbidity counts between some trials and the community sample, whereas for other trials within the same indication the differences were large. This suggests that, even for standard industry-funded phase 3/4 trials, increasing the recruitment of comorbid participants is feasible. There is therefore potential for future trials to become more representative in terms of multimorbidity. In exploratory analyses, the differences in comorbidity between trials for similar indications were not related to start date, phase, sponsor or total number of exclusion criteria. Additional work is needed to identify the selection processes driving inclusion or exclusion of people with comorbidity so that trials can be made more representative. In addition, it will be important for future research to examine how conditions cluster in people with multimorbidity and whether this differs between clinical trial participants and people in the community in order to improve analysis and reporting of treatment effects as well as trial design.

The strengths of our study include large numbers and that the comorbidity definitions and analyses were pre-specified before making comparisons. However, there are several limitations. First, the trials collected medical history data in a variety of incommensurable ways. Consequently, we used concomitant medications to define comorbidities. This meant that some important conditions that are not treated with specific medications (e.g. chronic kidney disease) could not be identified reliably, whereas some other conditions which share treatments (e.g. asthma and COPD) had to be combined into broader categories. The use of some medications was so heterogenous as to preclude meaningful categorisation, and we did not attempt to use such drugs in any definition (for example, since amitriptyline is widely used in the treatment of chronic pain [39], we did not include it in our definition of affective disorders). Despite these limitations, some conditions are well defined by medications, and importantly, the same definitions were applied across trial and community data. Our community sample was taken from Wales because, while being broadly similar to the rest of the UK, it provides access to electronic medical records from a large and representative sample covering 70% of the population [40]. The Welsh population is broadly similar to the UK population in demography, and the findings are likely to be applicable to other high-income countries, but do require replication in other contexts. In order to facilitate this, we provide standard comorbidity definitions as well as data on the distribution of comorbidity counts, age and sex at the level of individual trials. A further limitation is that the included trials were not a random sample of all trials for these index conditions. Not all sponsors share trial data. Those who do share data do not make all trials available. Differences between trials that do or do not provide IPD may be a potential source of bias [41]. As such, we believe that the sharing of data by trial sponsors is to be encouraged, so as to minimise bias arising from the availability of a limited set of trials. Nonetheless, the included trials were similar to a wider body of registered trials across a range of characteristics (Additional file 8).

## Conclusion

Clinical trial populations have a lower prevalence of comorbidity and multimorbidity than unselected community populations. Clinicians should exercise caution when applying disease-specific evidence and guidelines to people with comorbidity or multimorbidity. Nonetheless, comorbidity and multimorbidity are common in clinical trials. Given the limitations of observational data for estimating treatment effects, this suggests that standard industry-funded clinical trials are an underused resource for estimating treatment effects in multimorbidity. We would recommend that future disease-specific guidelines need to incorporate information concerning likely treatment effects in the context of the specific index condition and comorbidity or multimorbidity. To enable guideline developers to do so, triallists should at least report the prevalence of multimorbidity and a range of comorbidities among trial participants and should consider reporting treatment effect estimates stratified by comorbidity and/or multimorbidity. More general multimorbidity guidelines could also usefully include information in relation to this within any future guideline to permit more specific guidance for clinicians dealing with people with multimorbidity.

## Supplementary information

**Additional file 1.** Selection-of-trials-protocol.pdf: Protocol for selection of clinical trials individual participant data and search of wider body of registered trials from clinicaltrials.gov.

**Additional file 2.** Representativeness-of-community-data-sail.pdf: Analysis of the representativeness of the community sample.

**Additional file 3.** Selection-of-patients-and-participants-from-primary-care-data-read-codes.pdf: Read codes used to identify index conditions.

**Additional file 4.** Defining-comorbidity-protocol.pdf: Protocol detailing the identification of comorbidities from clinical trial data.

**Additional file 5.** More-detailed-statistical-analysis.pdf: Model description and code required for analyses. Detailed description of statistical methods.

**Additional file 6.** Trials-characteristics.pdf: Summary of characteristics of included trials.

**Additional file 7.** Characteristics-of-primary-care-populations-with-each-of-the-trial-indications.pdf: Summary statistics of community sample for each index condition.

**Additional file 8.** Summary-Statistics-Comparing-Ipd-Trials-To-Wider-Body-Of-Trials-From-Clinicaltrials.Gov.Pdf: Comparison of included trials with registered trials on clinicaltrials.gov for which individual participant data were not available.

**Additional file 9.** Proportion-with-each-comorbidity-for-trials-and-sail.pdf: Analysis of the prevalence of each comorbidity, within each index condition, in trial participants and the community sample.

**Additional file 10.** Comorbidity-counts-for-trials-and-primary-care.pdf: Summary comorbidity counts.

**Additional file 11.** Explore-relationship-of-trial-mean-comorbidity-counts-to-trial-characteristics.pdf: Analysis of characteristics based on trial meta-data and relationship to comorbidity counts.

**Additional file 12.** Figure-4-interactive.svg: Interactive version of Fig. 4 with hyperlinks to trial registration.

## Data Availability

All data released from the respective safe havens (YODA, CSDR and SAIL) has been made available via the supplementary appendix. Potentially disclosive data can be accessed by applying to the original data holders who were reported in the “Methods” section.

## Abbreviations

COPD: Chronic obstructive pulmonary disease
CSRD: Clinical Study Data Request
IPD: Individual-level participant data
SAIL: Secure Anonymised Information Databank
WHO-ATC: World Health Organization Anatomic Therapeutic Chemical classification
YODA: Yale University Open Data Access
