# Supplementary material for: Representation of people with comorbidity and multimorbidity in clinical trials of novel drug therapies: an individual-level participant data analysis
Source: BMC Med. 2019 Nov 12;17:201. doi: 10.1186/s12916-019-1427-1 (PMC6849229; doi:10.1186/s12916-019-1427-1)
Supplement: Supplementary file 1 — Additional file 1. Selection-of-trials-protocol.pdf: Protocol for selection of clinical trials individual participant data and search of wider body of registered trials from clinicaltrials.gov. [file 12916_2019_1427_MOESM1_ESM.pdf]

Representation of people with comorbidity in clinical trials of novel drug therapies; an individual-level participant data analysis

11 July, 2019

1 Selection of trials - Protocol

The search strategy and selection process for the individual-level participant data (IPD) trials included in this analysis were pre-registered via PROSPERO. The record is available at https://www.crd.york.ac.uk/PROSPERO/display_record.php?RecordID=48202. For convenience, the relevant sections of the protocol as submitted with that application are reproduced below.

1.1 Overview

Trials were chosen that enrolled a minimum of 300 participants, had no maximum age or a maximum age of >= 60, and included drugs used to treat or prevent long term medical (and, as these are a common issue in the elderly, urological) conditions. Both short and long-term indications arising within the context of long-term conditions were included (e.g. short-term post MI therapy). We excluded trials where the treatment was for neoplastic, infectious, affective, psychotic or developmental disorders. We did not exclude trials where patients had cancer and were being treated with, for example, antithrombotic drugs to prevent DVT or PE. Topical eye and primary prevention trials in the general adult population were also excluded.

The complete list of included drugs is listed in the relevant sections below. Initially we included drugs acting on male and female sex hormones as these are used to treat certain chronic diseases. However, in view of the widespread use of the latter in treating cancer, and as a contraceptive and for the symptoms of menopause – we excluded these before obtaining any individual-level patient data (IPD), as these conditions fall outside our scope.

Representative samples of individual-level patient data were not available as access to IPD remains incomplete; not all sponsors share trial data, and among those that do, not all trials are shared. Therefore, rather than a representative sample we aimed to obtain a set of relevant trials. Therefore, we searched for trials within three repositories for which clinical trial data are available – the National Institutions of Health NIH BioLINCC repository (https://biolincc.nhlbi.nih.gov/home/), the multi-sponsor Clinical Study Data Request (CSDR) repository (https://www.clinicalstudydatarequest.com/) and the Yale University Open Data Access (YODA) Project (http://yoda.yale.edu/). We then performed a search within clinicaltrials.gov, the U.S. National Library of Medicine Clinical Trial Register (https://clinicaltrials.gov/), to obtain a “denominator” set of trials, of which the trials identified by screening the repositories can be considered a subset.

Any repository trial which was not found on applying our search criteria to the registry was excluded. In this way, while the set of trials for which we have IPD cannot be said to be representative (in the sense of a random or complete sample), it can be related to a wider body of trials in a public registry.

1.2 Clinical Study Data Request – trial repository

Using the search tool provided within the CSDR repository, we obtained a list of all phase 2/3, 3 and 4 trials on CSDR on 2016/11/21. This identified 1821 trials for which we downloaded a short trial description.

As in the following example, each trial description contained the trial title, sponsor, medicine, condition, phase and additional study ID:-

- sponsor_id: ASTELLAS-E05-CL-3002
- title: A Randomized, Controlled, Long-term Safety Study Evaluating the Effect of Repeated Applications of QUTENZATM plus Standard of Care versus Standard of Care alone in Patients with Painful Diabetic Peripheral Neuropathy (PACE)
- medicine: capsaicin
- condition: Painful diabetic neuropathy
- phase: 3
- clinical study id: E05-CL-3002

There were 292 unique condition strings. These did not appear to be from a controlled vocabulary, and so each was reviewed manually and assigned into one of 25 groups (Table S1.1).

Table S1.1: Conditions as recorded on CSDR site

Selected	Type of condition	Conditions
No	aesthetic	Alopecia
No	affective	Acute Mania, Anxiety Disorder, Anxiety Disorders, Attention Deficit Hyperactivity Disorder, Major Depressive Disorder, Oppositional Defiant Disorder, Bipolar Disorder, Depressive Disorder, Major, Dermatitis, Atopic, Insomnia, Mood Disorders, Obsessive-Compulsive Disorder and Depressive Disorder, Obsessive-Compulsive Disorder, Panic Disorder, Post-Traumatic Stress Disorder, Schizophrenia, Social Phobia, Treatment-Resistant Depression
Yes	bone	osteoporosis with pathological fracture, Osteoporosis, Male, Hip Fracture
Yes	cancer	Breast Cancer, Breast Neoplasms, Cancer , Cancer, Advanced Gastric, Breast, Carcinoma, Non-Small-Cell Lung, Adenocarcinoma, Renal Cell, Chronic Lymphocytic Leukemia, Colonic Neoplasms, Differentiated Thyroid Cancer, Erosive Esophagitis, Gastroesophageal Reflux Disease, gastric cancer stomach neoplasms, Gastric Cancer, Gastric cancer/ Stomach neoplasms, Head and Neck Neoplasms, Squamous Cell, HER2-positive metastatic breast cancer, HER2-positive metastatic gastric cancer, locally advanced metastatic or recurrent non-squamous non-small cell lung cancer, Lymphoid Leukaemia, Lymphoma, Non-Hodgkin, malignant neoplasm breast unspecified site, malignant neoplasm of breast, Malignant Neoplasm Of Bronchus And Lung, malignant neoplasm of colon/rectum, malignant neoplasm of colon, malignant neoplasm of rectum, Malignant Neoplasm Of Lung, malignant neoplasm of ovary, malignant neoplasm of pancreas, Mesothelioma, metastatic colorectal cancer, Multiple myeloma (MM), Myelodysplastic syndrome (MDS), Neoplasms, Prostate, Non-Small Cell Lung Cancer, Non-small cell lung cancer, Nonsquamous non-small cell lung cancer (NSCLC). , Nonsquamous

Table S1.1: Conditions as recorded on CSDR site

Selected	Type of condition	Conditions
		Non-Small Cell Lung Cancer (NSCLC). , Ovarian Cancer, refractory metastatic breast cancer, renal cell carcinoma, Risk Reduction of Invasive Breast Cancer in Postmenopausal Women, Cardiovascular Disease, Sarcoma, Soft tissue, Urothelium Cancer
Yes	Chronic lower respiratory disease	Asthma, Bronchitis, Chronic, Bronchospasm, Chronic Obstructive Pulmonary Disease (COPD), Pulmonary Disease, Chronic Obstructive, Ph, Asthma
Yes	Cardiovascular disease	Acute Coronary Syndrome, acute coronary syndrome, Acute Ischemic Hemispheric Stroke, acute ischemic stroke, Atherosclerosis, Atrial Fibrillation, Stroke, Cardiovascular Diseases, Cerebrovascular Accident, Coronary Arteriosclerosis, Fibrillation, Atrial, Heart Arrest, Heart Failure, Chronic (CHF), Heart failure, Congestive and Microalbuminuria, Congestive, Hypertension, Pulmonary, Diabetes Mellitus, Type 2, Diabetic Nephropathies, Hypertriglyceridemia, Hypertrophy, Left Ventricular, Myocardial Infarction, Myocardial Perfusion Imaging, Non-valvular Atrial Fibrillation, Obesity, Atrial Fibrillation, Embolism
Yes	Dementia	Alzheimer’s Disease, Dementia
Yes	Dermatological	Acne Vulgaris, chronic idiopathic urticaria (CIU) , Chronic Idiopathic Urticaria (CIU)
No	Developmental	Attention Deficit Hyperactivity Disorder, Turner Syndrome
Yes	Diabetes	Diabetes Mellitus, Diabetes Mellitus, Type 1, Type 2 , Type 2, type 2, Hyperglycemia, Hypertension, Renal Insufficiency, Diabetic Eye Disease, Diabetic Nephropathies, Diabetic Neuropathies, Depressive Disorder, Major, Diabetic Peripheral Neuropathic Pain, Neuropathy, Diabetic, Painful diabetic neuropathy, Type 2 Diabetes Mellitus
Yes	Endocrine	Hypogonadism
Yes	Epilepsy	Epilepsy, Epilepsy, Partial, Tonic-Clonic, Lennox-Gastaut Syndrome, Seizures
No	ophthalmological	Allergic conjunctivitis, Neovascular Age-related Macular Degeneration, Open-Angle Glaucoma, Ocular Hypertension, Retinal Vascular Occlusion
No	infection	Acellular pertussis, Diphtheri, Acellular pertussis, Diphtheria, Haemoph, Haemophilus in, Haemophilus influenz, Haemophilus influenza, Haemophilus influenzae, Haemophilus influenzae typ, Haemophilus influenzae type b, He, Hepa, Hepatiti, Hepatitis B, Pol, Poliomyelitis, Tetanus, acellular pertussis, Tet, Phas, Acquired Immunodeficiency Syndrome, chronic hepatitis b, chronic hepatitis B, chronic hepatitis c, Whol, Whole Cell Pertussis, Phase, Acellular Pertussis, Neisseria Meningitidis, Hepatitis A, Chronic, Hepatitis C, Herpes Genitalis, Herpes Labialis, Herpes Simplex, hiv disease, HIV Infections, Hepatic Insufficiency, Metabolism, Lipids, Impetigo, Infection, Human Immunode, Human Immunodeficiency Virus I, Human Immunodeficiency Virus, Infections, Herpesviridae, Meningococcal, Papillomavirus, Rotavirus, Streptococcal, Influenza infection, Influenza, Human, influenza, Invasive aspergillosis / Invasive mucormycosis, Invasive Fungal Infections, Malaria, Falciparum, Measles, Mumps, Rubella , Rubella, Varicella, Otitis Maedia, Pharyngitis, Pneumonia, Community-Acquired, Severe Sepsis, Skin Infections, Bacterial, Streptococcus pneumoniae, Diphth, Therapeutic treatment of inhalation anthrax, Typhoid Fever, viral diseases

Table S1.1: Conditions as recorded on CSDR site

Selected	Type of condition	Conditions
Yes	inflammatory	Crohn's Disease, Psoriasis, Psoriatic Arthritis, Ulcerative Colitis, Crohn's Disease
Yes	liver	Liver Diseases
Yes	neurological	Dermatitis, Chronic, Dermatitis, Seborrheic, Idiopathic Restless Legs Syndrome, Migraine Disorders and Premenstrual Syndrome, Migraine Disorders, Migraine, Without Aura, Multiple Sclerosis, Parkinson Disease, Depression, Parkinson's Disease Dementia, Peripheral Neuropathies, Restless Legs Syndrome, Tourette Syndrome
No	not suitable, other	Central Venous Access Devices, Healthy Subjects, Premenstrual Syndrome
Yes	pain	Analgesia, Chronic Lower Back Pain, Fibromyalgia, IrriTable Bowel Syndrome with constipation (IBS-C), Neuropathic pain, Osteoarthritis Knee Pain, Osteoarthritis, pain, not elsewhere classified
Yes	rheumatological	ankylosing spondylitis , Arthritis, Juvenile Rheumatoid, Rheumatoid, Axial Spondyloarthritis , Lupus, rheumatoid arthritis (juvenile), Rheumatoid Arthritis, rheumatoid arthritis, Systemic Lupus Erythematosus
Yes	Risk-factor	Smoking Cessation
Yes	suitable, other	Erectile Dysfunction, Fluoxetine-Associated Sexual Dysfunction, Hyperuricemia, Polyps, Nasal, Pulmonary Fibrosis, Purpura, Thrombocytopaenic, Idiopathic, Rhinitis, Allergic, Perennial and Seasonal, Perennial, Seasonal, Vasomotor, Sinusitis
Yes	surgical	Arthroplasty, Replacement, Knee, Thromboembolism, Arthroplasty, Thromboembolism, Prevention of Venous Thromboembolism, Moderate Renal Impairment (CrCl 30-50 mL/Min), Benign Prostatic Hyperplasia, Catheter clearance, Constipation, Erectile Dysfunction, Femoral Neck (Hip) Fracture, Lower Urinary Tract Symptoms / Benign Prostatic Hyperplas, Lower Urinary Tract Symptoms / Benign Prostatic Hyperplasia, , Prostatic Hyperplasia, Urinary Bladder, Overactive, Urinary Incontinence, Stress, Venous Insufficiency
Yes	Thromboembolic	Embolism, Pulmonary, Secondary prevention of Thromboembolic events, Thrombosis, Venous, Thromboembolism, Arthroplasty, Replacement, Hip, Thromboprophylaxis, Venous Thromboembolism, Deep Vein Thrombosis (DVT), Pulmonary Embolism (PE), Thromboembolism, Venous Thrombosis
Yes	Transplant	Heart transplantation, Kidney Transplantation, Liver Transplantation, Transplantation, Kidney, Liver

We initially excluded trials where the condition was classified as aesthetic, affective, developmental, eye-disease, or infectious or "other-unsuitable". Of the latter, the conditions being treated were "Central Venous Access Devices", "Healthy Subjects" and "Premenstrual Syndrome". On applying these exclusion criteria, 850 trials remained.

For each of these, the trial drug was assigned to a unique concept ID from the RXNORM database and a label from the World Health Organisation (WHO) Anatomic Therapeutic classification (ATC). Where a drug did not yet have a WHO ATC label, it was assigned to the closest class. Each class was reviewed and trials with the following drug-classes were excluded - A01AD, A06AG, A06AX, D01AE, D05AX, D07AD, D10AE, D10AF, G01AC, D07AC, D11AH, G01AF, G02CC, G01AA, J01CA, J02AB, J01CR, J01FF, M02AA, M02AA, M02AB, N01BX, N05AH, N06AB, N06AX, R01AD, R01AX, S01BC, S01LA, S01XA, S01GX, V10XA.

On applying these exclusion criteria, 615 trials remained.

Drugs have a one-to-many relationship with ATC classes, so some trials with drugs in excluded classes were nonetheless retained (eg corticosteroid drugs can be assigned to one of several classes depending on the route of administration).

To exclude trials of anti-cancer therapies, and trials of immune-suppression therapy for transplantation, we then excluded trials where the condition was cancer or transplantation, and the drug was in the WHO ATC antineoplastic and immunomodulating drugs class (L), the Testosterone-5-alpha reductase inhibitors class (G04CB) or the sex hormones and modulators of the genital system class (G03). We did not exclude all trials with these drugs in order to retain trials of, for example, the use of immunomodulating drugs for inflammatory arthropathies.

On applying these exclusion criteria, 541 unique trials remained.

We then identified which of the trials had been registered in clinicaltrials.gov. 152 were not registered (142 of these were started before the requirement to register). On excluding un-registered trials 382 trials remained.

Of these, the following were excluded based on information obtained from clincialtrials.gov (Table S1.2).

Table S1.2: CSDR trials excluded based on additional eligibility criteria

Exclusion criteria	Number of trials excluded
Enrolment < 300	131
Excludes older people	24
Did not meet design criteria	94
Any exclusion	177

On applying these exclusion criteria 205 trials remained. On reviewing these, two were subsequently excluded based on the indication (1 for erectile dysfunction, 1 for delayed growth).

We subsequently obtained detailed description data for each trial from the CSDR repository. We found that one trial (BI-122.56) was no longer listed on the repository and so it was removed, leaving 202 trials. After applying to CSDR for these trials we removed one clinical trial of tencteplase in out of hospital cardiac arrest (BI-1123.18) and added one trial of Pramipexole for Parkinson's disease (BI-248.525) following advice from the trial sponsor.

These 202 trials included a total of 312,922 participants.

After examining the US clinical trials register, we reduced the number of eligible conditions which resulted in excluding 3 additional trials as unsuitable for analysis (2 were trials of antiemetics for post-operative nausea and vomiting (NCT00334152 and NCT00326248) and 1 was for a selective estrogen receptor modulator (NCT00670319). In the case of the latter trial, this was excluded because the inclusion of this trial would have led to a much larger dataset including large numbers of trials for unsuitable indications. This is because of the large number of trials for drugs acting on the female sex-hormone axis for contraception, anti-cancer therapy and/or the symptomatic management of menopause.

This further restriction resulted in a total of 199 trials.

1.3 YODA

We performed a search of the YODA trial repository on 18th May 2017. We searched the repository for trials with the following drug categories (Table S1.3)) or condition categories (Table S1.4).

Table S1.3: YODA Selected drug categories

Drug class	Selected
Alzheimer's Disease - Cholinesterase Inhibitors	Y
Anticonvulsants	Y
Antimycobacterial agents	

Table S1.3: YODA Selected drug categories

Drug class	Selected
Antipsoriatics	Y
Antirheumatic agents - biologic response modifiers	Y
Antiviral Agent	
Atypical antipsychotics	
Cardiovascular devices	
Colony-stimulating factors	Y
Diabetes Related- Other	Y
Hormones	Y
Immunizations	
Oncology - Antibiotic	
Orthopedic device	
Quinolones - 3rd gen.	
Stimulants/ADHD/Anorexiants	

Table S1.4: YODA Selected condition categories

Disease	Selected
Anemia	
Arthritis, Juvenile	
Arthritis, Psoriatic	Y
Arthritis, Rheumatoid	Y
Atrial Fibrillation	Y
Attention Deficit and Disruptive Behavior Disorders	
Attention Deficit Hyperactivity Disorder	
Autistic Disorder	
Bipolar Disorder	
Colitis, Ulcerative	Y
Conduct Disorder	
Critical Illness	
Crohn’s Disease	Y
Dementia	Y
Depressive Disorder, Major	
Diabetes Mellitus, Type 2	Y
Epilepsy	Y
Hepatitis C	
HIV Infections	
Leukemia	
Migraine Disorders	Y
Multiple Myeloma	
Neoplasms	
Neoplasms, Breast	
Neoplasms, Ovarian	
Neoplasms, Prostatic	
Partial Seizure Disorder	Y
Psoriasis	Y
Psychosis	
Pyelonephritis	
Rabies	
Sacroiliac Joint Dysfunction	Y
Schizoaffective Disorder	
Schizophrenia	
Seizures	Y
Spondylitis, Ankylosing	Y
Tuberculosis	
Urinary Tract Infections	

This yielded 90 trials with 4533 participants. Of these, 58 trials had 300 or more participants. Of these, 56 trials were phase 2/3, phase 3 or phase 4.

We reviewed the title, drug, condition and comparator of each. We excluded one trial which compared two doses of the same drug, 1 trial which used an anti-psychotic to manage psychosis in Alzheimer's disease, and 11 trials which examined ineligible conditions (Table S1.5).

Table S1.5: YODA Excluded trials as ineligible conditions

Condition	Trials
Behavioural disturbance in dementia	3
Bipolar affective disorder	2
Bipolar affective disorder	1
Cancer	1
Critically ill	1
Metastatic breast cancer	1
Prostate cancer	2

On excluding these, 43 trials remained. Trial data was extracted from the clinicaltrials.gov register for each of these. None were excluded based on age of eligibility, numbers enrolled or the design of the study. Five were excluded on the basis of having very restrictive inclusion criteria meaning that these trials were tantamount to phase 2 trials.

The remaining 38 trials were requested from the YODA repository.

1.4 NIH Biolincc

We manually reviewed the titles and study summaries for 117 entries comprising trials (or in some cases groups of studies including one or more trial) listed as "randomised clinical trials" on the NIH BioLINCC repository. 93 were ineligible (Table S1.6).

Table S1.6: NIH BioLINCC "Randomised clinical trial" entries

Reason(s) for exclusion	Sum of Trial/network
Acute illness	3
Acute lung injury	10
Cancer	1
Children only	5
Depression	1
Infectious diseases	3
Maximum age of participants 55	1
Not a drug	36
Not a randomised controlled trial, non-drug	1
Too few participants	11
Too few adult participants (less than 300 adults)	1
Too few participants or not a trial	11
Too few participants, not a trial or not a drug intervention	1
Transplant	8
**Total**	**93**

Of the remaining 24 trials, 8 were conducted before 1990, 4 compared treatment strategies rather than specific interventions, one examined primary prevention in the general adult population and one included an ineligible treatment (female sex hormones). Ten trials remained.

1.5 Clinicaltrials.gov

1.5.1 Trial date, status, conditions and intervention types

We then searched clinicaltrials.gov on 2017-09-04 for eligible trials. The search was performed via the Clinical Trials Transformation Initiative (CTTI) Access to Aggregate Content of ClinicalTrials.gov (AACT) PostgreSQL database running the RPostgreSQL package from within R. As with the repository searches, the R files which give a complete description of the search, and can be used to reproduce both the search and data cleaning, will be made available via a code repository on publishing the findings. Details of the completeness of the database and its use for research are available at http://aact.ctti-clinicaltrials.org/.

Initial criteria were used to identify a set of trials from the clinicaltrials.gov (CTG) registry (Table S1.7).

Table S1.7: CTG Initial search criteria

Criteria	Trials
Start date >= 1990-01-01<br>Study type – interventional<br>Status – ‘Active, not recruiting’, ‘Completed’, ‘Terminated’<br>Phase – 2/3, 3 or 4<br>Enrollment >= 300 (or start_date < ‘2005-01-01’)	16957
Participants not excluded for being 60 or older	13079
Study design is “Factorial Assignment”, “Parallel Assignment” and “allocation random”	10841
Selected conditions (see below) found in study title or in browse_conditions or conditions table.	4348
Intervention type is “Biological” or “Drug”	3872

Conditions were eligible if the string (or the reverse of the string eg “Angina, Unstable” or “UnsTable angina”) corresponding to one or more the following MESH terms (or to a more specific terms in the MESH hierarchy) was found:- C05.116.198.579, C05.116.900.853.625.800, C05.550.114, C05.799.114, C05.799.414, C05.799.613, C06.405.117.119.500.204, C06.405.117.119.500.432, C06.405.117.119.500.450, C06.405.117.119.500.484, C06.405.117.119.500.484.500, C06.405.117.119.500.742, C06.405.117.620, C06.405.205.265.231, C06.405.205.731, C06.405.469.432, C06.405.608.348, C06.405.748.240, C06.405.748.398, C06.552.380.350.050, C08.127.108, C08.127.384, C08.127.446.567, C08.381.423, C08.381.483.487, C08.381.483.487.500, C08.381.495, C08.381.746, C08.381.765, C08.460.799, C08.674.095, C08.730.099.567, C09.603.799.315, C10.114.375.500, C10.228.140.079.862, C10.228.140.300.150, C10.228.140.300.275.800, C10.228.140.300.400, C10.228.140.300.510.200.325, C10.228.140.300.510.200.387, C10.228.140.300.510.200.418, C10.228.140.300.510.800.500, C10.228.140.300.775, C10.228.140.380.100, C10.228.140.380.230, C10.228.140.490, C10.228.140.546.399.750, C10.228.662.600, C10.574.812, C10.574.945.249, C10.803, C12.294.565.500, C12.777.419.192, C12.777.829.866, C12.777.934.284, C12.777.934.852, C13.351.968.829.813, C13.351.968.934.252, C13.351.968.934.814, C14.280.067.198, C14.280.067.248, C14.280.434, C14.280.647, C14.907.137.126.307, C14.907.137.126.307.500, C14.907.137.126.339, C14.907.137.126.372.500, C14.907.137.126.669, C14.907.253.092.477.200, C14.907.253.560.350.500, C14.907.253.855, C14.907.355, C14.907.355.350.700, C14.907.355.590, C14.907.355.830, C14.907.489, C14.907.585, C14.907.617, C17.300.480, C17.300.540, C17.300.775, C17.300.799, C17.800.784, C17.800.784.602, C17.800.784.801, C17.800.784.801.500, C17.800.859.675, C17.800.862.945, C18.452.394.750, C18.452.584.500.500.396, C18.452.584.500.500.438, C18.452.584.500.500.851, C18.452.648.398.450, C19.246.267, C19.246.300, C20.111.193, C20.111.197, C20.111.198, C20.111.199, C20.111.258.250.500, C20.111.327, C20.111.567, C20.543.480.680.095, C20.543.480.680.443, F03.087.400, or F03.675.700.

Table Table S1.8 groups these MeSH codes into wider categories and provides the preferred MeSH term.

Table S1.8: MeSH Terms Applied to clinicaltrials.gov database

Category	MeSH term	Code
Musculoskeletal	Osteoporosis	C05.116.198.579
	Spondyloarthopathies	C05.116.900.853 .625.800

Table S1.8: MeSH Terms Applied to clinicaltrials.gov database

Category	MeSH term	Code
	Arthritis	C05.550.114
	Arthritis, Rheumatoid	C05.799.114
	Gout	C05.799.414
	Osteoporosis	C05.799.613
Digestive system diseases	CREST Syndrome	C06.405.117.119.500.204
	Oesophageal Achalasia	C06.405.117.119.500.432
	Oesophageal spasm, diffuse	C06.405.117.119.500.450
	Gastro-oesophageal reflux	C06.405.117.119.500.484
	Laryngopharyngeal reflux	C06.405.117.119.500.484.500
	Plummer-Vinson Syndrome	C06.405.117.119.500.742
	Oesophagitis	C06.405.117.620
	Colitis, Ulcerative	C06.405.205.265.231
	Inflammatory Bowel Diseases	C06.405.205.731
	Inflammatory Bowel diseases	C06.405.469.432
	Oesophagitis, peptic	C06.405.608.348
	Duodenogastric reflux	C06.405.748.240
	Gastritis	C06.405.748.398
	Hepatitis, autoimmune	C06.552.380.350.050
Respiratory Tract Diseases	Asthma	C08.127.108
	Bronchiectasis	C08.127.384
	Bronchitis, chronic	C08.127.446.567
	Hypertension, Pulmonary	C08.381.423
	Idiopathic Interstitial Pneumonias	C08.381.483.487
	Idiopathic Pulmonary Fibrosis	C08.381.483.487.500
	Lung Diseases, Obstructive	C08.381.495
	Pulmonary Embolism	C08.381.746
	Pulmonary Fibrosis	C08.381.765
	Rhinitis	C08.460.799
	Asthma	C08.674.095
	Bronchitis, Chronic	C08.730.099.567
Otorhinolaryngologic Diseases	Rhinitis, Allergic	C09.603.799.315
	Multiple Sclerosis	C10.114.375.500
	Parkinsonian Disorders	C10.228.140.079.862
	Brain Ischaemia	C10.228.140.300.150
	Stroke, Lacunar	C10.228.140.300.275.800
	Dementia, Vascular	C10.228.140.300.400
	Infarction, Anterior Cerebral Artery	C10.228.140.300.510.200.325
	Infarction, Middle Cerebral Artery	C10.228.140.300.510.200.387
	Infarction, Posterior Cerebral Artery	C10.228.140.300.510.200.418
	Dementia, Vascular	C10.228.140.300.510.800.500
	Stroke	C10.228.140.300.775
	Alzheimer Disease	C10.228.140.380.100
	Dementia, Vascular	C10.228.140.380.230
	Epilepsy	C10.228.140.490
	Migraine Disorders	C10.228.140.546.399.750
	Parkinsonian Disorders	C10.228.662.600
	Parkinson Disease	C10.574.812
	Alzheimer Disease	C10.574.945.249
	Restless Leg Syndrome	C10.803
Male Urogenital Diseases	Prostatic Hyperplasia	C12.294.565.500
	Diabetic Nephropathies	C12.777.419.192
	Urinary Bladder, Overactive	C12.777.829.866

Table S1.8: MeSH Terms Applied to clinicaltrials.gov database

Category	MeSH term	Code
	Enuresis	C12.777.934.284
	Urinary Incontinence	C12.777.934.852
Female Urogenital Diseases	Urinary Bladder, Overactive	C13.351.968.829.813
	Enuresis	C13.351.968.934.252
	Urinary Incontinence	C13.351.968.934.814
Cardiovascular Diseases	Atrial Fibrillation	C14.280.067.198
	Atrial Flutter	C14.280.067.248
	Heart Failure	C14.280.434
	Myocardial Ischaemia	C14.280.647
	Atherosclerosis	C14.907.137.126.307
	Peripheral Arterial Disease	C14.907.137.126.307.500
	Coronary Artery Disease	C14.907.137.126.339
	Dementia, Vascular	C14.907.137.126.372.500
	Intermittent Claudication	C14.907.137.126.669
	Cerebral Infarction	C14.907.253.092.477.200
	Dementia, Vascular	C14.907.253.560.350.500
	Stroke	C14.907.253.855
	Embolism and Thrombosis	C14.907.355
	Pulmonary Embolism	C14.907.355.350.700
	Thromboembolism	C14.907.355.590
	Thrombosis	C14.907.355.830
	Hypertension	C14.907.489
	Myocardial Ischaemia	C14.907.585
	Peripheral Vascular Diseases	C14.907.617
Skin and Connective Tissue Diseases	Lupus Erythematosus, Systemic	C17.300.480
	Mixed Connective Tissue Disease	C17.300.540
	Rheumatic Diseases	C17.300.775
	Scleroderma, Systemic	C17.300.799
	Scleroderma, Systemic	C17.800.784
	Scleroderma, Diffuse	C17.800.784.602
	Scleroderma, Limited	C17.800.784.801
	CREST Syndrome	C17.800.784.801.500
	Psoriasis	C17.800.859.675
	Urticaria	C17.800.862.945
Nutritional and Metabolic Diseases	Diabetes Mellitus	C18.452.394.750
	Hypercholisterolaemia	C18.452.584.500.500.396
	Hyperlipidaemia, Familial Combined	C18.452.584.500.500.438
	Hypertriglyceridiaemia	C18.452.584.500.500.851
	Hyperlipidaemia, Familial Combined	C18.452.648.398.450
Endocrine System Diseases	Diabetes Mellitus, Type 1	C19.246.267
	Diabetes Mellitus, Type 2	C19.246.300
Immune System Diseases	Anti-Neutrophil Cytoplasmic Antibody-Associated Vasculitis	C20.111.193
	Antiphospholipid Syndrome	C20.111.197
	Arthritis, Juvenile	C20.111.198
	Arthritis, Rheumatoid	C20.111.199
	Multiple Sclerosis	C20.111.258.250.500
	Diabetes Mellitus, Type 1	C20.111.327
	Hepatitis, Autoimmune	C20.111.567

Table S1.8: MeSH Terms Applied to clinicaltrials.gov database

Category	MeSH term	Code
	Asthma	C20.543.480.680.095
	Rhinitis, Allergic	C20.543.480.680.443

Six repository trials were not found in the CTG register (Table S1.9). Of these, the trials which were ineligible were excluded from the repository set. Those with clerical errors only were retained. This resulted in 197 eligible trials in the CSDR repository, 9 trials in the NIH repository, and 38 trials in the YODA repository.

Table S1.9: Excluded repository trials following initial CTG search

Trial ID		Reason	Type of reason
BioLINCC	NCT00786474	No selected MESH condition listed	Ineligible
CSDR	NCT00240435	Listed as device comparison (tiotropium versus tiotropium)	Ineligible
CSDR	NCT00239473	Listed as device comparison (tiotropium versus tiotropium)	Ineligible
CSDR	NCT00348309	Phase not recorded on CTG (was Phase 3)	Clerical error
YODA	NCT00096655	Erroneously states 'accepts healthy volunteers'	Clerical error
YODA	NCT00236028	Missing primary completion date	Clerical error

1.5.2 Specific interventions

Eligible drugs were those which belonged to a defined set of relevant WHO ATC classes and which were not-topical, and had not been withdrawn or discontinued.

To identify drugs from text strings, we matched the strings for interventions to the RxNorm metathesauras and Pubchem. For strings where there was no match, we used manual review to assign a name. We then assigned each of the normalized drug names to a WHO ATC class. Drugs were assigned to one or more 7-character ATC-class uniquely identifying each drug. Where a drug did not yet have any ATC-code, the drug was assigned to the closest 5-character ATC class, and a 2-character alphanumeric code was appended to make a new 7-character code. Where interventions were defined in the trial only as a drug-class (eg a trial of ACE-inhibitor therapy) a WHO ATC code for the most specific class was assigned.

Any drug with a 7-character code starting with any of the following 3, 4, and 5-character ATC codes was defined as relevant:- A02A, A02AC, A02BA, A02BC, A02BX, A10AB, A10AC, A10AD, A10AE, A10BA, A10BB, A10BF, A10BG, A10BH, A10BJ, A10BK, A10BX, B01AA, B01AB, B01AC, B01AD, B01AE, B01AF, B01AX, C01AA, C01BA, C01BB, C01BC, C01BD, C01BG, C01CA, C01CE, C01CX, C01DA, C01DX, C01EA, C01EB, C02AA, C02AB, C02AC, C02CA, C02DB, C02DD, C02KX, C03, C03AA, C03BA, C03BD, C03CA, C03DA, C03DB, C03XA, C04AC, C04AD, C05AA, C05AB, C05AD, C05AE, C05BA, C07AA, C07AB, C07AG, C08, C08CA, C08DA, C08DB, C09AA, C09CA, C09DB, C09XA, C10AA, C10AB, C10AC, C10AD, C10AX, G04BD, G04BE, G04CA, G04CB, H05AA, H05BX, L01AA, L01BA, L01BB, L01BC, L01CD, L01XC, L01XE, L01XX, L04AA, L04AB, L04AC, L04AD, L04AX, M01AB, M01AC, M01AE, M01AH, M01AX, M04AA, M04AB, M04AC, M05BA, M05BX, N03AA, N03AB, N03AF, N03AG, N03AX, N04AA, N04BA, N04BC, N04BD, N04BX, N06DA, N06DX, R03AA, R03AC, R03BA, R03BB, R03CA, R03CC, R03DA, R03DC, R03DX. Secondly, any intervention assigned to a drug-class with a code exactly matching the following codes was defined as relevant:- A02A, A02B, A10A, A10B, A10BA, A10BB, A10BF, A10BG, A10BH, A10BJ, A10BK, A10BX, B01A, B01AB, B01AD, C01B, C02, C03A, C03AA, C03CA, C07A, C08, C09A, C09AA, C09CA, C10A, C10AA, L04AB, M01A, M01AE, N03A, N04BD, R03AC, R03BA, R03BB, R06A.

Using the normalized names, we used a combination of string comparison functions and manual review to restrict the set of trials to those where one or more arm-comparison compared eligible drugs (or classes), or compared an eligible drug to either placebo, usual-care or a "standard comparator".

Especially for later records, clinicaltrials.gov provided information on the study arm for each intervention. However, for most earlier records and some later records the arm in which each drug was used was not specified. As such, these two types of record were examined separately, resulting in a number of trials being excluded (Table S1.10).

Table S1.10: Trial exclusions on basis of drug comparisons

	No arm-specific information	Arm specific information	Total
All	973	2902	3875
Exclude trials with same-drug comparisons	973	2469	3442
Exclude topical	947	2361	3308
Exclude discontinued	888	2310	3198
Exclude not selected	721	1949	2670

These exclusions included 22 repository trial exclusions (Table S1.11). 18 CSDR, 2 YODA and 2 BioLINCC trials were excluded. Three of these trials were wrongly excluded (first two rows in Table 10) and so have been retained in the set of trials (Biolincc- NCT00000560, CSDR - NCT00428090 and YODA- NCT00973479).

Table S1.11: Repository trials excluded on basis of drug comparisons

Reason Excluded	Trials
Mislabelled name	1
Wrongly identified as same drug comparison – included open label extension	2
Discontinued	1
No relevant drug compared based on WHO ATC code	1
Same drug all arms	11
Topical	5
Withdrawal study	1

1.5.3 Enrollment

Of the 2670 trials the following was found regarding enrollment. Note that for more recently registered trials, those with fewer than 300 participants have already been excluded. Enrollment data was not recorded in a relevant CTG controlled field for 21 trials, and could not be located at all (in any of free text fields in clinicaltrials.gov, listed related publications, or on searching pubmed and google for the clinical trial ID) for an additional 17 trials. In total, 418 trials included fewer than 300 participants. None of the repository trials had missing enrollment data or fewer than 300 participants.

1.6 Final set of denominator trials

This left a final “denominator” dataset of 2235 clinical trials (Table: 1.12), and a final repository dataset of 225 trials. The following Table provides a summary of the number of trials at each stage

Table S1.12: Final denominator set of trials

	CSDR	YODA	NIH	Total repository	Total registry
Initial set of trials	199	38	10	247	
Start date, phase, enrolment, age, design, conditions, broad indication type	197	38	9	244	3872
Ineligible drugs	180	37	8	225	2670
Less than 300 participants	180	37	8	225	2235

1.7 Final set of numerator trials

Of the IPD trials listed above, 96 had been provided by CSDR, 37 by YODA and 8 by NIH at the time of this analysis. All of the YODA trials, none of the NIH trials and 87 of the CSDR trials included data on comorbid diseases which allowed us to conduct these analyses. Of the 9 CSDR trials which did not have data on comorbidity, 5 trials from a single sponsor redacted all concomitant medication data and the remaining 4 trials (where the indication was myocardial infarction) only recorded cardiometabolic drugs. This left a total of 124 trials.

Three of these trials which were for Chronic Idiopathic Urticaria (NCT01264939, NCT01287117 and NCT01292473) which was a condition we could not detect in the primary care data. One trial was examining the use of anticoagulant medication to prevent thromboebolic disease for “solid tumour” (NCT00694382). We excluded this as it was not clear how it should be identified using READ codes. One further trial was excluded as the indication was chronic allergic rhinitis (NCT00694382), which we felt would be difficult to distinguish from seasonal allergic rhinitis in the primary care data. This left 119 trials.

A further 3 trials (2 trials for thromboprophylaxis post arthroplasty of the knee and 1 post arthroplasty of the hip) were excluded from the final comparison. This was because, for a comorbidity count, prescribing patterns in the pre- or post- operative period may not accurately reflect long-term management of comorbidities. This left a total of 116 trials for the final comparison.

Next
