# Supplementary material for: Representation of people with comorbidity and multimorbidity in clinical trials of novel drug therapies: an individual-level participant data analysis
Source: BMC Med. 2019 Nov 12;17:201. doi: 10.1186/s12916-019-1427-1 (PMC6849229; doi:10.1186/s12916-019-1427-1)
Supplement: Supplementary file 2 — Additional file 2. Representativeness-of-community-data-sail.pdf: Analysis of the representativeness of the community sample. [file 12916_2019_1427_MOESM2_ESM.pdf]

2 Representativeness of community data (SAIL)

The following tables (Tables 2.1, 2.2 and 2.3) examine the representativeness of SAIL by comparing age, sex and socio-economic status between SAIL and non-SAIL primary care practices.

2.1 Sex

Table S2.1: Proportion of men and women in all Wales primary care practices, and SAIL primary care practices

Sex	All practices	SAIL practices	Non-SAIL practices
Male	1640340 (49.9%)	1300625 (49.9%)	339715 (50.0%)
Female	1646863 (50.1%)	1307793 (50.1%)	339070 (50.0%)

2.2 Age

Table S2.2: Proportion of different ages in all Wales primary care practices, and SAIL primary care practices

Age (years)	All practices	SAIL practices	Non-SAIL practices
0 to 17	662165 (20.1%)	527491 (20.2%)	134674 (19.8%)
18 to 64	1981499 (60.3%)	1579351 (60.5%)	402148 (59.2%)
65 plus	643544 (19.6%)	501579 (19.2%)	141965 (20.9%)

2.3 Socio-economic status

Table S2.3: Proportion of people of different ages in all Wales primary care practices, and SAIL primary care practices

Socio-economic deprivation (quintiles)	All practices	SAIL practices	Non-SAIL practices
Q1	627368 (19.1%)	529430 (20.3%)	97938 (14.4%)
Q2	613593 (18.7%)	483424 (18.5%)	130169 (19.2%)
Q3	612592 (18.6%)	489567 (18.8%)	123025 (18.1%)
Q4	582833 (17.7%)	422413 (16.2%)	160420 (23.6%)
Q5	595508 (18.1%)	485554 (18.6%)	109954 (16.2%)
Missing	255314 (7.8%)	198033 (7.6%)	57281 (8.4%)
