# Supplementary material for: Representation of people with comorbidity and multimorbidity in clinical trials of novel drug therapies: an individual-level participant data analysis
Source: BMC Med. 2019 Nov 12;17:201. doi: 10.1186/s12916-019-1427-1 (PMC6849229; doi:10.1186/s12916-019-1427-1)
Supplement: Supplementary file 3 — Additional file 3. Selection-of-patients-and-participants-from-primary-care-data-read-codes.pdf: Read codes used to identify index conditions. [file 12916_2019_1427_MOESM3_ESM.pdf]

3 Selection of patients and participants from Primary Care data - READ codes

3.1 Primary care

For primary care data, READ codes were used to identify conditions matching the trial indications. READ codes are shown in Table S3.1. There are 50 rows in the cars dataframe.

Table S3.1: Primary care READ codes used to identify patients with trial indications

Condition	Read.code	Description	condition_rename
Dementia (Alzheimer's)	Eu00.	[X]Dementia in Alzheimer's disease	Alzheimer's Disease
Dementia (Alzheimer's)	Eu000	[X]Dementia in Alzheimer's disease with early onset	Alzheimer's Disease
Dementia (Alzheimer's)	Eu001	[X]Dementia in Alzheimer's disease with late onset	Alzheimer's Disease
Dementia (Alzheimer's)	Eu002	[X]Dementia in Alzheimer's dis, atypical or mixed type	Alzheimer's Disease
Dementia (Alzheimer's)	Eu00z	[X]Dementia in Alzheimer's disease, unspecified	Alzheimer's Disease
Dementia (Alzheimer's)	F110.	Alzheimer's disease	Alzheimer's Disease
Dementia (Alzheimer's)	F1100	Alzheimer's disease with early onset	Alzheimer's Disease
Dementia (Alzheimer's)	F1101	Alzheimer's disease with late onset	Alzheimer's Disease
Dementia (Alzheimer's)	Fyu30	[X]Other Alzheimer's disease	Alzheimer's Disease
Asthma	173A.	Exercise induced asthma	Asthma
Asthma	173c.	Occupational asthma	Asthma
Asthma	173d.	Work aggravated asthma	Asthma
Asthma	1780.	Aspirin induced asthma	Asthma
Asthma	1O2..	Asthma confirmed	Asthma
Asthma	663d.	Emergency asthma admission since last appointment	Asthma
Asthma	663e.	Asthma restricts exercise	Asthma
Asthma	663e0	Asthma sometimes restricts exercise	Asthma
Asthma	663e1	Asthma severely restricts exercise	Asthma
Asthma	663f.	Asthma never restricts exercise	Asthma
Asthma	663h.	Asthma - currently dormant	Asthma
Asthma	663j.	Asthma - currently active	Asthma
Asthma	663m.	Asthma accident and emergency attendance since last visit	Asthma
Asthma	663n.	Asthma treatment compliance satisfactory	Asthma
Asthma	663N.	Asthma disturbing sleep	Asthma
Asthma	663N0	Asthma causing night waking	Asthma
Asthma	663N1	Asthma disturbs sleep weekly	Asthma
Asthma	663N2	Asthma disturbs sleep frequently	Asthma
Asthma	663O.	Asthma not disturbing sleep	Asthma

Table S3.1: Primary care READ codes used to identify patients with trial indications

Condition	Read.code	Description	condition_rename
Asthma	663O0	Asthma never disturbs sleep	Asthma
Asthma	663p.	Asthma treatment compliance unsatisfactory	Asthma
Asthma	663P.	Asthma limiting activities	Asthma
Asthma	663q.	Asthma daytime symptoms	Asthma
Asthma	663Q.	Asthma not limiting activities	Asthma
Asthma	663r.	Asthma causes night symptoms 1 to 2 times per month	Asthma
Asthma	663s.	Asthma never causes daytime symptoms	Asthma
Asthma	663t.	Asthma causes daytime symptoms 1 to 2 times per month	Asthma
Asthma	663u.	Asthma causes daytime symptoms 1 to 2 times per week	Asthma
Asthma	663U.	Asthma management plan given	Asthma
Asthma	663v.	Asthma causes daytime symptoms most days	Asthma
Asthma	663V.	Asthma severity	Asthma
Asthma	663P.	Asthma limiting activities	Asthma
Asthma	663q.	Asthma daytime symptoms	Asthma
Asthma	663r.	Asthma causes night symptoms 1 to 2 times per month	Asthma
Asthma	663u.	Asthma causes daytime symptoms 1 to 2 times per week	Asthma
Asthma	663v.	Asthma causes daytime symptoms most days	Asthma
Asthma	663V0	Occasional asthma	Asthma
Asthma	663V1	Mild asthma	Asthma
Asthma	663V2	Moderate asthma	Asthma
Asthma	663V3	Severe asthma	Asthma
Asthma	663w.	Asthma limits walking up hills or stairs	Asthma
Asthma	663W.	Asthma prophylactic medication used	Asthma
Asthma	663x.	Asthma limits walking on the flat	Asthma
Asthma	663y.	Number of asthma exacerbations in past year	Asthma
Asthma	66Y5.	Change in asthma management plan	Asthma
Asthma	66Y9.	Step up change in asthma management plan	Asthma
Asthma	66YA.	Step down change in asthma management plan	Asthma
Asthma	66YC.	Absent from work or school due to asthma	Asthma
Asthma	66YE.	Asthma monitoring due	Asthma
Asthma	66YJ.	Asthma annual review	Asthma
Asthma	66YK.	Asthma follow-up	Asthma
Asthma	66YP.	Asthma night-time symptoms	Asthma
Asthma	66YQ.	Asthma monitoring by nurse	Asthma
Asthma	66YR.	Asthma monitoring by doctor	Asthma
Asthma	8793.	Asthma control step 0	Asthma
Asthma	8794.	Asthma control step 1	Asthma
Asthma	8795.	Asthma control step 2	Asthma
Asthma	8796.	Asthma control step 3	Asthma
Asthma	8797.	Asthma control step 4	Asthma

Table S3.1: Primary care READ codes used to identify patients with trial indications

Condition	Read.code	Description	condition_rename
Asthma	8798.	Asthma control step 5	Asthma
Asthma	8B3j.	Asthma medication review	Asthma
Asthma	8CR0.	Asthma clinical management plan	Asthma
Asthma	8791.	Further asthma - drug prevent.	Asthma
Asthma	9hA..	Exception reporting: asthma quality indicators	Asthma
Asthma	H3120	Chronic asthmatic bronchitis	Asthma
Asthma	H33..	Asthma	Asthma
Asthma	H330.	Extrinsic (atopic) asthma	Asthma
Asthma	H3300	Extrinsic asthma without status asthmaticus	Asthma
Asthma	H3301	Extrinsic asthma with status asthmaticus	Asthma
Asthma	H330z	Extrinsic asthma NOS	Asthma
Asthma	H331.	Intrinsic asthma	Asthma
Asthma	H3310	Intrinsic asthma without status asthmaticus	Asthma
Asthma	H3311	Intrinsic asthma with status asthmaticus	Asthma
Asthma	H331z	Intrinsic asthma NOS	Asthma
Asthma	H332.	Mixed asthma	Asthma
Asthma	H333.	Acute exacerbation of asthma	Asthma
Asthma	H334.	Brittle asthma	Asthma
Asthma	H33z.	Asthma unspecified	Asthma
Asthma	H33z0	Status asthmaticus NOS	Asthma
Asthma	H33z1	Asthma attack	Asthma
Asthma	H33z2	Late-onset asthma	Asthma
Asthma	H33zz	Asthma NOS	Asthma
Asthma	H35y6	Sequoiosis (red-cedar asthma)	Asthma
Asthma	H35y7	Wood asthma	Asthma
Asthma	H47y0	Detergent asthma	Asthma
Atrial fibrillation	14AN.	H/O: atrial fibrillation	Atrial Fibrillation
Atrial fibrillation	3272.	ECG: atrial fibrillation	Atrial Fibrillation
Atrial fibrillation	662S.	Atrial fibrillation monitoring	Atrial Fibrillation
Atrial fibrillation	6A9..	Atrial fibrillation annual review	Atrial Fibrillation
Atrial fibrillation	G573.	Atrial fibrillation and flutter	Atrial Fibrillation
Atrial fibrillation	G5730	Atrial fibrillation	Atrial Fibrillation
Atrial fibrillation	G5732	Paroxysmal atrial fibrillation	Atrial Fibrillation
Atrial fibrillation	G5733	Non-rheumatic atrial fibrillation	Atrial Fibrillation
Atrial fibrillation	G5734	Permanent atrial fibrillation	Atrial Fibrillation
Atrial fibrillation	G5735	Persistent atrial fibrillation	Atrial Fibrillation
Atrial fibrillation	G573z	Atrial fibrillation and flutter NOS	Atrial Fibrillation
Axial spondyloarthritis	XabRZ	Axial spondyloarthritis	Axial Spondyloarthritis
Axial spondyloarthritis	N100.	Ankylosing spondylitis	Axial Spondyloarthritis
Axial spondyloarthritis	X7023	Ankylosing spondylitis with organ / system involvement	Axial Spondyloarthritis
Axial spondyloarthritis	X7024	Ankylosing spondylitis with multisystem involvement	Axial Spondyloarthritis
Axial spondyloarthritis	X701x	Juvenile ankylosing spondylitis	Axial Spondyloarthritis
Benign prostatic hyperplasia	K20..	Benign prostatic hypertrophy	Benign Prostatic Hyperplasia
Benign prostatic hyperplasia	K200.	Prostatic hyperplasia unspecified	Benign Prostatic Hyperplasia

Table S3.1: Primary care READ codes used to identify patients with trial indications

Condition	Read.code	Description	condition_rename
Benign prostatic hyperplasia	K201.	Prostatic hyperplasia of the lateral lobe	Benign Prostatic Hyperplasia
Benign prostatic hyperplasia	K202.	Prostatic hyperplasia of the medial lobe	Benign Prostatic Hyperplasia
Benign prostatic hyperplasia	K20z.	Prostatic hyperplasia NOS	Benign Prostatic Hyperplasia
Diabetes Mellitus, Type 2	66A3.	Diabetic on diet only	Diabetes Mellitus, Type 2
Diabetes Mellitus, Type 2	66A4.	Diabetic on oral treatment	Diabetes Mellitus, Type 2
Diabetes Mellitus, Type 2	66Ao.	Diabetes type 2 review	Diabetes Mellitus, Type 2
Diabetes Mellitus, Type 2	C1074	NIDDM with peripheral circulatory disorder	Diabetes Mellitus, Type 2
Diabetes Mellitus, Type 2	C109.	Non-insulin dependent diabetes mellitus	Diabetes Mellitus, Type 2
Diabetes Mellitus, Type 2	C1090	Non-insulin-dependent diabetes mellitus with renal comps	Diabetes Mellitus, Type 2
Diabetes Mellitus, Type 2	C1091	Non-insulin-dependent diabetes mellitus with ophthalm comps	Diabetes Mellitus, Type 2
Diabetes Mellitus, Type 2	C1092	Non-insulin-dependent diabetes mellitus with neuro comps	Diabetes Mellitus, Type 2
Diabetes Mellitus, Type 2	C1093	Non-insulin-dependent diabetes mellitus with multiple comps	Diabetes Mellitus, Type 2
Diabetes Mellitus, Type 2	C1094	Non-insulin dependent diabetes mellitus with ulcer	Diabetes Mellitus, Type 2
Diabetes Mellitus, Type 2	C1095	Non-insulin dependent diabetes mellitus with gangrene	Diabetes Mellitus, Type 2
Diabetes Mellitus, Type 2	C1096	Non-insulin-dependent diabetes mellitus with retinopathy	Diabetes Mellitus, Type 2
Diabetes Mellitus, Type 2	C1097	Non-insulin dependent diabetes mellitus - poor control	Diabetes Mellitus, Type 2
Diabetes Mellitus, Type 2	C1099	Non-insulin-dependent diabetes mellitus without complication	Diabetes Mellitus, Type 2
Diabetes Mellitus, Type 2	C109A	Non-insulin dependent diabetes mellitus with mononeuropathy	Diabetes Mellitus, Type 2
Diabetes Mellitus, Type 2	C109B	Non-insulin dependent diabetes mellitus with polyneuropathy	Diabetes Mellitus, Type 2
Diabetes Mellitus, Type 2	C109C	Non-insulin dependent diabetes mellitus with nephropathy	Diabetes Mellitus, Type 2
Diabetes Mellitus, Type 2	C109D	Non-insulin dependent diabetes mellitus with hypoglyca coma	Diabetes Mellitus, Type 2
Diabetes Mellitus, Type 2	C109E	Non-insulin depend diabetes mellitus with diabetic cataract	Diabetes Mellitus, Type 2
Diabetes Mellitus, Type 2	C109F	Non-insulin-dependent d m with peripheral angiopath	Diabetes Mellitus, Type 2
Diabetes Mellitus, Type 2	C109G	Non-insulin dependent diabetes mellitus with arthropathy	Diabetes Mellitus, Type 2
Diabetes Mellitus, Type 2	C109H	Non-insulin dependent d m with neuropathic arthropathy	Diabetes Mellitus, Type 2
Diabetes Mellitus, Type 2	C109J	Insulin treated Type 2 diabetes mellitus	Diabetes Mellitus, Type 2
Diabetes Mellitus,	C109K	Hyperosmolar non-ketotic state in type	Diabetes Mellitus, Type

Table S3.1: Primary care READ codes used to identify patients with trial indications

Condition	Read.code	Description	condition_rename
Type 2		2 diabetes mellitus	2
Diabetes Mellitus, Type 2	C10F.	Type 2 diabetes mellitus	Diabetes Mellitus, Type 2
Diabetes Mellitus, Type 2	C10F0	Type 2 diabetes mellitus with renal complications	Diabetes Mellitus, Type 2
Diabetes Mellitus, Type 2	C10F1	Type 2 diabetes mellitus with ophthalmic complications	Diabetes Mellitus, Type 2
Diabetes Mellitus, Type 2	C10F2	Type 2 diabetes mellitus with neurological complications	Diabetes Mellitus, Type 2
Diabetes Mellitus, Type 2	C10F3	Type 2 diabetes mellitus with multiple complications	Diabetes Mellitus, Type 2
Diabetes Mellitus, Type 2	C10F4	Type 2 diabetes mellitus with ulcer	Diabetes Mellitus, Type 2
Diabetes Mellitus, Type 2	C10F5	Type 2 diabetes mellitus with gangrene	Diabetes Mellitus, Type 2
Diabetes Mellitus, Type 2	C10F6	Type 2 diabetes mellitus with retinopathy	Diabetes Mellitus, Type 2
Diabetes Mellitus, Type 2	C10F7	Type 2 diabetes mellitus - poor control	Diabetes Mellitus, Type 2
Diabetes Mellitus, Type 2	C10F9	Type 2 diabetes mellitus without complication	Diabetes Mellitus, Type 2
Diabetes Mellitus, Type 2	C10FA	Type 2 diabetes mellitus with mononeuropathy	Diabetes Mellitus, Type 2
Diabetes Mellitus, Type 2	C10FB	Type 2 diabetes mellitus with polyneuropathy	Diabetes Mellitus, Type 2
Diabetes Mellitus, Type 2	C10FC	Type 2 diabetes mellitus with nephropathy	Diabetes Mellitus, Type 2
Diabetes Mellitus, Type 2	C10FD	Type 2 diabetes mellitus with hypoglycaemic coma	Diabetes Mellitus, Type 2
Diabetes Mellitus, Type 2	C10FE	Type 2 diabetes mellitus with diabetic cataract	Diabetes Mellitus, Type 2
Diabetes Mellitus, Type 2	C10FF	Type 2 diabetes mellitus with peripheral angiopathy	Diabetes Mellitus, Type 2
Diabetes Mellitus, Type 2	C10FG	Type 2 diabetes mellitus with arthropathy	Diabetes Mellitus, Type 2
Diabetes Mellitus, Type 2	C10FH	Type 2 diabetes mellitus with neuropathic arthropathy	Diabetes Mellitus, Type 2
Diabetes Mellitus, Type 2	C10FJ	Insulin treated Type 2 diabetes mellitus	Diabetes Mellitus, Type 2
Diabetes Mellitus, Type 2	C10FK	Hyperosmolar non-ketotic state in type 2 diabetes mellitus	Diabetes Mellitus, Type 2
Diabetes Mellitus, Type 2	C10FL	Type 2 diabetes mellitus with persistent proteinuria	Diabetes Mellitus, Type 2
Diabetes Mellitus, Type 2	C10FM	Type 2 diabetes mellitus with persistent microalbuminuria	Diabetes Mellitus, Type 2
Diabetes Mellitus, Type 2	C10FN	Type 2 diabetes mellitus with ketoacidosis	Diabetes Mellitus, Type 2
Diabetes Mellitus, Type 2	C10FP	Type 2 diabetes mellitus with ketoacidotic coma	Diabetes Mellitus, Type 2
Diabetes Mellitus, Type 2	C10FQ	Type 2 diabetes mellitus with exudative maculopathy	Diabetes Mellitus, Type 2
Diabetes Mellitus, Type 2	C10FR	Type 2 diabetes mellitus with gastroparesis	Diabetes Mellitus, Type 2

Table S3.1: Primary care READ codes used to identify patients with trial indications

Condition	Read.code	Description	condition_rename
Erectile dysfunction	E2273	Erectile dysfunction	Erectile dysfunction
Erectile dysfunction	Xaa8r	Erectile dysfunction due to diabetes mellitus	Erectile dysfunction
Erectile dysfunction	X400F	Drug-induced impotence	Erectile dysfunction
Erectile dysfunction	X400G	Endocrine impotence	Erectile dysfunction
Erectile dysfunction	X400H	Neuropathic impotence	Erectile dysfunction
Erectile dysfunction	Xa24H	Arteriopathic impotence	Erectile dysfunction
Erectile dysfunction	X400I	Psychogenic impotence	Erectile dysfunction
Erectile dysfunction	K27y1	Impotence of organic origin	Erectile dysfunction
Erectile dysfunction	XaXgw	C/O erectile dysfunction	Erectile dysfunction
Erectile dysfunction	7A6G5	Ligation of penile veins for impotence	Erectile dysfunction
Erectile dysfunction	X74UL	Provision of device for impotence	Erectile dysfunction
Erectile dysfunction	X74UM	Provision of penile vacuum constriction device	Erectile dysfunction
Erectile dysfunction	X74UN	Provision of penile tension band device	Erectile dysfunction
Erectile dysfunction	X77IU	Physiological tests for impotence	Erectile dysfunction
Erectile dysfunction	X77IV	Nocturnal penile tumescence	Erectile dysfunction
Erectile dysfunction	X77IW	Corporeal veno-occlusive function	Erectile dysfunction
Erectile dysfunction	X77IX	Intracavernous injection test using vaso-active agent	Erectile dysfunction
Erectile dysfunction	X71FJ	Advice on technique for impotence	Erectile dysfunction
Erectile dysfunction	X30HR	Penile revascularisation for impotence	Erectile dysfunction
Hypertension	662G.	Hypertensive treatm.changed	Hypertension
Hypertension	662O.	On treatment for hypertension	Hypertension
Hypertension	662P.	Hypertension monitoring	Hypertension
Hypertension	662q.	Trial reduction of antihypertensive therapy	Hypertension
Hypertension	662r.	Trial withdrawal of antihypertensive therapy	Hypertension
Hypertension	8B26.	Antihypertensive therapy	Hypertension
Hypertension	8BL0.	Patient on maximal tolerated antihypertensive therapy	Hypertension
Hypertension	8CR4.	Hypertension clinical management plan	Hypertension
Hypertension	F4042	Blind hypertensive eye	Hypertension
Hypertension	F4213	Hypertensive retinopathy	Hypertension
Hypertension	F4504	Ocular hypertension	Hypertension
Hypertension	G2…	Hypertensive disease	Hypertension
Hypertension	G20..	Essential hypertension	Hypertension
Hypertension	G200.	Malignant essential hypertension	Hypertension
Hypertension	G201.	Benign essential hypertension	Hypertension
Hypertension	G202.	Systolic hypertension	Hypertension
Hypertension	G203.	Diastolic hypertension	Hypertension
Hypertension	G20z.	Essential hypertension NOS	Hypertension
Hypertension	G21..	Hypertensive heart disease	Hypertension
Hypertension	G210.	Malignant hypertensive heart disease	Hypertension
Hypertension	G2100	Malignant hypertensive heart disease without CCF	Hypertension
Hypertension	G2101	Malignant hypertensive heart disease with CCF	Hypertension
Hypertension	G210z	Malignant hypertensive heart disease NOS	Hypertension

Table S3.1: Primary care READ codes used to identify patients with trial indications

Condition	Read.code	Description	condition_rename
Hypertension	G211.	Benign hypertensive heart disease	Hypertension
Hypertension	G2110	Benign hypertensive heart disease without CCF	Hypertension
Hypertension	G2111	Benign hypertensive heart disease with CCF	Hypertension
Hypertension	G211z	Benign hypertensive heart disease NOS	Hypertension
Hypertension	G21z.	Hypertensive heart disease NOS	Hypertension
Hypertension	G21z0	Hypertensive heart disease NOS without CCF	Hypertension
Hypertension	G21z1	Hypertensive heart disease NOS with CCF	Hypertension
Hypertension	G21zz	Hypertensive heart disease NOS	Hypertension
Hypertension	G22..	Hypertensive renal disease	Hypertension
Hypertension	G220.	Malignant hypertensive renal disease	Hypertension
Hypertension	G221.	Benign hypertensive renal disease	Hypertension
Hypertension	G222.	Hypertensive renal disease with renal failure	Hypertension
Hypertension	G22z.	Hypertensive renal disease NOS	Hypertension
Hypertension	G23..	Hypertensive heart and renal disease	Hypertension
Hypertension	G230.	Malignant hypertensive heart and renal disease	Hypertension
Hypertension	G231.	Benign hypertensive heart and renal disease	Hypertension
Hypertension	G232.	Hypertensive heart&renal dis wth (congestive) heart failure	Hypertension
Hypertension	G233.	Hypertensive heart and renal disease with renal failure	Hypertension
Hypertension	G234.	Hyperten heart&renal dis+both(congestv)heart and renal fail	Hypertension
Hypertension	G23z.	Hypertensive heart and renal disease NOS	Hypertension
Hypertension	G24..	Secondary hypertension	Hypertension
Hypertension	G240.	Secondary malignant hypertension	Hypertension
Hypertension	G2400	Secondary malignant renovascular hypertension	Hypertension
Hypertension	G240z	Secondary malignant hypertension NOS	Hypertension
Hypertension	G241.	Secondary benign hypertension	Hypertension
Hypertension	G2410	Secondary benign renovascular hypertension	Hypertension
Hypertension	G241z	Secondary benign hypertension NOS	Hypertension
Hypertension	G244.	Hypertension secondary to endocrine disorders	Hypertension
Hypertension	G24z.	Secondary hypertension NOS	Hypertension
Hypertension	G24z0	Secondary renovascular hypertension NOS	Hypertension
Hypertension	G24z1	Hypertension secondary to drug	Hypertension
Hypertension	G24zz	Secondary hypertension NOS	Hypertension
Hypertension	G2y..	Other specified hypertensive disease	Hypertension
Hypertension	G2z..	Hypertensive disease NOS	Hypertension
Hypertension	G672.	Hypertensive encephalopathy	Hypertension
Hypertension	Gyu2.	[X]Hypertensive diseases	Hypertension

Table S3.1: Primary care READ codes used to identify patients with trial indications

Condition	Read.code	Description	condition_rename
Hypertension	Gyu20	[X]Other secondary hypertension	Hypertension
Hypertension	Gyu21	[X]Hypertension secondary to other renal disorders	Hypertension
Pulmonary hypertension	G410.	Primary pulmonary hypertension	Hypertension, Pulmonary
Pulmonary hypertension	X2036	Sporadic primary pulmonary hypertension	Hypertension, Pulmonary
Pulmonary hypertension	X2037	Familial primary pulmonary hypertension	Hypertension, Pulmonary
Inflammatory Bowel disease	14C4.	H/O: colitis	Inflammatory bowel disease
Inflammatory Bowel disease	J08z9	Orofacial Crohn’s disease	Inflammatory bowel disease
Inflammatory Bowel disease	J40..	Regional enteritis - Crohn’s disease	Inflammatory bowel disease
Inflammatory Bowel disease	J400.	Regional enteritis of the small bowel	Inflammatory bowel disease
Inflammatory Bowel disease	J4000	Regional enteritis of the duodenum	Inflammatory bowel disease
Inflammatory Bowel disease	J4001	Regional enteritis of the jejunum	Inflammatory bowel disease
Inflammatory Bowel disease	J4002	Crohn’s disease of the terminal ileum	Inflammatory bowel disease
Inflammatory Bowel disease	J4003	Crohn’s disease of the ileum unspecified	Inflammatory bowel disease
Inflammatory Bowel disease	J4004	Crohn’s disease of the ileum NOS	Inflammatory bowel disease
Inflammatory Bowel disease	J4005	Exacerbation of Crohn’s disease of small intestine	Inflammatory bowel disease
Inflammatory Bowel disease	J400z	Crohn’s disease of the small bowel NOS	Inflammatory bowel disease
Inflammatory Bowel disease	J401.	Regional enteritis of the large bowel	Inflammatory bowel disease
Inflammatory Bowel disease	J4010	Regional enteritis of the colon	Inflammatory bowel disease
Inflammatory Bowel disease	J4011	Regional enteritis of the rectum	Inflammatory bowel disease
Inflammatory Bowel disease	J4012	Exacerbation of Crohn’s disease of large intestine	Inflammatory bowel disease
Inflammatory Bowel disease	J401z	Crohn’s disease of the large bowel NOS	Inflammatory bowel disease
Inflammatory Bowel disease	J402.	Regional ileocolitis	Inflammatory bowel disease
Inflammatory Bowel disease	J40z.	Regional enteritis NOS	Inflammatory bowel disease
Inflammatory Bowel disease	J41..	Idiopathic proctocolitis	Inflammatory bowel disease
Inflammatory Bowel disease	J410.	Ulcerative proctocolitis	Inflammatory bowel disease
Inflammatory Bowel disease	J4100	Ulcerative ileocolitis	Inflammatory bowel disease
Inflammatory Bowel disease	J4101	Ulcerative colitis	Inflammatory bowel disease

Table S3.1: Primary care READ codes used to identify patients with trial indications

Condition	Read.code	Description	condition_rename
Inflammatory Bowel disease	J4102	Ulcerative rectosigmoiditis	Inflammatory bowel disease
Inflammatory Bowel disease	J4103	Ulcerative proctitis	Inflammatory bowel disease
Inflammatory Bowel disease	J4104	Exacerbation of ulcerative colitis	Inflammatory bowel disease
Inflammatory Bowel disease	J410z	Ulcerative proctocolitis NOS	Inflammatory bowel disease
Inflammatory Bowel disease	J411.	Ulcerative (chronic) enterocolitis	Inflammatory bowel disease
Inflammatory Bowel disease	J412.	Ulcerative (chronic) ileocolitis	Inflammatory bowel disease
Inflammatory Bowel disease	Jyu40	[X]Other Crohn’s disease	Inflammatory bowel disease
Inflammatory Bowel disease	Jyu41	[X]Other ulcerative colitis	Inflammatory bowel disease
Inflammatory Bowel disease	N0310	Arthropathy in ulcerative colitis	Inflammatory bowel disease
Inflammatory Bowel disease	N0454	Juvenile arthritis in ulcerative colitis	Inflammatory bowel disease
Inflammatory Bowel disease	J08z9	Orofacial Crohn’s disease	Inflammatory bowel disease
Inflammatory Bowel disease	J40..	Regional enteritis - Crohn’s disease	Inflammatory bowel disease
Inflammatory Bowel disease	J4002	Crohn’s disease of the terminal ileum	Inflammatory bowel disease
Inflammatory Bowel disease	J4003	Crohn’s disease of the ileum unspecified	Inflammatory bowel disease
Inflammatory Bowel disease	J4004	Crohn’s disease of the ileum NOS	Inflammatory bowel disease
Inflammatory Bowel disease	J4005	Exacerbation of Crohn’s disease of small intestine	Inflammatory bowel disease
Inflammatory Bowel disease	J400z	Crohn’s disease of the small bowel NOS	Inflammatory bowel disease
Inflammatory Bowel disease	J4012	Exacerbation of Crohn’s disease of large intestine	Inflammatory bowel disease
Inflammatory Bowel disease	J401z	Crohn’s disease of the large bowel NOS	Inflammatory bowel disease
Inflammatory Bowel disease	Jyu40	[X]Other Crohn’s disease	Inflammatory bowel disease
Inflammatory Bowel disease	N0311	Arthropathy in Crohn’s disease	Inflammatory bowel disease
Inflammatory Bowel disease	N0453	Juvenile arthritis in Crohn’s disease	Inflammatory bowel disease
Migraine	F26..	Migraine	Migraine
Migraine	XaXkr	Migraine induced by oestrogen contraceptive	Migraine
Migraine	F261.	Migraine without aura	Migraine
Migraine	F261z	Common migraine NOS	Migraine
Migraine	F260.	Migraine with aura	Migraine
Migraine	X007J	Migraine with typical aura	Migraine
Migraine	X007K	Migraine with prolonged aura	Migraine
Migraine	F26y0	Hemiplegic migraine	Migraine

Table S3.1: Primary care READ codes used to identify patients with trial indications

Condition	Read.code	Description	condition_rename
Migraine	X007L	Familial hemiplegic migraine	Migraine
Migraine	X007M	Non-familial hemiplegic migraine	Migraine
Migraine	F2623	Basilar migraine	Migraine
Migraine	X007N	Migraine aura without headache	Migraine
Migraine	F2624	Ophthalmic migraine	Migraine
Migraine	F26y1	Ophthalmoplegic migraine	Migraine
Migraine	X007O	Retinal migraine	Migraine
Migraine	X007Q	Alternating hemiplegia of childhood	Migraine
Migraine	X007R	Status migrainosus	Migraine
Migraine	X007S	Migraine with ischaemic complication	Migraine
Migraine	Xa07H	Migraine - menstrual	Migraine
Migraine	F2610	Atypical migraine	Migraine
Migraine	F262.	Migraine variants	Migraine
Migraine	F262z	Migraine variant NOS	Migraine
Migraine	F26y.	Other forms of migraine	Migraine
Migraine	F26yz	Other forms of migraine NOS	Migraine
Migraine	F26y3	Complicated migraine	Migraine
Migraine	F26z.	Migraine NOS	Migraine
Migraine	Fyu53	[X]Other migraine	Migraine
Migraine	1474	H/O: migraine	Migraine
Migraine	XaXkv	H/O migraine with aura	Migraine
Migraine	F260.	Classical migraine	Migraine
Migraine	X007J	Migraine with typical aura	Migraine
Migraine	X007K	Migraine with prolonged aura	Migraine
Migraine	F26y0	Hemiplegic migraine	Migraine
Migraine	X007L	Familial hemiplegic migraine	Migraine
Migraine	X007M	Non-familial hemiplegic migraine	Migraine
Migraine	F2623	Basilar migraine	Migraine
Migraine	X007N	Migraine aura without headache	Migraine
Migraine	F2624	Ophthalmic migraine	Migraine
Osteoporosis	585O.00	Quantitative ultrasound scan of heel - result osteoporotic	Osteoporosis
Osteoporosis	58E4.00	Forearm DXA scan result osteoporotic	Osteoporosis
Osteoporosis	58E8.00	Heel DXA scan T score	Osteoporosis
Osteoporosis	58EA.00	Heel DXA scan result osteoporotic	Osteoporosis
Osteoporosis	58EA.00	Heel DXA scan result osteoporotic	Osteoporosis
Osteoporosis	58EE.00	Hip DXA scan T score	Osteoporosis
Osteoporosis	58EG.00	Hip DXA scan result osteoporotic	Osteoporosis
Osteoporosis	58EG.00	Hip DXA scan result osteoporotic	Osteoporosis
Osteoporosis	58EK.00	Lumbar spine DXA scan T score	Osteoporosis
Osteoporosis	58EM.00	Lumbar DXA scan result osteoporotic	Osteoporosis
Osteoporosis	58EM.00	Lumbar DXA scan result osteoporotic	Osteoporosis
Osteoporosis	58ES.00	Femoral neck DEXA scan T score	Osteoporosis
Osteoporosis	58EV.00	Femoral neck DEXA scan result osteoporotic	Osteoporosis
Osteoporosis	7230A	OSTEOPOROSIS	Osteoporosis
Osteoporosis	7230B	OSTEOPOROSIS SENILIS	Osteoporosis
Osteoporosis	7230D	VERTEBRAL OSTEOPOROSIS	Osteoporosis
Osteoporosis	7230PM	OSTEOPOROSIS POSTMENOPAUSAL	Osteoporosis
Osteoporosis	7230PT	OSTEOPOROSIS POST-TRAUMATIC	Osteoporosis
Osteoporosis	N330.00	Osteoporosis	Osteoporosis
Osteoporosis	N330.00	Osteoporosis	Osteoporosis

Table S3.1: Primary care READ codes used to identify patients with trial indications

Condition	Read.code	Description	condition_rename
Osteoporosis	N330000	Osteoporosis; unspecified	Osteoporosis
Osteoporosis	N330000	Osteoporosis; unspecified	Osteoporosis
Osteoporosis	N330100	Senile osteoporosis	Osteoporosis
Osteoporosis	N330100	Senile osteoporosis	Osteoporosis
Osteoporosis	N330200	Postmenopausal osteoporosis	Osteoporosis
Osteoporosis	N330200	Postmenopausal osteoporosis	Osteoporosis
Osteoporosis	N330300	Idiopathic osteoporosis	Osteoporosis
Osteoporosis	N330300	Idiopathic osteoporosis	Osteoporosis
Osteoporosis	N330400	Dissuse osteoporosis	Osteoporosis
Osteoporosis	N330400	Dissuse osteoporosis	Osteoporosis
Osteoporosis	N330500	Drug-induced osteoporosis	Osteoporosis
Osteoporosis	N330500	Drug-induced osteoporosis	Osteoporosis
Osteoporosis	N330600	Postoophorectomy osteoporosis	Osteoporosis
Osteoporosis	N330600	Postoophorectomy osteoporosis	Osteoporosis
Osteoporosis	N330700	Postsurgical malabsorption osteoporosis	Osteoporosis
Osteoporosis	N330700	Postsurgical malabsorption osteoporosis	Osteoporosis
Osteoporosis	N330800	Localized osteoporosis - Lequesne	Osteoporosis
Osteoporosis	N330800	Localized osteoporosis - Lequesne	Osteoporosis
Osteoporosis	N330900	Osteoporosis in multiple myelomatosis	Osteoporosis
Osteoporosis	N330900	Osteoporosis in multiple myelomatosis	Osteoporosis
Osteoporosis	N330A00	Osteoporosis in endocrine disorders	Osteoporosis
Osteoporosis	N330A00	Osteoporosis in endocrine disorders	Osteoporosis
Osteoporosis	N330B00	Vertebral osteoporosis	Osteoporosis
Osteoporosis	N330B00	Vertebral osteoporosis	Osteoporosis
Osteoporosis	N330C00	Osteoporosis localized to spine	Osteoporosis
Osteoporosis	N330C00	Osteoporosis localized to spine	Osteoporosis
Osteoporosis	N330D00	Osteoporosis due to corticosteroids	Osteoporosis
Osteoporosis	N330D00	Osteoporosis due to corticosteroids	Osteoporosis
Osteoporosis	N330z00	Osteoporosis NOS	Osteoporosis
Osteoporosis	N330z00	Osteoporosis NOS	Osteoporosis
Osteoporosis	N331200	Postoophorectomy osteoporosis with pathological fracture	Osteoporosis
Osteoporosis	N331200	Postoophorectomy osteoporosis with pathological fracture	Osteoporosis
Osteoporosis	N331300	Osteoporosis of disuse with pathological fracture	Osteoporosis
Osteoporosis	N331300	Osteoporosis of disuse with pathological fracture	Osteoporosis
Osteoporosis	N331400	Postsurgical malabsorption osteoporosis with path fracture	Osteoporosis
Osteoporosis	N331500	Drug-induced osteoporosis with pathological fracture	Osteoporosis
Osteoporosis	N331600	Idiopathic osteoporosis with pathological fracture	Osteoporosis
Osteoporosis	N331600	Idiopathic osteoporosis with pathological fracture	Osteoporosis
Osteoporosis	N331800	Osteoporosis + pathological fracture lumbar vertebrae	Osteoporosis
Osteoporosis	N331900	Osteoporosis + pathological fracture thoracic vertebrae	Osteoporosis
Osteoporosis	N331A00	Osteoporosis + pathological fracture	Osteoporosis

Table S3.1: Primary care READ codes used to identify patients with trial indications

Condition	Read.code	Description	condition_rename
		cervical vertebrae	
Osteoporosis	N331B00	Postmenopausal osteoporosis with pathological fracture	Osteoporosis
Osteoporosis	N331M00	Fragility fracture due to unspecified osteoporosis	Osteoporosis
Osteoporosis	N331N00	Fragility fracture	Osteoporosis
Osteoporosis	NyuB000	[X]Other osteoporosis with pathological fracture	Osteoporosis
Osteoporosis	NyuB000	[X]Other osteoporosis with pathological fracture	Osteoporosis
Osteoporosis	NyuB100	[X]Other osteoporosis	Osteoporosis
Osteoporosis	NyuB100	[X]Other osteoporosis	Osteoporosis
Osteoporosis	NyuB200	[X]Osteoporosis in other disorders classified elsewhere	Osteoporosis
Osteoporosis	NyuB800	[X]Unspecified osteoporosis with pathological fracture	Osteoporosis
Osteoporosis	NyuB800	[X]Unspecified osteoporosis with pathological fracture	Osteoporosis
Parkinson’s disease (excluding secondary)	147F.	History of Parkinson’s disease	Parkinson’s disease (excluding secondary)
Parkinson’s disease (excluding secondary)	297A.	O/E - Parkinsonian tremor	Parkinson’s disease (excluding secondary)
Parkinson’s disease (excluding secondary)	2987.	O/E -Parkinson flexion posture	Parkinson’s disease (excluding secondary)
Parkinson’s disease (excluding secondary)	2994.	O/E-festination-Parkinson gait	Parkinson’s disease (excluding secondary)
Parkinson’s disease (excluding secondary)	Eu023	[X]Dementia in Parkinson’s disease	Parkinson’s disease (excluding secondary)
Parkinson’s disease (excluding secondary)	F11x9	Cerebral degeneration in Parkinson’s disease	Parkinson’s disease (excluding secondary)
Parkinson’s disease (excluding secondary)	F12..	Parkinson’s disease	Parkinson’s disease (excluding secondary)
Parkinson’s disease (excluding secondary)	F120.	Paralysis agitans	Parkinson’s disease (excluding secondary)
Parkinson’s disease (excluding secondary)	F12z.	Parkinson’s disease NOS	Parkinson’s disease (excluding secondary)
Parkinson’s disease (excluding secondary)	F1303	Parkinsonism with orthostatic hypotension	Parkinson’s disease (excluding secondary)
Parkinson’s disease (excluding secondary)	Fyu22	[X]Parkinsonism in diseases classified elsewhere	Parkinson’s disease (excluding secondary)
Psoriasis	14F2.	H/O: psoriasis	Psoriasis
Psoriasis	M160.	Psoriatic arthropathy	Psoriasis
Psoriasis	M1600	Psoriasis spondylitica	Psoriasis
Psoriasis	M1601	Distal interphalangeal psoriatic arthropathy	Psoriasis
Psoriasis	M160z	Psoriatic arthropathy NOS	Psoriasis
Psoriasis	M161.	Other psoriasis	Psoriasis
Psoriasis	M1610	Psoriasis unspecified	Psoriasis
Psoriasis	M1611	Psoriasis annularis	Psoriasis
Psoriasis	M1612	Psoriasis circinata	Psoriasis
Psoriasis	M1613	Psoriasis diffusa	Psoriasis
Psoriasis	M1614	Psoriasis discoidea	Psoriasis
Psoriasis	M1615	Psoriasis geographica	Psoriasis

Table S3.1: Primary care READ codes used to identify patients with trial indications

Condition	Read.code	Description	condition_rename
Psoriasis	M1616	Guttate psoriasis	Psoriasis
Psoriasis	M1617	Psoriasis gyrata	Psoriasis
Psoriasis	M1618	Psoriasis inveterata	Psoriasis
Psoriasis	M1619	Psoriasis ostracea	Psoriasis
Psoriasis	M161A	Psoriasis palmaris	Psoriasis
Psoriasis	M161B	Psoriasis plantaris	Psoriasis
Psoriasis	M161C	Psoriasis punctata	Psoriasis
Psoriasis	M161D	Pustular psoriasis	Psoriasis
Psoriasis	M161E	Psoriasis universalis	Psoriasis
Psoriasis	M161F	Psoriasis vulgaris	Psoriasis
Psoriasis	M161G	Acrodermatitis continua	Psoriasis
Psoriasis	M161H	Erythrodermic psoriasis	Psoriasis
Psoriasis	M161z	Psoriasis NOS	Psoriasis
Psoriasis	M16y.	Other psoriasis and similar disorders	Psoriasis
Psoriasis	M16y0	Scalp psoriasis	Psoriasis
Psoriasis	M16z.	Psoriasis and similar disorders NOS	Psoriasis
Psoriasis	Myu30	[X]Other psoriasis	Psoriasis
Psoriasis	N0452	Juvenile arthritis in psoriasis	Psoriasis
Psoriasis	Nyu13	[X]Other psoriatic arthropathies	Psoriasis
Psoriatic arthropathy	N0452	Juvenile arthritis in psoriasis	Psoriatic arthropathy
Psoriatic arthropathy	M1600	Psoriasis spondylitica	Psoriatic arthropathy
Psoriatic arthropathy	M1601	Distal interphalangeal psoriatic arthropathy	Psoriatic arthropathy
Psoriatic arthropathy	M1602	Arthritis mutilans	Psoriatic arthropathy
Psoriatic arthropathy	Nyu13	[X]Other psoriatic arthropathies	Psoriatic arthropathy
Psoriatic arthropathy	M160.	Psoriatic arthropathy	Psoriatic arthropathy
Psoriatic arthropathy	M160z	Psoriatic arthropathy NOS	Psoriatic arthropathy
COPD	66YB.	Chronic obstructive pulmonary disease monitoring	Pulmonary Disease, Chronic Obstructive
COPD	66Yd.	COPD accident and emergency attendance since last visit	Pulmonary Disease, Chronic Obstructive
COPD	66YD.	Chronic obstructive pulmonary disease monitoring due	Pulmonary Disease, Chronic Obstructive
COPD	66Ye.	Emergency COPD admission since last appointment	Pulmonary Disease, Chronic Obstructive
COPD	66Yf.	Number of COPD exacerbations in past year	Pulmonary Disease, Chronic Obstructive
COPD	66Yg.	Chronic obstructive pulmonary disease disturbs sleep	Pulmonary Disease, Chronic Obstructive
COPD	66Yh.	Chronic obstructive pulmonary disease does not disturb sleep	Pulmonary Disease, Chronic Obstructive
COPD	66Yi.	Multiple COPD emergency hospital admissions	Pulmonary Disease, Chronic Obstructive
COPD	66YI.	COPD self-management plan given	Pulmonary Disease, Chronic Obstructive
COPD	66YL.	Chronic obstructive pulmonary disease follow-up	Pulmonary Disease, Chronic Obstructive
COPD	66YM.	Chronic obstructive pulmonary disease annual review	Pulmonary Disease, Chronic Obstructive
COPD	66YS.	Chronic obstructive pulmonary disease monitoring by nurse	Pulmonary Disease, Chronic Obstructive
COPD	66YT.	Chronic obstructive pulmonary disease monitoring by doctor	Pulmonary Disease, Chronic Obstructive

Table S3.1: Primary care READ codes used to identify patients with trial indications

Condition	Read.code	Description	condition_rename
COPD	8CR1.	Chronic obstructive pulmonary disease clini management plan	Pulmonary Disease, Chronic Obstructive
COPD	H3…	Chronic obstructive pulmonary disease	Pulmonary Disease, Chronic Obstructive
COPD	H3121	Emphysematous bronchitis	Pulmonary Disease, Chronic Obstructive
COPD	H3122	Acute exacerbation of chronic obstructive airways disease	Pulmonary Disease, Chronic Obstructive
COPD	H312z	Obstructive chronic bronchitis NOS	Pulmonary Disease, Chronic Obstructive
COPD	H32..	Emphysema	Pulmonary Disease, Chronic Obstructive
COPD	H320.	Chronic bullous emphysema	Pulmonary Disease, Chronic Obstructive
COPD	H3200	Segmental bullous emphysema	Pulmonary Disease, Chronic Obstructive
COPD	H3201	Zonal bullous emphysema	Pulmonary Disease, Chronic Obstructive
COPD	H3202	Giant bullous emphysema	Pulmonary Disease, Chronic Obstructive
COPD	H3203	Bullous emphysema with collapse	Pulmonary Disease, Chronic Obstructive
COPD	H320z	Chronic bullous emphysema NOS	Pulmonary Disease, Chronic Obstructive
COPD	H321.	Panlobular emphysema	Pulmonary Disease, Chronic Obstructive
COPD	H322.	Centrilobular emphysema	Pulmonary Disease, Chronic Obstructive
COPD	H32y.	Other emphysema	Pulmonary Disease, Chronic Obstructive
COPD	H32y0	Acute vesicular emphysema	Pulmonary Disease, Chronic Obstructive
COPD	H32y1	Atrophic (senile) emphysema	Pulmonary Disease, Chronic Obstructive
COPD	H32y2	MacLeod’s unilateral emphysema	Pulmonary Disease, Chronic Obstructive
COPD	H32yz	Other emphysema NOS	Pulmonary Disease, Chronic Obstructive
COPD	H32z.	Emphysema NOS	Pulmonary Disease, Chronic Obstructive
COPD	H36..	Mild chronic obstructive pulmonary disease	Pulmonary Disease, Chronic Obstructive
COPD	H37..	Moderate chronic obstructive pulmonary disease	Pulmonary Disease, Chronic Obstructive
COPD	H38..	Severe chronic obstructive pulmonary disease	Pulmonary Disease, Chronic Obstructive
COPD	H39..	Very severe chronic obstructive pulmonary disease	Pulmonary Disease, Chronic Obstructive
COPD	H3y..	Other specified chronic obstructive airways disease	Pulmonary Disease, Chronic Obstructive
COPD	H3y0.	Chronic obstruct pulmonary dis with acute lower resp infectn	Pulmonary Disease, Chronic Obstructive
COPD	H3y1.	Chron obstruct pulmonary dis wth	Pulmonary Disease,

Table S3.1: Primary care READ codes used to identify patients with trial indications

Condition	Read.code	Description	condition_rename
		acute exacerbation, unspec	Chronic Obstructive
COPD	H3z..	Chronic obstructive airways disease NOS	Pulmonary Disease, Chronic Obstructive
COPD	H4640	Chronic emphysema due to chemical fumes	Pulmonary Disease, Chronic Obstructive
COPD	Hyu3.	[X]Chronic lower respiratory diseases	Pulmonary Disease, Chronic Obstructive
COPD	Hyu30	[X]Other emphysema	Pulmonary Disease, Chronic Obstructive
COPD	Hyu31	[X]Other specified chronic obstructive pulmonary disease	Pulmonary Disease, Chronic Obstructive
Restless leg	F13z2	Ekbom syndrome	Restless legs syndrome
Rheumatoir arthritis	14G1.	H/O: rheumatoid arthritis	Rheumatoid arthritis
Rheumatoir arthritis	F3712	Polyneuropathy in rheumatoid arthritis	Rheumatoid arthritis
Rheumatoir arthritis	F3964	Myopathy due to rheumatoid arthritis	Rheumatoid arthritis
Rheumatoir arthritis	G5yA.	Rheumatoid carditis	Rheumatoid arthritis
Rheumatoir arthritis	G5y8.	Rheumatoid myocarditis	Rheumatoid arthritis
Rheumatoir arthritis	H570.	Rheumatoid lung	Rheumatoid arthritis
Rheumatoir arthritis	N04..	Rheumatoid arthritis and other inflammatory polyarthropathy	Rheumatoid arthritis
Rheumatoir arthritis	N040.	Rheumatoid arthritis	Rheumatoid arthritis
Rheumatoir arthritis	N0400	Rheumatoid arthritis of cervical spine	Rheumatoid arthritis
Rheumatoir arthritis	N0401	Other rheumatoid arthritis of spine	Rheumatoid arthritis
Rheumatoir arthritis	N0402	Rheumatoid arthritis of shoulder	Rheumatoid arthritis
Rheumatoir arthritis	N0403	Rheumatoid arthritis of sternoclavicular joint	Rheumatoid arthritis
Rheumatoir arthritis	N0404	Rheumatoid arthritis of acromioclavicular joint	Rheumatoid arthritis
Rheumatoir arthritis	N0405	Rheumatoid arthritis of elbow	Rheumatoid arthritis
Rheumatoir arthritis	N0406	Rheumatoid arthritis of distal radio-ulnar joint	Rheumatoid arthritis
Rheumatoir arthritis	N0407	Rheumatoid arthritis of wrist	Rheumatoid arthritis
Rheumatoir arthritis	N0408	Rheumatoid arthritis of MCP joint	Rheumatoid arthritis
Rheumatoir arthritis	N0409	Rheumatoid arthritis of PIP joint of finger	Rheumatoid arthritis
Rheumatoir arthritis	N040A	Rheumatoid arthritis of DIP joint of finger	Rheumatoid arthritis
Rheumatoir arthritis	N040B	Rheumatoid arthritis of hip	Rheumatoid arthritis
Rheumatoir arthritis	N040C	Rheumatoid arthritis of sacro-iliac joint	Rheumatoid arthritis
Rheumatoir arthritis	N040D	Rheumatoid arthritis of knee	Rheumatoid arthritis
Rheumatoir arthritis	N040E	Rheumatoid arthritis of tibio-fibular joint	Rheumatoid arthritis
Rheumatoir arthritis	N040F	Rheumatoid arthritis of ankle	Rheumatoid arthritis
Rheumatoir arthritis	N040G	Rheumatoid arthritis of subtalar joint	Rheumatoid arthritis
Rheumatoir arthritis	N040H	Rheumatoid arthritis of talonavicular joint	Rheumatoid arthritis
Rheumatoir arthritis	N040J	Rheumatoid arthritis of other tarsal joint	Rheumatoid arthritis
Rheumatoir arthritis	N040K	Rheumatoid arthritis of 1st MTP joint	Rheumatoid arthritis
Rheumatoir arthritis	N040L	Rheumatoid arthritis of lesser MTP joint	Rheumatoid arthritis
Rheumatoir arthritis	N040M	Rheumatoid arthritis of IP joint of toe	Rheumatoid arthritis
Rheumatoir arthritis	N040N	Rheumatoid vasculitis	Rheumatoid arthritis

Table S3.1: Primary care READ codes used to identify patients with trial indications

Condition	Read.code	Description	condition_rename
Rheumatoir arthritis	N040P	Seronegative rheumatoid arthritis	Rheumatoid arthritis
Rheumatoir arthritis	N040Q	Rheumatoid bursitis	Rheumatoid arthritis
Rheumatoir arthritis	N040R	Rheumatoid nodule	Rheumatoid arthritis
Rheumatoir arthritis	N040S	Rheumatoid arthritis - multiple joint	Rheumatoid arthritis
Rheumatoir arthritis	N040T	Flare of rheumatoid arthritis	Rheumatoid arthritis
Rheumatoir arthritis	N041.	Felty's syndrome	Rheumatoid arthritis
Rheumatoir arthritis	N042.	Other rheumatoid arthropathy + visceral/systemic involvement	Rheumatoid arthritis
Rheumatoir arthritis	N0421	Rheumatoid lung disease	Rheumatoid arthritis
Rheumatoir arthritis	N0422	Rheumatoid nodule	Rheumatoid arthritis
Rheumatoir arthritis	N042z	Rheumatoid arthropathy + visceral/systemic involvement NOS	Rheumatoid arthritis
Rheumatoir arthritis	N043.	Juvenile rheumatoid arthritis - Still's disease	Rheumatoid arthritis
Rheumatoir arthritis	N0430	Juvenile rheumatoid arthropathy unspecified	Rheumatoid arthritis
Rheumatoir arthritis	N0431	Acute polyarticular juvenile rheumatoid arthritis	Rheumatoid arthritis
Rheumatoir arthritis	N0432	Pauciarticular juvenile rheumatoid arthritis	Rheumatoid arthritis
Rheumatoir arthritis	N0433	Monarticular juvenile rheumatoid arthritis	Rheumatoid arthritis
Rheumatoir arthritis	N043z	Juvenile rheumatoid arthritis NOS	Rheumatoid arthritis
Rheumatoir arthritis	N047.	Seropositive errosive rheumatoid arthritis	Rheumatoid arthritis
Rheumatoir arthritis	N04X.	Seropositive rheumatoid arthritis, unspecified	Rheumatoid arthritis
Rheumatoir arthritis	N04y2	Adult-onset Still's disease	Rheumatoid arthritis
Rheumatoir arthritis	N0455	Juvenile rheumatoid arthritis	Rheumatoid arthritis
SLE	Nyu43	[X]Other forms of systemic lupus erythematosus	Systemic Lupus Erythematosus
SLE	H57y4	Lung disease with systemic lupus erythematosus	Systemic Lupus Erythematosus
SLE	K01x4	Nephrotic syndrome in systemic lupus erythematosus	Systemic Lupus Erythematosus
SLE	K0B40	Renal tubulo-interstitial disorder in SLE	Systemic Lupus Erythematosus
SLE	N000.	Systemic lupus erythematosus	Systemic Lupus Erythematosus
SLE	N0000	Disseminated lupus erythematosus	Systemic Lupus Erythematosus
SLE	N0002	Drug-induced systemic lupus erythematosus	Systemic Lupus Erythematosus
SLE	N0003	Systemic lupus erythematosus with organ or sys involv	Systemic Lupus Erythematosus
SLE	N0004	Systemic lupus erythematosus with pericarditis	Systemic Lupus Erythematosus
SLE	N000z	Systemic lupus erythematosus NOS	Systemic Lupus Erythematosus
SLE	F371.	Polyneuropathy in collagen vascular disease	Systemic Lupus Erythematosus
SLE	F3961	Myopathy due to disseminated lupus erythematosus	Systemic Lupus Erythematosus

Table S3.1: Primary care READ codes used to identify patients with trial indications

Condition	Read.code	Description	condition_rename
Thromboembolism	14A8.00	H/O: thrombo-embolism	Thromboembolism
Thromboembolism	14A8100	H/O: Deep Vein Thrombosis	Thromboembolism
Thromboembolism	14A8.11	H/O: embolism	Thromboembolism
Thromboembolism	14A8.12	H/O: thrombosis	Thromboembolism
Thromboembolism	14AC.00	H/O: pulmonary embolus	Thromboembolism
Thromboembolism	G401.00	Pulmonary embolism	Thromboembolism
Thromboembolism	G401000	Post operative pulmonary embolus	Thromboembolism
Thromboembolism	G401100	Recurrent pulmonary embolism	Thromboembolism
Thromboembolism	G401.11	Infarction - pulmonary	Thromboembolism
Thromboembolism	G401.12	Pulmonary embolus	Thromboembolism
Thromboembolism	G41y100	Thromboembolic pulmonary hypertension	Thromboembolism
Thromboembolism	G74..00	Arterial embolism and thrombosis	Thromboembolism
Thromboembolism	G740.00	Embolism and thrombosis of the abdominal aorta	Thromboembolism
Thromboembolism	G740.11	Aortic bifurcation syndrome	Thromboembolism
Thromboembolism	G740.12	Aortoiliac obstruction	Thromboembolism
Thromboembolism	G740.13	Leriche's syndrome	Thromboembolism
Thromboembolism	G740.14	Saddle embolus	Thromboembolism
Thromboembolism	G741.00	Embolism and thrombosis of the thoracic aorta	Thromboembolism
Thromboembolism	G74..11	Arterial embolus and thrombosis	Thromboembolism
Thromboembolism	G74..12	Thrombosis - arterial	Thromboembolism
Thromboembolism	G74..13	Arterial embolic and thrombotic occlusion	Thromboembolism
Thromboembolism	G742.00	Embolism and thrombosis of an arm or leg artery	Thromboembolism
Thromboembolism	G742000	Embolism and thrombosis of the brachial artery	Thromboembolism
Thromboembolism	G742100	Embolism and thrombosis of the radial artery	Thromboembolism
Thromboembolism	G742200	Embolism and thrombosis of the ulnar artery	Thromboembolism
Thromboembolism	G742300	Embolism and thrombosis of an arm artery NOS	Thromboembolism
Thromboembolism	G742400	Embolism and thrombosis of the femoral artery	Thromboembolism
Thromboembolism	G742500	Embolism and thrombosis of the popliteal artery	Thromboembolism
Thromboembolism	G742600	Embolism and thrombosis of the anterior tibial artery	Thromboembolism
Thromboembolism	G742700	Embolism and thrombosis of the dorsalis pedis artery	Thromboembolism
Thromboembolism	G742800	Embolism and thrombosis of the posterior tibial artery	Thromboembolism
Thromboembolism	G742900	Embolism and thrombosis of a leg artery NOS	Thromboembolism
Thromboembolism	G742A00	Post radiological embolism of upper limb artery	Thromboembolism
Thromboembolism	G742B00	Post radiological embolism of lower limb artery	Thromboembolism
Thromboembolism	G742z00	Peripheral arterial embolism and thrombosis NOS	Thromboembolism

Table S3.1: Primary care READ codes used to identify patients with trial indications

Condition	Read.code	Description	condition_rename
Thromboembolism	G743.00	Embolism and thrombosis of other and unspec parts aorta	Thromboembolism
Thromboembolism	G74y.00	Embolism and thrombosis of other specified artery	Thromboembolism
Thromboembolism	G74y000	Embolism and/or thrombosis of the common iliac artery	Thromboembolism
Thromboembolism	G74y100	Embolism and/or thrombosis of the internal iliac artery	Thromboembolism
Thromboembolism	G74y200	Embolism and/or thrombosis of the external iliac artery	Thromboembolism
Thromboembolism	G74y300	Embolism and thrombosis of the iliac artery unspecified	Thromboembolism
Thromboembolism	G74y500	Embolism and thrombosis of the subclavian artery	Thromboembolism
Thromboembolism	G74y600	Embolism and thrombosis of the splenic artery	Thromboembolism
Thromboembolism	G74y700	Embolism and thrombosis of the axillary artery	Thromboembolism
Thromboembolism	G74y800	Embolism and thrombosis of the coeliac artery	Thromboembolism
Thromboembolism	G74y900	Embolism and thrombosis of the hepatic artery	Thromboembolism
Thromboembolism	G74yz00	Embolism and thrombosis of other arteries NOS	Thromboembolism
Thromboembolism	G74z.00	Arterial embolism and thrombosis NOS	Thromboembolism
Thromboembolism	G801.00	Deep vein phlebitis and thrombophlebitis of the leg	Thromboembolism
Thromboembolism	G801000	Phlebitis of the femoral vein	Thromboembolism
Thromboembolism	G801100	Phlebitis of the popliteal vein	Thromboembolism
Thromboembolism	G801.11	Deep vein thrombosis	Thromboembolism
Thromboembolism	G801.12	Deep vein thrombosis; leg	Thromboembolism
Thromboembolism	G801.13	DVT - Deep vein thrombosis	Thromboembolism
Thromboembolism	G801200	Phlebitis of the anterior tibial vein	Thromboembolism
Thromboembolism	G801400	Phlebitis of the posterior tibial vein	Thromboembolism
Thromboembolism	G801500	Deep vein phlebitis of the leg unspecified	Thromboembolism
Thromboembolism	G801600	Thrombophlebitis of the femoral vein	Thromboembolism
Thromboembolism	G801700	Thrombophlebitis of the popliteal vein	Thromboembolism
Thromboembolism	G801800	Thrombophlebitis of the anterior tibial vein	Thromboembolism
Thromboembolism	G801900	Thrombophlebitis of the dorsalis pedis vein	Thromboembolism
Thromboembolism	G801A00	Thrombophlebitis of the posterior tibial vein	Thromboembolism
Thromboembolism	G801B00	Deep vein thrombophlebitis of the leg unspecified	Thromboembolism
Thromboembolism	G801C00	Deep vein thrombosis of leg related to air travel	Thromboembolism
Thromboembolism	G801D00	Deep vein thrombosis of lower limb	Thromboembolism
Thromboembolism	G801E00	Deep vein thrombosis of leg related to intravenous drug use	Thromboembolism
Thromboembolism	G801F00	Deep vein thrombosis of peroneal vein	Thromboembolism
Thromboembolism	G801G00	Recurrent deep vein thrombosis	Thromboembolism

Table S3.1: Primary care READ codes used to identify patients with trial indications

Condition	Read.code	Description	condition_rename
Thromboembolism	G801z00	Deep vein phlebitis and thrombophlebitis of the leg NOS	Thromboembolism
Thromboembolism	G802000	Thrombosis of vein of leg	Thromboembolism
Thromboembolism	G81..00	Portal vein thrombosis	Thromboembolism
Thromboembolism	G82..00	Other venous embolism and thrombosis	Thromboembolism
Thromboembolism	G820.00	Budd - Chiari syndrome (hepatic vein thrombosis)	Thromboembolism
Thromboembolism	G820.11	Hepatic vein thrombosis	Thromboembolism
Thromboembolism	G821.00	Thrombophlebitis migrans	Thromboembolism
Thromboembolism	G822.00	Embolism and thrombosis of the vena cava	Thromboembolism
Thromboembolism	G822000	Thrombosis of inferior vena cava	Thromboembolism
Thromboembolism	G823.00	Embolism and thrombosis of the renal vein	Thromboembolism
Thromboembolism	G824.00	Axillary vein thrombosis	Thromboembolism
Thromboembolism	G825.00	Thrombosis of subclavian vein	Thromboembolism
Thromboembolism	G826.00	Thrombosis of internal jugular vein	Thromboembolism
Thromboembolism	G827.00	Thrombosis of external jugular vein	Thromboembolism
Thromboembolism	G82y.00	Other embolism and thrombosis	Thromboembolism
Thromboembolism	G82z.00	Embolism and thrombosis NOS	Thromboembolism
Thromboembolism	G82z000	Embolus of vein NOS	Thromboembolism
Thromboembolism	G82z011	Embolism of vein NOS	Thromboembolism
Thromboembolism	G82z100	Thrombosis of vein NOS	Thromboembolism
Thromboembolism	G82zz00	Embolism and thrombosis NOS	Thromboembolism
Thromboembolism	G8y4.00	Postthrombotic syndrome	Thromboembolism
Thromboembolism	Gyu8200	[X]Embolism and thrombosis of other specified veins	Thromboembolism
Thromboembolism	SP12200	Post operative deep vein thrombosis	Thromboembolism
Thromboembolism	SP32100	Thromboembolism after infusion	Thromboembolism
Thromboembolism	ZV12800	[V] Personal history deep vein thrombosis	Thromboembolism
Thromboembolism	ZV12811	[V] Personal history DVT- deep vein thrombosis	Thromboembolism
Thromboembolism	ZV12900	[V] Personal history of pulmonary embolism	Thromboembolism
Osteoarthritis	X7039	Spondylosis	Osteoarthritis
Osteoarthritis	X703j	Cervical spondylosis	Osteoarthritis
Osteoarthritis	N119.	Cervical spondylosis with radiculopathy	Osteoarthritis
Osteoarthritis	N1190	Single-level cervical spondylosis with radiculopathy	Osteoarthritis
Osteoarthritis	N1191	Two-level cervical spondylosis with radiculopathy	Osteoarthritis
Osteoarthritis	N1192	Multiple-level cervical spondylosis with radiculopathy	Osteoarthritis
Osteoarthritis	XE1Ev	Cervical spondylosis with myelopathy	Osteoarthritis
Osteoarthritis	N1110	Single-level cervical spondylosis with myelopathy	Osteoarthritis
Osteoarthritis	N1111	Two-level cervical spondylosis with myelopathy	Osteoarthritis
Osteoarthritis	N1112	Multiple-level cervical spondylosis with myelopathy	Osteoarthritis

Table S3.1: Primary care READ codes used to identify patients with trial indications

Condition	Read.code	Description	condition_rename
Osteoarthritis	N11A.	Cervical spondylosis with vascular compression	Osteoarthritis
Osteoarthritis	XE1Eu	Cervical spondylosis without myelopathy	Osteoarthritis
Osteoarthritis	N1100	Single-level cervical spondylosis without myelopathy	Osteoarthritis
Osteoarthritis	N1101	Two-level cervical spondylosis without myelopathy	Osteoarthritis
Osteoarthritis	N1102	Multiple-level cervical spondylosis without myelopathy	Osteoarthritis
Osteoarthritis	N110.	Cervical spondylosis (& [without myelopathy]) or (osteoarthritis cervical spine)	Osteoarthritis
Osteoarthritis	X70Cw	Thoracic spondylosis	Osteoarthritis
Osteoarthritis	N11B.	Thoracic spondylosis with radiculopathy	Osteoarthritis
Osteoarthritis	N11B0	Single-level thoracic spondylosis with radiculopathy	Osteoarthritis
Osteoarthritis	N11B1	Two-level thoracic spondylosis with radiculopathy	Osteoarthritis
Osteoarthritis	N11B2	Multiple-level thoracic spondylosis with radiculopathy	Osteoarthritis
Osteoarthritis	N113.	Thoracic spondylosis with myelopathy	Osteoarthritis
Osteoarthritis	N1130	Single-level thoracic spondylosis with myelopathy	Osteoarthritis
Osteoarthritis	N1131	Two-level thoracic spondylosis with myelopathy	Osteoarthritis
Osteoarthritis	N1132	Multiple-level thoracic spondylosis with myelopathy	Osteoarthritis
Osteoarthritis	XE1Ew	Thoracic spondylosis without myelopathy	Osteoarthritis
Osteoarthritis	N1120	Single-level thoracic spondylosis without myelopathy	Osteoarthritis
Osteoarthritis	N1121	Two-level thoracic spondylosis without myelopathy	Osteoarthritis
Osteoarthritis	N1122	Multiple-level thoracic spondylosis without myelopathy	Osteoarthritis
Osteoarthritis	N112.	Thoracic spondylosis (& [without myelopathy])	Osteoarthritis
Osteoarthritis	XaE3C	Lumbosacral spondylosis	Osteoarthritis
Osteoarthritis	X703k	Lumbar spondylosis	Osteoarthritis
Osteoarthritis	N11C.	Lumbosacral spondylosis with radiculopathy	Osteoarthritis
Osteoarthritis	N11C0	Single-level lumbosacral spondylosis with radiculopathy	Osteoarthritis
Osteoarthritis	N11C1	Two-level lumbosacral spondylosis with radiculopathy	Osteoarthritis
Osteoarthritis	N11C2	Multiple-level lumbosacral spondylosis with radiculopathy	Osteoarthritis
Osteoarthritis	N115.	Lumbosacral spondylosis with myelopathy	Osteoarthritis
Osteoarthritis	N1150	Single-level lumbosacral spondylosis with myelopathy	Osteoarthritis

Table S3.1: Primary care READ codes used to identify patients with trial indications

Condition	Read.code	Description	condition_rename
Osteoarthritis	N1151	Two-level lumbosacral spondylosis with myelopathy	Osteoarthritis
Osteoarthritis	N1152	Multiple-level lumbosacral spondylosis with myelopathy	Osteoarthritis
Osteoarthritis	XE1Ex	Lumbosacral spondylosis without myelopathy	Osteoarthritis
Osteoarthritis	N1140	Single-level lumbosacral spondylosis without myelopathy	Osteoarthritis
Osteoarthritis	N1141	Two-level lumbosacral spondylosis without myelopathy	Osteoarthritis
Osteoarthritis	N1142	Multiple-level lumbosacral spondylosis without myelopathy	Osteoarthritis
Osteoarthritis	N11y.	Other spondyloses and allied disorders	Osteoarthritis
Osteoarthritis	Nyu62	[X]Other spondylosis with myelopathy	Osteoarthritis
Osteoarthritis	Nyu63	[X]Other spondylosis with radiculopathy	Osteoarthritis
Osteoarthritis	Nyu64	[X]Other spondylosis	Osteoarthritis
Osteoarthritis	XE1Ey	Spondylosis NOS	Osteoarthritis
Osteoarthritis	N11z0	Spondylosis without myelopathy, NOS	Osteoarthritis
Osteoarthritis	N11z1	Spondylosis with myelopathy, NOS	Osteoarthritis
Osteoarthritis	N114.	(Lumbosacral spondylosis [& without myelopathy]) or (degeneration of lumbar spine)	Osteoarthritis
Osteoarthritis	N11z.	(Spondylosis NOS) or (osteoarthritis spine)	Osteoarthritis
Osteoarthritis	XE1HM	(Spondyloses: [cervical] or [lumbar] or [sacral]) or (arthritis - spine) or (osteoarthritis - spine)	Osteoarthritis
Osteoarthritis	X703A	Osteoarthritis of spinal facet joint	Osteoarthritis
Osteoarthritis	X703F	Osteoarthritis of first carpometacarpal joint	Osteoarthritis
Osteoarthritis	X703G	Osteoarthritis of finger joint	Osteoarthritis
Osteoarthritis	X703H	Osteoarthritis of distal interphalangeal joint	Osteoarthritis
Osteoarthritis	N05zH	Osteoarthritis NOS, of distal interphalangeal joint of finger	Osteoarthritis
Osteoarthritis	X703I	Osteoarthritis of proximal interphalangeal joint	Osteoarthritis
Osteoarthritis	N05zG	Osteoarthritis NOS, of proximal interphalangeal joint of finger	Osteoarthritis
Osteoarthritis	X703J	Osteoarthritis of metacarpophalangeal joint of finger	Osteoarthritis
Osteoarthritis	X7035	Finger osteoarthritis NOS	Osteoarthritis
Osteoarthritis	X703K	Osteoarthritis of hip	Osteoarthritis
Osteoarthritis	N05zJ	Osteoarthritis NOS, of hip	Osteoarthritis
Osteoarthritis	X703L	Osteoarthritis of knee	Osteoarthritis
Osteoarthritis	XaYQD	Patellofemoral osteoarthritis	Osteoarthritis
Osteoarthritis	N05zL	Osteoarthritis NOS, of knee	Osteoarthritis
Osteoarthritis	N0514	Localised, primary osteoarthritis of the hand	Osteoarthritis
Osteoarthritis	N0524	Localised, secondary osteoarthritis of the hand	Osteoarthritis
Osteoarthritis	N0534	Localised osteoarthritis, unspecified, of	Osteoarthritis

Table S3.1: Primary care READ codes used to identify patients with trial indications

Condition	Read.code	Description	condition_rename
		the hand	
Osteoarthritis	N0539	Arthrosis of first carpometacarpal joint, unspecified	Osteoarthritis
Osteoarthritis	XM1NQ	Osteoarthritis of metacarpophalangeal joint	Osteoarthritis
Osteoarthritis	Xa3gQ	Osteoarthritis - hand joint	Osteoarthritis
Osteoarthritis	N0535	(Otto's pelvis) or (hip osteoarthitis NOS) or (localised osteoarthritis, unspecified, of the pelvic region and thigh)	Osteoarthritis
Osteoarthritis	N0507	Heberden's nodes with arthropathy	Osteoarthritis
Osteoarthritis	N0503	Bouchard's nodes with arthropathy	Osteoarthritis
Osteoarthritis	XE1DW	Generalised osteoarthritis of the hand	Osteoarthritis
Osteoarthritis	N0501	(Heberdens' nodes) or (Bouchards' nodes) or (generalised osteoarthritis of the hand)	Osteoarthritis
Osteoarthritis	N0614	Traumatic arthropathy of the hand	Osteoarthritis
Osteoarthritis	N061G	Traumatic arthropathy of metacarpophalangeal joint	Osteoarthritis
Osteoarthritis	N061H	Traumatic arthropathy of proximal interphalangeal joint of finger	Osteoarthritis
Osteoarthritis	N061J	Traumatic arthropathy of distal interphalangeal joint of finger	Osteoarthritis
Osteoarthritis	N061K	Traumatic arthropathy-hip	Osteoarthritis
Osteoarthritis	N061M	Traumatic arthropathy-knee	Osteoarthritis
Osteoarthritis	N11y2	Neuropathic spondylopathy	Osteoarthritis
Osteoarthritis	N0604	Kaschin-Beck disease of the hand	Osteoarthritis
Osteoarthritis	N0544	Oligoarticular osteoarthritis, unspecified, of hand	Osteoarthritis
Osteoarthritis	N05zF	Osteoarthritis NOS, of metacarpophalangeal joint	Osteoarthritis
Osteoarthritis	N05zL	Osteoarthritis NOS, of knee	Osteoarthritis
Osteoarthritis	XE1Dd	Osteoarthritis NOS, of the hand	Osteoarthritis
Osteoarthritis	X7034	Thumb osteoarthritis NOS	Osteoarthritis
Osteoarthritis	N05z4	Osteoarthritis NOS: [hand] or [finger] or [thumb]	Osteoarthritis
Osteoarthritis	N05z5	Osteoarthritis NOS: [pelvic region and/or thigh] or [hip]	Osteoarthritis
Osteoarthritis	XE1DV	Osteoarthrosis	Osteoarthritis
Osteoarthritis	XaIna	Exacerbation of osteoarthritis	Osteoarthritis
Osteoarthritis	X703Q	Secondary osteoarthritis	Osteoarthritis
Osteoarthritis	N050.	Generalised osteoarthritis	Osteoarthritis
Osteoarthritis	N0500	Generalised osteoarthritis of unspecified site	Osteoarthritis
Osteoarthritis	N0502	Generalised osteoarthritis of multiple sites	Osteoarthritis
Osteoarthritis	N050z	Generalised osteoarthritis NOS	Osteoarthritis
Osteoarthritis	N0505	Secondary multiple arthrosis	Osteoarthritis
Osteoarthritis	N0619	Traumatic arthropathy of multiple sites	Osteoarthritis
Osteoarthritis	N0609	Kaschin-Beck disease of multiple sites	Osteoarthritis
Osteoarthritis	X7038	Idiopathic osteoarthritis	Osteoarthritis
Osteoarthritis	N0506	Erosive osteoarthrosis	Osteoarthritis
Osteoarthritis	N054.	Oligoarticular osteoarthritis,	Osteoarthritis

Table S3.1: Primary care READ codes used to identify patients with trial indications

Condition	Read.code	Description	condition_rename
		unspecified	
Osteoarthritis	N0540	Oligoarticular osteoarthritis, unspecified, of unspecified sites	Osteoarthritis
Osteoarthritis	N0545	Oligoarticular osteoarthritis, unspecified, of the pelvic region and thigh	Osteoarthritis
Osteoarthritis	N0549	Oligoarticular osteoarthritis, unspecified, of multiple sites	Osteoarthritis
Osteoarthritis	N054z	Osteoarthritis of more than one site, unspecified, NOS	Osteoarthritis
Osteoarthritis	XE1Da	Osteoarthritis NOS	Osteoarthritis
Osteoarthritis	XE1Gm	Osteoarthritis -multiple joint	Osteoarthritis
Osteoarthritis	N05z.	[Joint degeneration] or [osteoarthritis NOS]	Osteoarthritis
Osteoarthritis	X703E	Osteoarthritis of wrist	Osteoarthritis
Osteoarthritis	N05zE	Osteoarthritis NOS, of wrist	Osteoarthritis
Osteoarthritis	XaEGd	Localised, primary osteoarthritis of the wrist	Osteoarthritis
Osteoarthritis	N0515	Localised, primary osteoarthritis of the pelvic region and thigh	Osteoarthritis
Osteoarthritis	XE1DX	Localised, secondary osteoarthritis of the pelvic region and thigh	Osteoarthritis
Osteoarthritis	XE1DY	Localised osteoarthritis, unspecified, of the pelvic region and thigh	Osteoarthritis
Osteoarthritis	N0615	Traumatic arthropathy of the pelvic region and thigh	Osteoarthritis
Osteoarthritis	N061E	Traumatic arthropathy of distal radioulnar joint	Osteoarthritis
Osteoarthritis	N061F	Traumatic arthropathy-wrist	Osteoarthritis
Osteoarthritis	N0605	Kaschin-Beck disease of the pelvic region and thigh	Osteoarthritis
Osteoarthritis	N05zD	Osteoarthritis NOS, of distal radioulnar joint	Osteoarthritis
Osteoarthritis	N05zE	Osteoarthritis NOS, of wrist	Osteoarthritis
Osteoarthritis	XE1De	Osteoarthritis NOS, pelvic region/thigh	Osteoarthritis
Osteoarthritis	N05z3	Osteoarthritis NOS: [of the forearm] or [wrist]	Osteoarthritis
Pulmonary fibrosis	H563.00	idiopathic fibrosing alveolitis	Pulmonary fibrosis
Pulmonary fibrosis	H563.11	Hamman?ich syndrome	Pulmonary fibrosis
Pulmonary fibrosis	H563.12	cryptogenic fibrosing alveolitis	Pulmonary fibrosis
Pulmonary fibrosis	H563.13	Idiopathic pulmonary fibrosis	Pulmonary fibrosis
Pulmonary fibrosis	H563100	diffuse pulmonary fibrosis	Pulmonary fibrosis
Pulmonary fibrosis	H563200	Pulmonary fibrosis	Pulmonary fibrosis
Pulmonary fibrosis	H563z	Idiopathic fibrosing alveolitis	Pulmonary fibrosis
Pulmonary fibrosis	H563z00	idiopathic fibrosing alveolitis NOS	Pulmonary fibrosis
Pulmonary fibrosis	X102v	Usual interstitial pneumonitis	Pulmonary fibrosis
Pulmonary fibrosis	X102w	Desquamative interstitial pneum	Pulmonary fibrosis
Pulmonary fibrosis	XE0Yb	Cryptogenic fibrosing alveoliti	Pulmonary fibrosis
Pulmonary fibrosis	XE0Zr	Idiopath. fibrosing alveolitis	Pulmonary fibrosis

For migraine and for thromboembolism, primary care patents were only identified as having the indication if they were also taking anti-migraine medication (ATC code N02C) or anticoagulants (ATC code B01AA, B01AE or B01AF), respectively. These codes are shown under comorbidity definitions in the next section.
