# Supplementary material for: Representation of people with comorbidity and multimorbidity in clinical trials of novel drug therapies: an individual-level participant data analysis
Source: BMC Med. 2019 Nov 12;17:201. doi: 10.1186/s12916-019-1427-1 (PMC6849229; doi:10.1186/s12916-019-1427-1)
Supplement: Supplementary file 4 — Additional file 4. Defining-comorbidity-protocol.pdf: Protocol detailing the identification of comorbidities from clinical trial data. [file 12916_2019_1427_MOESM4_ESM.pdf]

4 Defining comorbidity - protocol

4.1 Approach to defining comorbidities

We used concomitant medications to identify comorbid conditions.

Previous studies have also used the WHO ATC criteria to define comorbid diseases, but usually in routine healthcare data with the goal being descriptive epidemiology, as in a recent paper by Huber et al.[1] We are not aware of any previous study which has used this approach for individual-level participant data from clinical trials, or to examine heterogeneity of treatment effects. To reduce non-differential misclassification bias, we have chosen definitions which we think favour specificity over sensitivity. (Table S4.2).

For each of the drug-based definitions in Table S4.2, reported concomitant medications were eligible if they were started at any time on or before starting the trial drug (or comparator) regardless of when they were stopped. Topical drugs are not included in the definitions except for M02 or S01E. Nor drugs with an inhaled or nebulised route of administration, except for R03 drugs.

This approach to defining comorbidities is a compromise between sensitivity and specificity and some misclassification is inevitable, reflecting the difficulty of inferring diagnoses from drug-usage. We have attempted to minimise the misclassification based on our understanding of clinical practice, with an emphasis on specificity over sensitivity.

For example, rather than assuming all participants taking a drug in the A02B class have an acid-related disorder (Table S4.2) we have limited this definition to exclude participants also taking non-steroidal drugs or any drug with anti-thrombotic actions (aspirin, antiplatelets, warfarin etc.). Similarly, we have not used aspirin to define cardiovascular disease because it is in widespread use for primary prevention.[2]

Moreover, while the ATC system is organised around therapeutic indications, not all indications are coded. This is because “A medicinal substance can be given more than one ATC code **ONLY** if it is available in two or more strengths or routes of administration with clearly different therapeutic uses.” For example, finasteride is classified as a dermatological drug if low-dose and as a drug for benign prostatic hyperplasia if high-dose. Therefore, for a single strength and route of administration, there is only “one code, the main indication being decided on the basis of the available information.”[3]

Moreover, the “main” WHO ATC indication is not necessarily the commonest indication. For example, in a US study of drug “mentions” in a representative database, >80% of mentions for gabapentin and amitriptyline were for off-label indications, predominantly pain.[4] Similarly, in a Canadian study of antidepressant use in primary care, amitriptyline was “almost exclusively prescribed for off-label indications” most commonly for pain, insomnia, and migraine.[5] These published findings are consistent with the clinical observation of members of our steering committee (and an independent epileptologist), that these drugs are predominantly used for pain. Despite this, the WHO ATC scheme does not include pain as an indication for these drugs, classifying gabapentin and pregabalin exclusively as antiepileptics and amitriptyline exclusively as an antidepressant.

Nor is there necessarily a code where routes/strengths do differ. For example, prochlorperazine is defined solely as an antipsychotic, despite being available in a buccal preparation for nausea. In this case the accompanying note states that “The substances in this group are sometimes used for other indications in much lower doses”.

We had initially planned to add skin disease to the list of diagnoses, however we found that topical therapies were very poorly recorded in the trial data and so have opted to drop this from the comorbid disease definitions.

A tabular summary of the drug-based comorbidity definitions given in Table 4.2, the definitive description is contained in the R code R code for comorbid disease definitions.

4.2 Incomplete ATC coding

An additional complexity is caused by the fact that for certain trials, sponsors have only provided less specific codes (eg 3 or 4-character codes) and not 5-character codes which uniquely identify each class (7-character codes identifying each agent). Where this is the case, but the drug name with or without route and indication information have been provided, we assigned each potentially-relevant drug to a WHO ATC code using the US Government drug meta-thesaurus (RXNORM). Where neither the drug name, nor sufficiently detailed drug-class information is provided, we adopted a workaround suited to each definition (Table S4.2). This had a limited impact on the overall comorbidity totals, and only applied to inflammatory, pain, urological and erectile definitions (4.1). In the case of pain and inflamamtory definitions, the broader categorisation was used. For urological and erectile definitions, the narrower categorisation was used.

Table S4.1: Proportion of participants with definition met on basis of 3/4 character ATC code

_	**YODA**	**CSDR**
Trials	37	82
Pain	253 (5.7%)	832 (5%)
Inflammatory	148 (77.5%)	308 (8.1%)
Urological	40 (21.6%)	156 (12.7%)
Erectile	40 (27.3%)	181 (12.5%)

Table S4.2: Comorbidity Definitions

**Condition**	**ATC codes**	**ATC label**	**Exceptions/more specific definitions/notes**
Acid related disorders	A02A	ANTACIDS	
	A02B	DRUGS FOR ACID RELATED DISORDERS	Exclude where also taking M01A ANTIINFLAMMATORY AND ANTIRHEUMATIC PRODUCTS, NON-STEROIDS, or B01 (antithrombotic) drugs
Diabetes mellitus	A10	DRUGS USED IN DIABETES	Note this includes insulins and analogues, other blood glucose lowering drugs etc. It does not include cardiovascular prevention drugs We do not exclude metformin, although this is used to treat Polycystic ovary syndrome (PCOS).
Thromboembolic disease, atrial fibrillation or valvular heart disease	B01AA	Vitamin K antagonists	Do not include if only 4-level codes are available.
	B01AE	Direct thrombin inhibitors	Do not include if only 4-level codes are available.
	B01AF	Direct factor Xa inhibitors	Do not include if only 4-level codes are available.
Cardiovascular	C01	CARDIAC THERAPY DRUGS	

Table S4.2: Comorbidity Definitions

Condition	ATC codes	ATC label	Exceptions/more specific definitions/notes
diseases			
	C04	PERIPHERAL VASODILATORS	
	C02	ANTIHYPERTENSIVES	
	C07	beta-Adrenergic Blocking Agents	Except ?propranolol? or other ?C07AA Beta blocking agents, non-selective? non-selective beta-blockers where participant is also taking an N02C drug. Do not apply exclusion if these have only 4 or 5 characters (respectively).
	C08	CALCIUM CHANNEL BLOCKERS	
	C09	AGENTS ACTING ON THE RENIN-ANGIOTENSIN SYSTEM	
Urinary frequency and incontinence	G04BD	Drugs for urinary frequency and incontinence	If only-4-character code exclude.
Erectile dysfunction	G04BE	Drugs used in erectile dysfunction	If only-4-character code exclude.
Benign prostatic hypertrophy	G04C	DRUGS USED IN BENIGN PROSTATIC HYPERTROPHY	If only 3-character code exclude.
Glaucoma	S01E	ANTIGLAUCOMA PREPARATIONS AND MIOTICS	If only 3-character code define as eye disease.
Arthritis and arthralgia	M01A	ANTIINFLAMMATORY AND ANTIRHEUMATIC PRODUCTS, NON-STEROIDS	If only 3-character code define as arthritis and arthralgia, but accept some misclassification possible as indications for penicillamine include ?conditions associated with impaired copper metabolism? eg Wilson?s disease and gold is used to treat dermatological conditions.
	M01B	ANTIINFLAMMATORY/ANTIRHEUMATIC AGENTS IN COMBINATION	
	M02	TOPICAL PRODUCTS FOR JOINT AND MUSCULAR PAIN	
Osteoporosis (or risk factors for osteoporosis)	M05	DRUGS FOR TREATMENT OF BONE DISEASES	
Gout	M04	ANTIGOUT PREPARATIONS	Although allopurinol is being used for other indications, this is unlikely to be widespread.
Inflammatory arthropathies, inflammatory bowel disease, systemic lupus	A07EA	Corticosteroids acting locally	Where only 3-character codes are provided, define as any A07

Table S4.2: Comorbidity Definitions

Condition	ATC codes	ATC label	Exceptions/more specific definitions/notes
erythematosus and connective tissue diseases			
	A07EC	Aminosalicylic acid and similar agents	Where only 3- character codes are provided, define as any A07
	L04AB	Tumour necrosis factor alpha (TNF-) inhibitors	Where only 3- character codes are provided, define as any L04
	L04AA	Selective immunosuppressants	Where only 3- character codes are provided, define as any L04
	L04AX	Other immunosuppressants (includes methotrexate,azathioprine, and lefluonomide)	Where only 3- character codes are provided, define as any L04
	M01CB	Gold preparations	Do not define if only 3-character code is available. If only 4-character code available define, since only other agent is an obscure drug oxycinchophen
	M01CC	Penicillamine and similar agents	Do not define if only 3-character code is available. If only 4-character code available define, since only other agent is an obscure drug oxycinchophen
	D05	ANTIPSORIATICS	
Migraine	N02C	ANTIMIGRAINE PREPARATIONS	Do not define if only 3-character code is available
Pain	N02A	OPIOIDS	If only 3-character code available define as pain
	N02B	OTHER ANALGESICS AND ANTIPYRETICS	
Schizophrenia and delusional disorders	N05A	ANTIPSYCHOTICS	Prochlorperazine is included in this class. If individual drug data available exclude if prochlorperazine. If 5-character code available exclude ?N05AB Phenothiazines with piperazine structure?. If only 4-character code is available do not define.
Mood, neurotic and sleep disorders	N05B	ANXIOLYTICS	If only 3-character code available do not define.
	N05C	HYPNOTICS AND SEDATIVES	If only 3-character code available do not define
	N06A	ANTIDEPRESSANTS	Except amitriptyline. If drug term not available

Table S4.2: Comorbidity Definitions

Condition	ATC codes	ATC label	Exceptions/more specific definitions/notes
Epilepsy	N03	ANTIEPILEPTICS	Except where drug is pregabalin, gabapentin or valproic acid. If specific drug is not given, and if indication for drug is not stated proceed as follows. If only a 5-character code is provided exclude N03AX (which includes gabapentin and pregabalin) and N03AG (includes valproic acid). This will reduce sensitivity, but improve specificity. If only a 4-character code is provided do not attempt to define.
Parkinson?s disease and Parkinsonism	N04	ANTI-PARKINSON DRUGS	
Dementia	N06D	ANTI-DEMENTIA DRUGS	Do not define if only 3-character code is available
Chronic lower respiratory disease	R03	DRUGS FOR OBSTRUCTIVE AIRWAY DISEASES	
Thyroid disorders	H03	THYROID THERAPY DRUGS	
Skin diseases	D02A	EMOLLIENTS AND PROTECTIVES	
	D04	ANTIPRURITICS, INCL. ANTIHISTAMINES, ANESTHETICS, ETC.	
	D06	ANTIBIOTICS AND CHEMOTHERAPEUTICS FOR DERMATOLOGICAL USE	
	D07	CORTICOSTEROIDS, DERMATOLOGICAL PREPARATIONS	

4.3 Mapping ATC codes to READ codes

We mapped the ATC codes to Read codes (which are used in the SAIL data) off-line, using the NHS Business Authority mappings[6] and, for some more recent drugs such as novel antidiabetic drugs, by manually mapping between ATC and Read codes. The mapping was very good, even retaining information on route. For example the READ code for topical beclomethasone preparations mapped to different ATC codes to those for oral preparations.

4.4 Suppression of comorbid conditions

For all three data sources, comorbid diseases were excluded if these were considered to be identical to the main condition (Table S4.3). For example, a patient/participant with asthma could not be considered to have airways disease as a comorbidity. This inevitably involved clinical judgements, for example pain was not suppressed for rheumatoid arthritis as we considered that where inflammation was fully controlled, pain may not be present, while the patient would nonetheless have clear evidence of the disease. In contrast, given that osteoarthritis is essentially a degenerative process, we

concluded that the diagnosis was of doubtful validity in the absence of at least some pain. These exclusions were nonetheless applied identically across all three cohorts.

Table S4.3: Indication/condition pairs where the condition is not considered a comorbidity

**Indication**	**Not defined as comorbid**
Alzheimer's Disease	Dementia
Alzheimer's Disease	Schizophrenia and delusional disorders
Ankylosing Spondylitis	Inflammatory arthropathies, inflammatory bowel disease, systemic lupus erythematosus and connective tissue diseases
Ankylosing Spondylitis	Arthritis and arthralgia
Asthma	Chronic lower respiratory disease
Atrial Fibrillation, Stroke	Cardiovascular diseases
Atrial Fibrillation, Stroke	Thromboembolic disease, atrial fibrillation or valvular heart disease
Benign Prostatic Hyperplasia	Benign prostatic hypertrophy
Benign Prostatic Hyperplasia	Urinary frequency and incontinence
Chronic Idiopathic Urticaria (Ciu)	Inflammatory arthropathies, inflammatory bowel disease, systemic lupus erythematosus and connective tissue diseases
Diabetes Mellitus	Diabetes mellitus
Diabetes Mellitus, Type 2	Diabetes mellitus
Diabetes Mellitus, Type 2; Hypertension	Diabetes mellitus
Diabetes Mellitus, Type 2; Hypertension	Hypertension
Diabetes Mellitus, Type 2; Renal Insufficiency	Diabetes mellitus
Diabetes Mellitus, Type 2; Renal Insufficiency	Renal
Erectile Dysfunction, Benign Prostatic Hyperplasia	Erectile dysfunction
Erectile Dysfunction, Benign Prostatic Hyperplasia	Benign prostatic hypertrophy
Erectile Dysfunction, Benign Prostatic Hyperplasia	Urinary frequency and incontinence
Hypertension, Pulmonary	Thromboembolic disease, atrial fibrillation or valvular heart disease
Osteoporosis	Osteoporosis (or risk factors for osteoporosis)
Osteoporosis, Male	Osteoporosis (or risk factors for osteoporosis)
Osteoporosis; Hip Fracture	Osteoporosis (or risk factors for osteoporosis)
Parkinson Disease	Parkinsons disease and Parkinsonism
Psoriasis	Inflammatory arthropathies, inflammatory bowel disease, systemic lupus erythematosus and connective tissue diseases
Pulmonary Disease, Chronic Obstructive	Chronic lower respiratory disease
Rheumatoid Arthritis	Inflammatory arthropathies, inflammatory bowel disease, systemic lupus erythematosus and connective tissue diseases
Rheumatoid Arthritis	Arthritis and arthralgia
Systemic Lupus Erythematosus	Inflammatory arthropathies, inflammatory bowel disease, systemic lupus erythematosus and connective tissue diseases
Thromboembolism	Thromboembolic disease, atrial fibrillation or valvular heart disease
Thromboprophylaxis	Thromboembolic disease, atrial fibrillation or valvular heart disease
Type 2 Diabetes Mellitus	Diabetes mellitus
Ulcerative Colitis; Crohn's Disease	Inflammatory arthropathies, inflammatory bowel disease,

Table S4.3: Indication/condition pairs where the condition is not considered a comorbidity

Indication	Not defined as comorbid
	systemic lupus erythematosus and connective tissue diseases
Venous Thromboembolism	Thromboembolic disease, atrial fibrillation or valvular heart disease
Crohn’s Disease	Inflammatory arthropathies, inflammatory bowel disease, systemic lupus erythematosus and connective tissue diseases
Ulcerative Colitis	Inflammatory arthropathies, inflammatory bowel disease, systemic lupus erythematosus and connective tissue diseases
Psoriatic Arthritis	Inflammatory arthropathies, inflammatory bowel disease, systemic lupus erythematosus and connective tissue diseases
Psoriatic Arthritis	Arthritis and arthralgia
Migraine	Pain
Migraine	Migraine
Osteoarthritis	Pain
Osteoarthritis	Arthritis and arthralgia
Restless Legs Syndrome	Parkinsons disease and Parkinsonism
Hypertension	Hypertension
Hypertension	Cardiovascular diseases
Diabetic Nephropathies	Diabetes mellitus
Diabetic Nephropathies	Renal
Arthroplasty, Replacement, Knee; Thromboembolism	Pain
Thromboembolism; Arthroplasty, Replacement, Hip	Pain
Diabetes Mellitus, Type 2; Hyperglycemia	Diabetes mellitus
Rhinitis, Allergic, Perennial	Chronic lower respiratory disease

4.5 Comorbidity counts

For the comorbidity count calculation, these concomitant medication definition pairs were also collapsed into a single condition (Table S4.4).

Table S4.4: Conditions as recorded on CSDR site

Definition 1	Definition 2
Pain	Migraine
Pain	Rheumatologic conditions

4.6 R code for comorbid disease definitions

Comorbid diseases were implemented, consistently across all datasts using the following R code.

```
library(tidyverse)
library(stringr)

# read in conmed data
conmed <- readRDS("Data/conmed_randomised_cleaned_no_contradictions.Rds") %>%
  filter(!is.na(drug_class)) %>%
  distinct()
rxnorm_bnf <- read_csv("Supporting/bnf_rxnorm_atc_codes.zip")
rxnorm_bnf <- rxnorm_bnf %>%
  filter(!is.na(str))

###### Functions
PrintDrugChoices <- function(incld, excld = FALSE, mydf = conmed){
```

```
  mydf2 <- mydf %>%
    mutate(incld = incld, excld = excld) %>%
    group_by(trial, id) %>%
    mutate(excld = any(excld)) %>%
    ungroup() %>%
    filter(incld & !excld) %>%
    group_by(term, route_classify, atc_code) %>%
    count(sort = TRUE)
  mydf2
}

ApplyMedCriteria <- function(incld, excld = FALSE, print = TRUE, mydf = conmed){
  PrintDrugChoices(incld, excld, mydf) %>%  head(19) %>%  print()
  mydf2 <- mydf %>%
    mutate(incld = incld, excld = excld) %>%
    group_by(trial, id) %>%
    summarise(present = any(incld) & !any(excld))
  print( paste0(round(100* mean(mydf2$present), 1), "%"))
  mydf2
}

## Rename variables in dataset so do not ahve to rename code
conmed <- conmed %>%
  rename(atc_code = drug_class,
         trial = trial_id_trunc,
         route_classify = route)

############ Define comorbidities based on concomitant medicaitons
## First remove aspirin from all analyses as is used widely as prophylaxis
## Similarly remove amitriptyline as most frequently used for PAIN not for depression,
anxiety etc
## Updated this code to be more specific, particularly for aspirin

DropDrugs <- function(mystring = "acetylsalicylic acid|aspirin") {
  x <- rxnorm_bnf %>%
    filter(str_detect(str, mystring))
  x <- rxnorm_bnf %>%
    filter(code %in% x$code | str %in% x$str) %>%
    transmute(term_lower = str, atc_code = str_sub(code, 1, 5))
  x
}

aspirin_codes <- c("A01DA05", "B01AC06", "N02BA01")
aspirin <- rxnorm_bnf %>%
  filter(code %in% aspirin_codes) %>%
  mutate(term_lower = str_to_lower(str)) %>%
  distinct(term_lower, code)

amitriptyline <- DropDrugs("amitriptyl")
pre_gab_val <- DropDrugs("gabapentin|pregabalin|valrpoate|valproic acid")

conmed <- conmed %>%
  mutate(term_lower = str_to_lower(term) %>%
           str_replace("/[0-9]{8,8}/", "") %>%
           str_trim()) %>%
  anti_join(aspirin %>%  select(term_lower))

conmed <- conmed %>%
  anti_join(amitriptyline %>%  select(term_lower))

conmed <- conmed %>%
  anti_join(pre_gab_val %>%  select(term_lower))

## Antacids
# Note only antacid codes are A02A, A02B, A02X and the last is an empty category
antacids_included <- conmed$atc_code  %>% str_sub(1, 4) %in% c("A02A", "A02B") |
  conmed$atc_code %>% str_sub(1, 3) == "A02"
```

```r
antacids_excluded <- (conmed$atc_code  %>% str_sub(1, 4) %in% "M01A") |
  (conmed$atc_code  %>% str_sub(1, 3) %in% "B01")
conmed_ant <- ApplyMedCriteria(antacids_included, antacids_excluded)

## Diabetes
diabetes_included <- conmed$atc_code  %>% str_sub(1, 3) %in% c("A10")
conmed_diab <- ApplyMedCriteria(diabetes_included)

## thromboembolic
tbe_included <- str_sub(conmed$atc_code, 1, 5) %in% c("B01AA", "B01AE", "B01AF")
conmed_tbe <- ApplyMedCriteria(tbe_included)

## Cardiovascular (CV)
cv_codes <- paste0("C0", c(1,2,4,7,8,9))
cv_included <- str_sub(conmed$atc_code, 1, 3) %in% cv_codes
cv_excluded <- str_sub(conmed$atc_code, 1, 4) == "N02C" &
  str_sub(conmed$atc_code, 1, 5) == "C07AA"
# Where has only 4 digits cannot exclude beta-blockers, where only has 3 cannot exclude
antimigraine
conmed_cv <- ApplyMedCriteria(cv_included, cv_excluded)

## Urinary incontinence
ur_included <- str_sub(conmed$atc_code, 1, 5) == "G04BD"
conmed_ur <- ApplyMedCriteria(ur_included)

## Erectile dysfunction (ED)
ed_included <- str_sub(conmed$atc_code, 1, 5) == "G04BE"
conmed_ed <- ApplyMedCriteria(ed_included)

## Urinary incontinence or ED
## If only 4 character code is available define as urinary incontinence or ED
ur_ed_included <- str_sub(conmed$atc_code, 1, 4) == "G04B" & str_length(conmed$atc_code
) == 4
conmed_ur_ed <- ApplyMedCriteria(ur_ed_included)

## Benign prostatic hypertrophy (BPH)
bph_included <- str_sub(conmed$atc_code, 1, 4) == "G04C"
conmed_bph <- ApplyMedCriteria(bph_included)

## Urinary incontinence or ED or BPH, these are tiny proportions, probably ignore
## If only 3 character code is available define as urinary incontinence or ED or BPH
ur_ed_bph_included <- str_sub(conmed$atc_code, 1, 3) == "G04" & str_length(conmed$atc_c
ode) == 3
conmed_ur_ed_bph <- ApplyMedCriteria(ur_ed_bph_included)

## Glaucoma
# drop isosorbide even though classified as an eye drug as is not a contemporary drug f
or glaucoma
glaucoma <- str_sub(conmed$atc_code, 1, 4) == "S01E" &
  (is.na(conmed$term) | conmed$term != "ISOSORBIDE")
conmed_gl <- ApplyMedCriteria(glaucoma)

## Arthritis and arthralgia
# Exclude FOLIC ACID from this as is very common and (despite class here)
# is not an M01AX code in WHO ATC
art <- ((str_sub(conmed$atc_code, 1, 4) %in% c("M01A", "M01B") |
          str_sub(conmed$atc_code, 1, 3) == "M02")) &
        (is.na(conmed$term) |
          conmed$term != "FOLIC ACID")
conmed_art <- ApplyMedCriteria(art)

## Osteoporosis
ost <- str_sub(conmed$atc_code, 1, 3) == "M05"
conmed_ost <- ApplyMedCriteria(ost)

## Gout
gou <- str_sub(conmed$atc_code, 1, 3) == "M04"
```

```r
conmed_gou <- ApplyMedCriteria(gou)

## Inflammatory arthropathies, psoriasis, inflammatory bowel disease or conective tissu
e diseases
inf <- str_sub(conmed$atc_code, 1,5) %in% c("A07EA", "A07EC", "L04AB", "L04AA",
                                            "L04AX", "M01CB", "M01CC")
conmed_inf <- ApplyMedCriteria(inf)

inf3 <- (!inf) & (str_length(conmed$atc_code  == 3) &
                    str_sub(conmed$atc_code, 1, 3) %in% c("A07", "L04", "D05"))
inf4 <- (!inf) & (str_length(conmed$atc_code == 4) &
                    str_sub(conmed$atc_code, 1, 4) %in% c("M01C"))
conmed_inf4 <- ApplyMedCriteria(inf4|inf3)

## Migraine
mig <- str_sub(conmed$atc_code, 1, 4) == "N02C"
conmed_mig <- ApplyMedCriteria(mig)

## Pain
pai <- str_sub(conmed$atc_code, 1, 4) %in% c("N02A", "N02B") &
 (!str_sub(conmed$atc_code, 1, 5) == "N02BA")
# Already excluded aspirin above
conmed_pai <- ApplyMedCriteria(pai)

pai3 <- !pai & (str_length(conmed$atc_code ==3) &
                  str_sub(conmed$atc_code, 1, 3) == "N02")
#already excluded aspirin
conmed_pai3 <- ApplyMedCriteria(pai3)

## schizophrenia and delusional disorders
sch_include <- str_sub(conmed$atc_code, 1, 4) == "N05A"
sch_exclude <- str_sub(conmed$atc_code, 1, 5) == "N05AB"
conmed_sch <- ApplyMedCriteria(sch_include, sch_exclude)

#  Anxiety and mood disorders
anx <- str_sub(conmed$atc_code, 1, 4) %in% c("N05B", "N05A", "N06A")
# already excluded amitriptyline
conmed_anx <- ApplyMedCriteria(anx)

##  Epilepsy
epi <- str_sub(conmed$atc_code, 1, 3) == "N03"
# Already excluded GABAPENTIN, PREGABALIN AND VALPROATE terms
# WIll also exclude 5-digit code when text is missing
pre_gab_val5 <- str_length(conmed$atc_code ==5) & is.na(conmed$term) & conmed$atc_code
== "N03AX"
epi_excld <-  pre_gab_val5
conmed_epi <- ApplyMedCriteria(epi, epi_excld)

## Parkinson'd disease and Parkinsonism
pd <- str_sub(conmed$atc_code, 1, 3) == "N04"
conmed_pd <- ApplyMedCriteria(pd)

## Dementia
dem <- str_sub(conmed$atc_code, 1, 4) == "N06D"
conmed_dem <- ApplyMedCriteria(dem)

## Chronic lower respiratory disease (predominantly asthma and/or COPD)
resp <- str_sub(conmed$atc_code, 1, 3) == "R03"
conmed_resp <- ApplyMedCriteria(resp)

## Thyroid disease (hyper and hypothroidism incldued)
thy <- str_sub(conmed$atc_code, 1, 3) == "H03"
conmed_thy <- ApplyMedCriteria(thy)

## Skin diseases
skn <- str_sub(conmed$atc_code, 1, 4) == "D02A" |
  str_sub(conmed$atc_code, 1, 3) %in% c("D04", "D06", "D07")
```

```r
conmed_skn <- ApplyMedCriteria(skn)

## Combine all conditions into a single dataset
a <- list("conmed_ant", "conmed_anx", "conmed_art", "conmed_bph", "conmed_cv",
          "conmed_dem", "conmed_diab", "conmed_ed", "conmed_epi", "conmed_gl",
          "conmed_gou", "conmed_inf", "conmed_inf4", "conmed_mig", "conmed_ost",
          "conmed_pai", "conmed_pai3", "conmed_pd", "conmed_resp", "conmed_sch",
          "conmed_tbe", "conmed_thy", "conmed_ur", "conmed_ur_ed", "conmed_ur_ed_bph",
          "conmed_skn"
)
conmed_all <- map(a, get)
conmed %>% distinct(company, trial, id) %>% nrow()
names(conmed_all) <- str_replace(a, "conmed_", "")
conmed_all <- map2(conmed_all, names(conmed_all), ~
                     set_names(.x, c("trial", "id", .y)))
conmed_all <- reduce(conmed_all, inner_join)

## Create more informative labels for conditions
b <- list("ant", "anx", "art", "bph", "cv", "dem", "diab", "ed", "epi",
          "gl", "gou", "inf", "inf4", "mig", "ost", "pai", "pai3", "pd",
          "resp", "sch", "tbe", "thy", "ur", "ur_ed", "ur_ed_bph", "skn")
b_lbl <- c('antacids', 'anxiety', 'arthritis', 'prostate', 'CV',
           'dementia', 'diabetes', 'erectile', 'epilepsy', 'glaucoma',
           'gout', 'inflammatory', 'inflammatory4', 'migraine', 'osteoporosis',
           'pain', 'pain3', 'parkinsons', 'asthma_COPD', 'schizophrenia',
           'thromboembolic', 'thyroid', 'urological', 'urological_or_ed', 'urological_o
r_ed_or_bph',
           'skin')
smrs_all <- map(b, function(x) tapply(conmed_all[[x]], conmed_all$trial, mean))
names(smrs_all) <- b_lbl

b_lkp <- b_lbl
names(b_lkp) <- b
other_names <- setdiff(names(conmed_all), names(b_lkp))
names(other_names) <- other_names
b_lkp <- c(other_names, b_lkp)
names(conmed_all) <- b_lkp[names(conmed_all)]

conmed_all <- trial_indic_drug %>%
  select(nct_id, medicine, condition, trial) %>%
  inner_join(conmed_all)

## Collapse additional conditions
## Clearly very uncommon to have urological 3-level codes, so drop for simplicity
conmed_all <- conmed_all %>%
  mutate(pain = pain|pain3,
         inflammatory = inflammatory|inflammatory4) %>%
  select(-pain3, -inflammatory4, -urological_or_ed,
         -urological_or_ed_or_bph)

## set condition to null where it corresponds to the indication condition
## Rename and update now adding in YODA trials
## Solely YODA condition terms are "Crohn's disease", "Ulcerative colitis", "Psoriatic
arthritis",
# and "Migraine" the others appear in CSDR

condition_match <- c("Alzheimer's Disease", "ankylosing spondylitis", "Asthma",
                     "Atrial Fibrillation, Stroke", "Benign Prostatic Hyperplasia",
                     "Chronic Idiopathic Urticaria (CIU)", "Diabetes Mellitus",
                     "Diabetes Mellitus, Type 2",
                     "Diabetes Mellitus, Type 2; Hypertension",
                     "Diabetes Mellitus, Type 2; Renal Insufficiency",
                     "Erectile Dysfunction, Benign Prostatic Hyperplasia",
                     "Hypertension, Pulmonary",
                     "Osteoporosis", "Osteoporosis, Male", "Osteoporosis; Hip Fracture"
,
```

```
                      "Parkinson Disease", "Psoriasis", "Pulmonary Disease, Chronic Obst
ructive",
                      "rheumatoid arthritis", "Systemic Lupus Erythematosus", "Thromboem
bolism",
                      "Thromboprophylaxis", "Type 2 Diabetes Mellitus",
                      "Ulcerative Colitis; Crohn's Disease",
                      "Venous Thromboembolism",
                      "Crohn's disease",
                      "Ulcerative colitis",
                      "Psoriatic arthritis",
                      "Migraine",
                      "Osteoarthritis",
                      "Restless Legs Syndrome",
                      "Hypertension",
                      "Diabetic Nephropathies",
                      "Arthroplasty, Replacement, Knee; Thromboembolism",
                      "Thromboembolism; Arthroplasty, Replacement, Hip",
                      "Diabetes Mellitus, Type 2;  Hyperglycemia",
                      "Rhinitis, Allergic, Perennial")
conmed_all <- conmed_all %>%
  mutate(condition = case_when(
    condition == "Rheumatoid arthritis" ~ "rheumatoid arthritis",
    condition == "Ankylosing spondylitis" ~ "ankylosing spondylitis",
    condition %in%
      c("Type 2 diabetes", "Diabetes Mellitus, Type 2;  Hyperglycemia") ~ "Diabetes Mel
litus, Type 2",
    condition == "Alzheimer's" ~ "Alzheimer's Disease",
    TRUE ~ condition
  ))

names(condition_match) <- condition_match
condition_match <- as.list(condition_match)
condition_match$`Alzheimer's Disease` <- c("dementia", "schizophrenia")
condition_match$Asthma <- "asthma_COPD"
condition_match$`Atrial Fibrillation, Stroke` <- c("CV", "thromboembolic")
condition_match$`Benign Prostatic Hyperplasia` <- c("prostate", "urological")
condition_match$`Chronic Idiopathic Urticaria (CIU)`<- "inflammatory"
condition_match$`Diabetes Mellitus` <- "diabetes"
condition_match$`Diabetes Mellitus, Type 2` <- "diabetes"
condition_match$`Diabetes Mellitus, Type 2; Hypertension` <- c("diabetes", "hypertensio
n")
condition_match$`Diabetes Mellitus, Type 2; Renal Insufficiency` <- c("diabetes", "rena
l")
condition_match$`Erectile Dysfunction, Benign Prostatic Hyperplasia` <- c("erectile", "
prostate", "urological")
condition_match$`Hypertension, Pulmonary` <- "thromboembolic"
condition_match[c("Osteoporosis", "Osteoporosis, Male", "Osteoporosis; Hip Fracture")]
<- "osteoporosis"
condition_match$`Parkinson Disease` <- "parkinsons"
condition_match$Psoriasis <- c("inflammatory", "skin")
condition_match$`Pulmonary Disease, Chronic Obstructive` <- "asthma_COPD"
condition_match[c("rheumatoid arthritis", "ankylosing spondylitis",
                  "Psoriatic arthritis")] <- map(
                    condition_match[c("rheumatoid arthritis", "ankylosing spondylitis",
                                      "Psoriatic arthritis")],
               function(x) c("inflammatory", "arthritis"))
condition_match$`Systemic Lupus Erythematosus`<- "inflammatory"
condition_match$Thromboembolism <- "thromboembolic"
condition_match$Thromboprophylaxis <- "thromboembolic"
condition_match$`Type 2 Diabetes Mellitus` <- "diabetes"
condition_match[c("Ulcerative Colitis; Crohn's Disease",
                "Crohn's disease",
                "Ulcerative colitis")] <- "inflammatory"
condition_match$`Venous Thromboembolism` <- "thrombembolic"
condition_match$Migraine <- c("pain", "migraine")
condition_match$Osteoarthritis <- c("pain", "arthritis")
condition_match$`Restless Legs Syndrome` <- c("parkinsons")
```

```r
condition_match$Hypertension <- c("hypertension", "CV")
condition_match$`Diabetic Nephropathies` <- c("diabetes", "renal")
condition_match$`Arthroplasty, Replacement, Knee; Thromboembolism` <- c("pain")
condition_match$`Thromboembolism; Arthroplasty, Replacement, Hip` <- c("pain")
condition_match$`Diabetes Mellitus, Type 2;  Hyperglycemia` <- "diabetes"
condition_match$`Rhinitis, Allergic, Perennial` <- "asthma_COPD"

saveRDS(condition_match, "Scratch_data/index_conditions_matching_comorbidities.Rds")

## For sail create as dataframe
# condition_match_df <- stack(condition_match)
# names(condition_match_df) <- c("conmed", "index")
# write_csv(condition_match_df, "Outputs/index_comorbid_suppress.csv")

## Apply exclusion by setting each concomitant disease to FALSE if is also the indicati
on
conmed_all_final <- conmed_all
conmed_all_final2 <- conmed_all_final
for (condition_slct in names(condition_match)) {
  print(paste0(condition_slct, " - ",condition_match[[condition_slct]]))
  conmed_all_final2[conmed_all_final$condition %in% condition_slct,
                    names(conmed_all_final2) %in% condition_match[[condition_slct]]
                    ] <- FALSE
}
conmed_all_final <- conmed_all_final2
rm(conmed_all_final2)
saveRDS(conmed_all_final, "Data/comorbidity_based_on_conmeds.Rds")

## Count conditions after have excluded index disease
# Avoid double-counting of pain
## Drop skin as concerns about completeness of recording
conmed_all_final_count <- conmed_all_final %>%
  mutate(skin = FALSE,
         pain = if_else(migraine|arthritis, FALSE, pain),
         arthritis = if_else(inflammatory, FALSE, arthritis))
# Create a disease count variable for each participant
conmed_all_final_count <- conmed_all_final_count %>%
  mutate(disease_count = NA)
conmed_all_final_count$disease_count <- rowSums(conmed_all_final_count %>% ungroup() %>
%
                                                  select(antacids:urological))
a <- table(conmed_all_final_count$disease_count)
round(100*cumsum(rev(a))/nrow(conmed_all_final_count),2)
# Aggregate the disease count for each trial
conmed_all_final_count_agg <- conmed_all_final_count %>%
  group_by(nct_id, disease_count) %>%
  summarise(x = length(disease_count)) %>%
  group_by(nct_id) %>%
  mutate(n = sum(x)) %>%
  ungroup() %>%
  mutate(prop = round(100*x/n,1))

# Spread this to wide for easier examination
conmed_all_final_count_agg_wide <- conmed_all_final_count_agg %>%
  select(nct_id, n, disease_count, prop) %>%
  spread(disease_count, prop, fill = 0)

## Summarise the proportion of participants with each comorbidity within each trial
conmed_all_trial_lvl <- conmed_all_final %>%
  select(-id) %>%
  group_by(trial, nct_id, medicine, condition) %>%
  summarise_all(function(x) round(100*mean(x),1))

write_csv(conmed_all_trial_lvl,
          "Outputs/Trial level counts of people with each disease based on concomittant
medicines.csv")
write_csv(conmed_all_final_count_agg_wide,
```

```
          "Outputs/Trial level counts of people with disease counts based on concomitta
nt medicines.csv")
```
