# Supplementary material for: Representation of people with comorbidity and multimorbidity in clinical trials of novel drug therapies: an individual-level participant data analysis
Source: BMC Med. 2019 Nov 12;17:201. doi: 10.1186/s12916-019-1427-1 (PMC6849229; doi:10.1186/s12916-019-1427-1)
Supplement: Supplementary file 5 — Additional file 5. More-detailed-statistical-analysis.pdf: Model description and code required for analyses. Detailed description of statistical methods. [file 12916_2019_1427_MOESM5_ESM.pdf]

5 More detailed statistical analysis

We compared comorbidity counts in the primary care (SAIL) and trial datasets having standardised the SAIL data to the number of trial participants with each condition in each age and sex band.

5.1 Rationale and overview of modelling

We chose to use standardisation because the individual level data, and hence age-sex specific comorbidity counts, are held in separate data “safe havens” (two for the trial data and a further safe haven for the primary care data) which would have made it difficult to fit a single model. We then used a simulation-based approach to model the uncertainty. This allowed us to propagate the uncertainty from the comorbidity counts, from both the primary care and trial data, through the standardisation calculations. This informed the choice to sample from Dirichlet distributions for the primary care data and individual trials, and to fit a Bayesian model using Markov Chain Monte Carlo (MCMC) to estimate the overall trial comorbidity distribution (for each index condition).

Briefly, for each condition, we applied the proportions with each comorbidity count for each age and sex specific stratum from SAIL to the age and sex distribution from the trials data to obtain the expected comorbidity counts for each stratum, then summed this across strata. This is essentially direct standardisation (albeit with proportion rather than rate data as is more common).

The following describes the individual steps of this modelling. Steps 1 and 7 were performed in the trial repository, and Steps 10 to 13 were performed within the SAIL safe haven, with the remaining steps being performed outside of the safe haven using aggregated/summary data.

5.2 Detailed description of statistical analysis

Step 1 - Obtain counts of the number of participants with each comorbidity count from the trial data

For each trial, we summarised the number of participants with each comorbidity count. Table S10.1 shows these data.

Step 2 - Summarise the number of participants with each comorbidity count for each trial, using the assumption that these counts are Poisson distributed, and test whether this is a reasonable assumption

Outside the trial repository, for each trial, we calculated the mean comorbidity count (λ) across participants using the aggregated data as $\sum_i (proportion_i \times count_i)$ where i indexed the comorbidity counts from 0 to the integer for the highest observed count (which was 10 for the trial data). We calculated the mean count in order to have a single parameter for describing the distribution of comorbidity counts for each trial. This then made subsequent modelling more straightforward, but did require the assumption that the distribution of participants with different comorbidity counts followed a Poisson distribution.

To confirm that this assumption was reasonable, we compared the observed proportion of participants with each comorbidity count (from Step 1) to the expected proportion of participants with each comorbidity count under a Poisson distribution. The latter was derived from the probability mass function of the Poisson distribution $\frac{\lambda^k e^{-\lambda}}{k!}$, where λ was the mean count and k corresponded to comorbidity counts ranging from 0 to 12. For a random sample of 25 trials, Figure S5.1 shows these observed and expected counts. We found that the distribution of participants with each comorbidity count within the trials did closely follow a Poisson distribution.

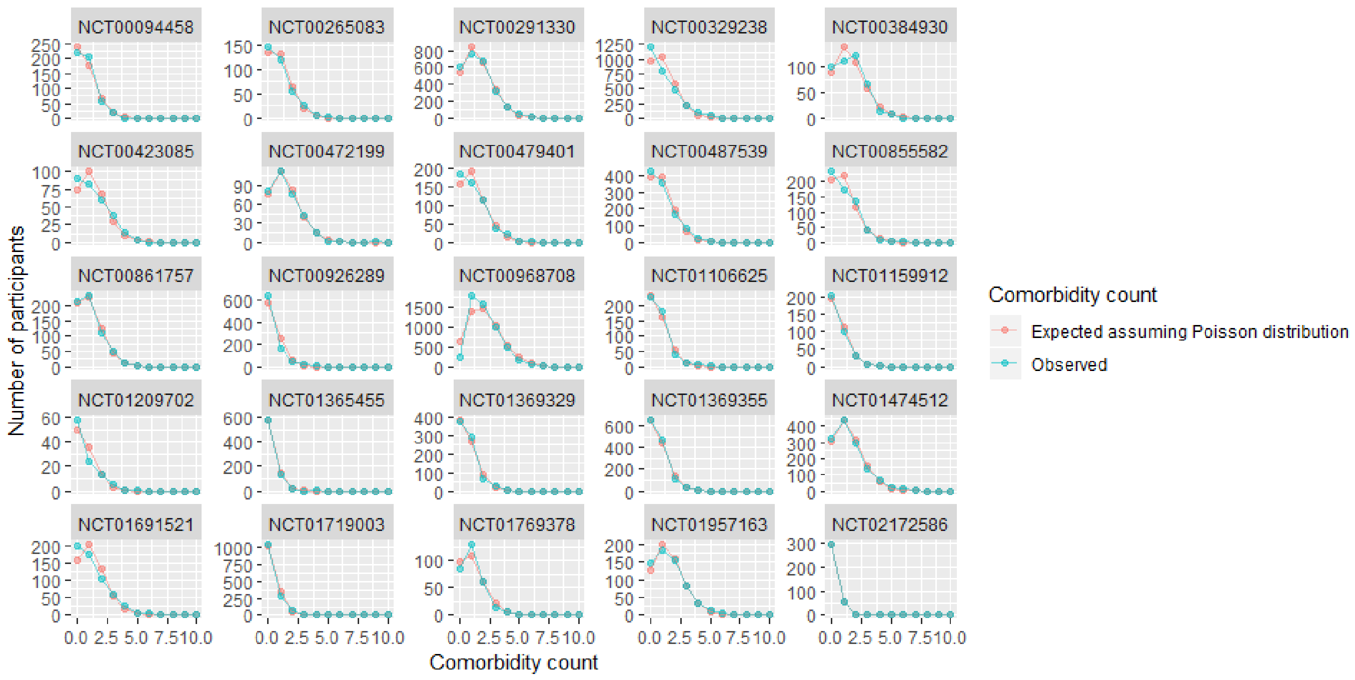

Figure S5.1: Distribution of comorbidity counts within each trial

Step 3 - Estimate mean comorbidity counts at the condition-level for conditions where there was more than one trials

Where there were multiple trials within a condition, we fitted a random effects Poisson regression model to obtain the expected comorbidity count for that condition. The modelling was performed in the statistical package JAGS. The JAGS code (which is similar to WINBUGS code) for this model is shown below.

5.2.0.1 Poisson model, random intercept, single between-trial (within indication) variance

```
model{
  for(i in 1:i_max){
    ## Trial level
    for(j in 1:j_max){
      ys[i,j] ~ dpois(lambda[i,j])
      log(lambda[i,j]) <- intercept[i,j] + log(ns[i,j])
      intercept[i,j] ~ dnorm(mu_trial[i], prec_trial)
  } # end trial
  } # end condition
    ## Prior for intercept at level of condition,  independent for each condition
    for (i in 1:i_max){
      mu_trial[i] ~ dnorm(0, 0.1)
    }
    ## High-level priors
    ## Single common prior for between trial variation
    prec_trial <- 1/sd_trial^2
    sd_trial ~ dnorm(0, 1)T(0,)
} # End of model
```

i indicates each condition and j each trial. We included a parameter for the expectation for each trial (intercept[i,j]), summarised via a higher-level parameter for each condition (mu_trial[i]). This meant the model is similar to a "random effects" meta-analysis. The condition-level expectation was estimated separately for each condition. However, as some conditions had few trials (sometimes only two trials), and it is known to be difficult to estimate between-trial variances in meta-analyses, we opted to model a single between-trial variance, within each condition, for all conditions.

Step 4 - Convert mean comorbidity counts to the proportion of participants with each comorbidity count, at the condition-level

As the model in Step 3 was a Poisson model, the mean comorbidity count at the level of each condition was calculated by exponentiating the parameter mu_trial. We used the resulting value (λ) to obtain the proportion of participants with each comorbidity count using the Poisson probability mass function $\frac{\lambda^k e^{-\lambda}}{k!}$. As before, k corresponds to a range of comorbidity counts for the integers 0 through 12. We assumed that the condition-level comorbidity counts were, like the trial counts, Poisson distributed.

We performed this calculation at the condition-level for 1000 samples obtained from the posterior distribution using the model described in Step 3. This allowed us to create a 1000 x 13 matrix, where each column corresponded to the proportion of participants with a specific comorbidity count (from 0 to 12) with each row corresponding to these proportions for different mean comorbidity count samples from the model in Step 3.

Step 5 - Directly sample from the trial-level summary data to obtain uncertainty estimates for the proportion of participants with each comorbidity count

To obtain uncertainty estimates for comorbidity counts at the level of individual trials, and for conditions where there was only one trial (eg osteoarthritis), we did not fit a regression model. Instead, we sampled directly from a Dirichlet distribution which is conjugate to the multinomial likelihood. We obtained the samples from the Dirichlet distribution in R as `MCMCpack::rdirichlet(1000, prior + number_of_participants_with_each_count)`. The prior for the proportion for each count was 1/13. This is analogous to the non-informative Beta (0.5, 0.5) "Jeffrey's" prior for the binomial likelihood.

As for the multi-trial/condition-level data this produced a 1000 x 13 matrix, with each column showing the proportion of participants with a specific comorbidity count (from 0 to 12) with each row corresponding to a different sample from the Dirichlet distribution.

Step 6 - Combine trial-level and condition-level samples

We then combined the matrices from Steps 5 and 6 to obtain samples summarising the uncertainty distribution for the proportion of participants with each comorbidity count. The resultant data was then uploaded to the primary care SAIL repository.

Step 7 - Summarise age-distribution for each sex for trial-level data using empirical mean, standard deviation and lower and upper bounds

Within the trial repository, we obtained estimates of the mean and standard deviation for age, along with upper and lower bounds based on the trial eligibility criteria. These were then exported from the trials repository.

Step 8 - Summarise age-distribution for each sex using truncated normal distributions

We summarised the sex-specific age distributions using truncated normal distributions. The truncated normal distribution was chosen over the normal distribution to reflect the fact that many trials impose age cut-offs for trial participation (allowing for the fact that age can be sharply cut-off at these levels). We used the truncated normal distribution to summarise the age data in preference to binning the data primarily to avoid concerns about disclosiveness (eg if only 5 patients were aged under 40 in a trial, this could in theory be disclosive and the data may not have been released by the trials repository).

We obtained the parameters for the truncated normal distribution using functions which output the expectation and variance from truncated normal distributions given the central tendency, spread, lower and upper bounds. We obtained estimates for these parameters by ranging over a two-dimensional grid for the central tendency and spread (since the lower and upper bounds were known these were fixed) selecting the parameters which best corresponded to the observed mean and standard deviation. Within the trial repository we then compared these truncated normal distributions to the observed distributions, finding that these closely corresponded to one another. The plots, which could also arguably be disclosive, are held within the secure trial repositories.

Step 9 - Obtain proportion of trial-participants aged 0 to 100, using the cumulative distribution function for the truncated normal

For each trial, for each sex, we obtain the proportion of trial participants within one-year each age bands using the cumulative distribution function. This can be obtained in R using the msm::ptnorm function. `msm::ptnorm(seq(0, 100, 1), expectation, standard_deviation, lower_bound, upper_bound)` . To obtain the distribution for each condition, we obtained a weighted mean (weighting by the number of participants in each trial) of the proportions in each trial. The resultant data was then uploaded to the primary care SAIL repository.

We did not sample repeatedly from a probability distribution for the age-sex distributions within trials, but rather treated this as fixed.

Step 10 - Obtain counts of the number of patients with each comorbidity count from the SAIL data

Within the SAIL safe haven, for each condition and each sex within 5-year age-bands, we summarised the number of patients with each comorbidity count.

Step 11 - Standardise primary care proportions of patients with each comorbidity count to the age-sex distributions obtained from trial data

For each condition, age-band and sex we obtained the the proportion with each comorbidity count from the summary data obtained in Step 10. We then collapsed the one-year age-bands for the trial data into five-year bands. We then multiplied the proportion with each comorbidity count by the proportion of trial participants in the corresponding age-sex-condition band. We then summed across age-sex bands within each condition to obtain the standardised proportion with each comorbidity count. One of the ways that we checked our calculations was by performing this standardisation for the original age-sex distribution within the community sample. In so doing we recovered the original unstandardised results. We then exported the proportion with each count Table S10.2).

The proportions with each comorbidity count for trials and community data were then compared graphically (Figure 3 in the main manuscript).

Step 12 - Sample from the condition-level summary data to obtain uncertainty estimates for the proportion of patients in the community with each comorbidity count

In the same manner as Step 5, within the SAIL safe haven, we obtained samples for the proportion of patients in the community with each comorbidity count by sampling from a Dirichlet distribution using the summary counts from Step 10 as the input parameters. This resulted in a 1000 x 13 matrix with each column corresponding to the proportion of patients with that comorbidity count (from 0 to 12) with each row being a sample from the Dirichlet distribution. We combined all the matrices for each age (in 5-year bands) and sex band within each condition.

We then repeated Step 11, with each of the 1000 samples in place of the empirical proportions, estimating the standardised proportion of community patients with each comorbidity count for each sample.

Step 13 Obtain summary statistics

Within the SAIL safe haven, for each sample obtained in Step 12, we obtained mean standardised comorbidity counts for each condition as $\sum_i (proportion_i \times count_i)$ where i is each comorbidity count from 0 to 12. We then compared these samples to the trial-derived samples obtained in Step 6 to obtain a number of summary statistics.

First we compared the mean count for both trial and community populations (on the ratio scale). In additional analyses we compared the proportion with 2 or more comorbidities (in addition to the index disease). Finally, we reported the proportion of primary care patients with a comorbidity count >= the median comorbidity count for the overall trial estimate. Uncertainty intervals were obtained by calculating these statistics using 1,000 samples from each distribution, and presenting the values for the 2.5th and 97.5th percentiles. We chose 2 or more comorbidities to attempt to identify a high-comorbidity group.

5.3 Sensitivity analyses and modelling choices

As the numbers were expected to be large, we had not originally planned to estimate uncertainty intervals. However, we opted to do so as some conditions (such as pulmonary hypertension), were found to be uncommon in the primary care data. We therefore developed the above plan to estimate the uncertainty in the

differences between primary care and trial comorbidity counts. This analysis was specified prior to examining (including examining graphically) the difference between primary care and trial comorbidity counts. The changes to this analysis plan made *after* comparing the trial and primary care data counts were in the approach used to summarise the proportion of participants with each count across trials. We had originally planned to use a simple summation weighting by the trial size or within-trial variance, but opted instead to model the uncertainty in a multinomial logit model.

However, we subsequently had difficulties fitting this model. There was a considerable amount of auto-correlation, even with thinning, and it was not clear that the model had converged even after it had been allowed to run for a very large number of iterations. The models were also slow to run and we therefore opted to use a different modelling approach.

We decided to use a Poisson model (with a log-link) because, on plotting the trial comorbidity counts, we noted that they were very closely approximated by Poisson distributions. By modelling the mean count (expectation), rather than the proportion of participants/patients with each comorbidity, we were able to greatly simplify the modelling. It meant that we could summarise the distribution of counts for each trial using a single integer (the total comorbidity count) along with an offset for the number of participants. These models appeared to converge quickly with no evidence of auto correlation.

Nonetheless, in sensitivity analyses, we compared the estimates from the Poisson model used in the main analysis to the original multinomial logistic regression model (although this showed considerable auto correlation in the MCMC samples). Both models yielded similar point estimates, and the credible intervals for the Poisson model were wider, hence we opted to use this for all subsequent analyses. We also fit fixed-effect models, assuming that all trials had the same underlying mean comorbidity count. We also fit models allowing the within-trial variation to vary between trials (but with a common prior). For these sensitivity analyses, the condition-level mean comorbidity counts for indications with more than one trial are shown in Figure S5.2). Similar results were obtained for all models.

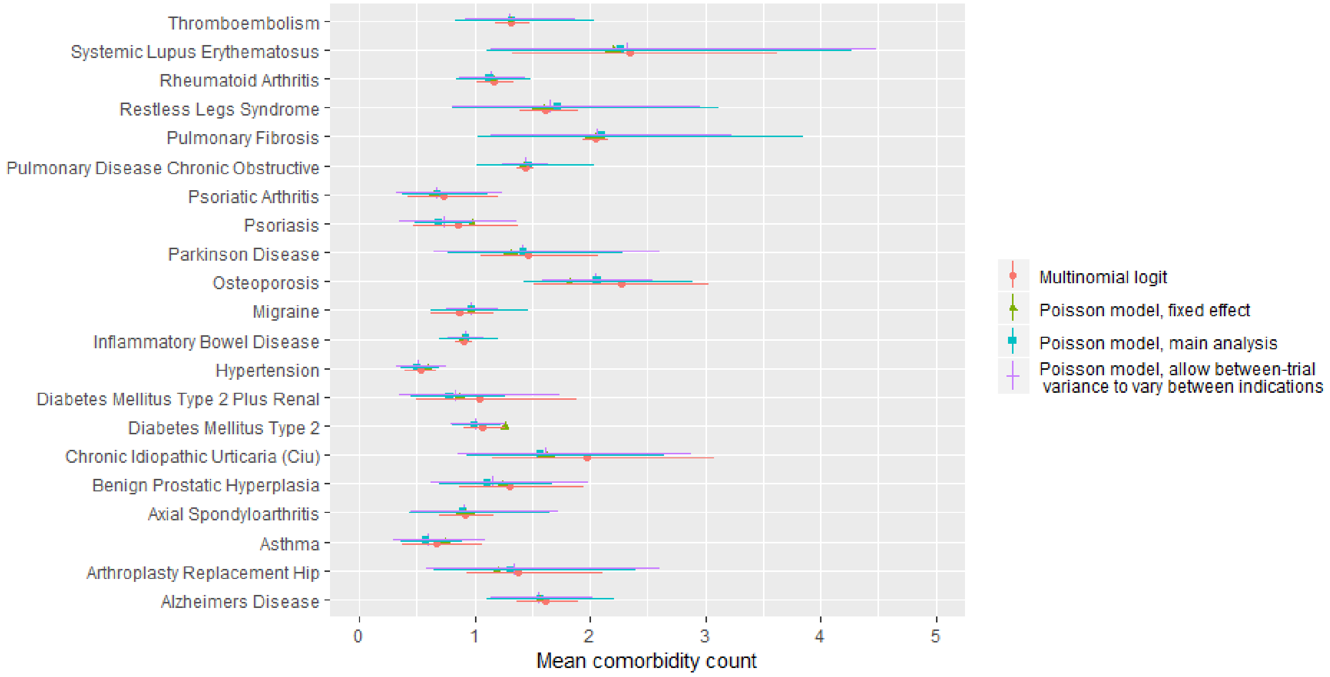

Figure S5.2: Mean comorbidity count

The code for each model shown in Figure S5.2 follows.

5.4 Multinomial logit

```
model{
for(i in 1:ns){
  r[i, 1:Categories] ~ dmulti(prob[i, 1:Categories], n[i])
  # Set phi (which is not the same as prob, for the first category, to zero)
  phi[i,1] <- 1
  prob[i,1] <- 1 / sum(phi[i, 1:Categories])
  for(c in 2:Categories){
    log(phi[i,c]) <- intercept[i, c]
    prob[i,c] <- phi[i,c] / sum(phi[i, 1:Categories])
  } # end categories

  ## Set priors and contraint at trial-level
  ## Set intercept for category one to zero
  intercept[i,1] <- 0
  ## st vague prior on other categories
  for(c in 2:Categories){
    intercept[i,c] ~ dnorm(mu[c], prec)
  }

} # end studies


  ## Global priors
    mu[1] <- 0
  for(c in 2:Categories){
    mu[c] ~ dnorm(0, 0.001)
  }
  ## common variance in odds ratio across all categories
    prec <- 1/sd^2
    sd ~ dnorm(0, 0.1)T(0,)

 ## Transform mu to estimate proportions for random effect across trials
    for(c in 1:Categories){
    log(phi_new[c]) <- mu[c]
    prob_new[c] <- phi_new[c] / sum(phi_new[1:Categories])
  } # end categories

}
"
```

5.5 Poisson model, fixed effect

```
model{
  for(i in 1:i_max){
    ## Trial level
    for(j in 1:j_max){
      ys[i,j] ~ dpois(lambda[i,j])
      log(lambda[i,j]) <- intercept[i] + log(ns[i,j])
  } # end trial
  } # end condition
    ## Prior for intercept at level of condition, note that these are independent for each co
ndition
    for (i in 1:i_max){
      intercept[i] ~ dnorm(0, 0.1)
    }
} # End of model
```

5.6 Poisson model, allow between-trial variance to vary between indications

```
model{
  for(i in 1:i_max){
    ## Trial level
    for(j in 1:j_max){
      ys[i,j] ~ dpois(lambda[i,j])
      log(lambda[i,j]) <- intercept[i,j] + log(ns[i,j])
      intercept[i,j] ~ dnorm(mu_trial[i], prec_trial[i])
  } # end trial
  } # end condition
    ## Prior for intercept at level of condition, note that these are independent for each co
ndition
    for (i in 1:i_max){
      mu_trial[i] ~ dnorm(0, 0.1)
    ## Precision in each trial
      prec_trial[i] <- 1/sd_trial[i]^2
      sd_trial[i] ~ dnorm(sd_trial_mu, sd_trial_prec)T(0,)
    }
    ## Hyperprior for between-trial precision
    sd_trial_mu ~ dnorm(0, 1)T(0,)
    sd_trial_prec <- 1/sd_trial_sd^2
    sd_trial_sd ~ dnorm(0, 1)T(0,)
} # End of model
```
