# Supplementary material for: Representation of people with comorbidity and multimorbidity in clinical trials of novel drug therapies: an individual-level participant data analysis
Source: BMC Med. 2019 Nov 12;17:201. doi: 10.1186/s12916-019-1427-1 (PMC6849229; doi:10.1186/s12916-019-1427-1)
Supplement: Supplementary file 6 — Additional file 6. Trials-characteristics.pdf: Summary of characteristics of included trials. [file 12916_2019_1427_MOESM6_ESM.pdf]

6 Trial characteristics

6.1 Indication, start-date, drug and phase

Table S6.1 shows the trial characteristics, which were obtained from the US clinical trials register, clinicaltrials.gov. The database was accessed using in a database format via the Aggregate Analysis of ClincalTrials.gov (AACT) tool from the Clinical Trials Transformation Initiative (http://www.ctti-clinicaltrials.org).

Table S6.1: Trial characteristics

nct_id	sponsor	start_date	phase	conditions	medicine
NCT00036439	Janssen	2002-02-01	Phase 3	Colitis, Ulcerative	Infliximab
NCT00046254	Novartis	2002-02-01	Phase 3	Osteoporosis	zoledronic acid
NCT00049829	Novartis	2002-01-01	Phase 3	Osteoporosis	zoledronic acid
NCT00051558	Lilly	2002-11-01	Phase 3	Osteoporosis	Teriparatide
NCT00094458	Janssen	2005-03-01	Phase 3	Crohn Disease	Infliximab
NCT00096655	Janssen	2002-05-01	Phase 3	Colitis, Ulcerative	Infliximab
NCT00100620	Novartis	2004-06-01	Phase 3	Osteoporosis	zoledronic acid
NCT00106535	Roche	2005-01-01	Phase 3	Arthritis, Rheumatoid	tocilizumab
NCT00125918	Lilly	2005-08-01	Phase 3	Hypertension, Hypertension, Pulmonary	Tadalafil
NCT00133198	Boehringer Ingelheim	2004-04-01	Phase 3	Restless Legs Syndrome	pramipexole
NCT00144508	Roche	2003-03-01	Phase 3	Arthritis, Rheumatoid	tocilizumab
NCT00153023	Boehringer Ingelheim	2003-04-01	Phase 4	Diabetic Nephropathies, Hypertension	telmisartan
NCT00153088	Boehringer Ingelheim	2003-01-01	Phase 4	Diabetic Nephropathies	telmisartan
NCT00207662	Janssen	2000-07-01	Phase 3	Crohn Disease	Infliximab
NCT00207766	Janssen	2000-06-01	Phase 3	Crohn Disease	Infliximab

NCT00210912	Janssen	2003-09-01	Phase 3	Migraine Disorders	Topiramate
NCT00212810	Janssen	2005-09-01	Phase 4	Migraine Disorders	Topiramate
NCT00216593	Janssen	2003-12-01	Phase 3	Alzheimer Disease	Galantamine
NCT00231595	Janssen	2001-03-01	Phase 3	Migraine Disorders	Topiramate
NCT00236028	Janssen		Phase 3	Arthritis, Rheumatoid	Infliximab
NCT00236431	Janssen	2001-05-01	Phase 3	Alzheimer Disease	Galantamine
NCT00236509	Janssen	2001-02-01	Phase 3	Migraine Disorders	Topiramate
NCT00236561	Janssen	2001-04-01	Phase 3	Migraine Disorders	Topiramate
NCT00236574	Janssen	2001-05-01	Phase 3	Alzheimer Disease	Galantamine
NCT00262600	Boehringer Ingelheim	2005-11-01	Phase 3	Atrial Fibrillation, Stroke	dabigatran etexilate
NCT00264537	Janssen	2005-12-01	Phase 3	Arthritis, Rheumatoid	Golimumab
NCT00264550	Janssen	2005-12-01	Phase 3	Arthritis, Rheumatoid	Golimumab
NCT00265083	Janssen	2005-12-01	Phase 3	Spondylitis, Ankylosing	Golimumab
NCT00265096	Janssen	2005-12-01	Phase 3	Arthritis, Psoriatic	Golimumab
NCT00267969	Janssen	2005-12-01	Phase 3	Psoriasis	Ustekinumab
NCT00274599	Boehringer Ingelheim	2002-10-01	Phase 4	Hypertension	telmisartan
NCT00274612	Boehringer Ingelheim	2002-10-01	Phase 4	Hypertension	telmisartan
NCT00291330	Boehringer Ingelheim	2006-02-01	Phase 3	Venous Thromboembolism	dabigatran etexilate
NCT00299546	Janssen	2006-02-01	Phase 3	Arthritis, Rheumatoid	Golimumab
NCT00307437	Janssen	2005-05-01	Phase 3	Psoriasis	Ustekinumab
NCT00329238	Boehringer Ingelheim	2006-05-01	Phase 3	Venous Thromboembolism	dabigatran etexilate
NCT00348140	GSK	2006-07-12	Phase 3	Alzheimer Disease	rosiglitazone

NCT00348309	GSK	2006-07-06	N/A	Alzheimer Disease	rosiglitazone
NCT00361335	Janssen	2006-09-01	Phase 3	Arthritis, Rheumatoid	Golimumab
NCT00384930	Lilly	2006-08-01	Phase 2/Phase 3	Prostatic Hyperplasia	Tadalafil
NCT00402233	Boehringer Ingelheim	2006-11-01	Phase 4	Parkinson Disease	pramipexole
NCT00410384	GSK	2006-12-01	Phase 3	Lupus Erythematosus, Systemic	belimumab
NCT00423085	Novartis	2007-01-01	Phase 3	Alzheimer Disease	rivastigmine
NCT00424476	GSK	2007-05-01	Phase 3	Lupus Erythematosus, Systemic	belimumab
NCT00428090	GSK	2007-02-27	Phase 3	Alzheimer Disease	rosiglitazone
NCT00439244	Novartis	2006-12-01	Phase 3	Osteoporosis	zoledronic acid
NCT00439647	Novartis	2006-12-01	Phase 3	Osteoporosis	zoledronic acid
NCT00466167	Boehringer Ingelheim	2007-04-01	Phase 3	Parkinson Disease	Pramipexol
NCT00472199	Boehringer Ingelheim	2007-05-01	Phase 4	Restless Legs Syndrome	pramipexole
NCT00479401	Boehringer Ingelheim	2007-05-01	Phase 3	Parkinson Disease	pramipexole
NCT00487539	Janssen	2007-08-01	Phase 2/Phase 3	Colitis, Ulcerative	Golimumab
NCT00488631	Janssen	2007-09-01	Phase 3	Colitis, Ulcerative	Golimumab
NCT00558259	Boehringer Ingelheim	2007-11-01	Phase 3	Venous Thromboembolism	dabigatran etexilate
NCT00670501	Lilly	1996-08-01	Phase 3	Osteoporosis	Teriparatide
NCT00680186	Boehringer Ingelheim	2008-04-01	Phase 3	Venous Thromboembolism	dabigatran etexilate
NCT00734474	Lilly	2008-08-01	Phase 2/Phase 3	Diabetes Mellitus, Type 2	Dulaglutide
NCT00783718	Takeda	2009-01-01	Phase 3	Colitis, Ulcerative	vedolizumab
NCT00827242	Lilly	2009-01-	Phase 3	Prostatic Hyperplasia	Tadalafil

		01			
NCT00848081	Lilly	2009-03-01	Phase 3	Prostatic Hyperplasia	Tadalafil
NCT00855582	Lilly	2009-03-01	Phase 3	Prostatic Hyperplasia	Tadalafil
NCT00856284	Takeda	2009-03-01	Phase 3	Diabetes Mellitus, Type 2	alogliptin
NCT00861757	Lilly	2009-03-01	Phase 3	Prostatic Hyperplasia	Tadalafil
NCT00926289	Boehringer Ingelheim	2009-06-01	Phase 4	Hypertension	telmisartan
NCT00968708	Takeda	2009-09-01	Phase 3	Acute Coronary Syndrome, Diabetes Mellitus, Type 2	alogliptin
NCT00968812	Janssen	2009-09-01	Phase 3	Diabetes Mellitus, Type 2	Canagliflozin
NCT00970632	Lilly	2009-10-01	Phase 3	Prostatic Hyperplasia	Tadalafil
NCT00973479	Janssen	2009-09-01	Phase 3	Arthritis, Rheumatoid	Golimumab
NCT01007435	Roche	2009-10-31	Phase 3	Arthritis, Rheumatoid	Tocilizumab
NCT01009086	Janssen	2009-12-01	Phase 3	Arthritis, Psoriatic	Ustekinumab
NCT01064687	Lilly	2010-02-01	Phase 3	Diabetes Mellitus, Type 2	Dulaglutide
NCT01075282	Lilly	2010-02-01	Phase 3	Diabetes Mellitus, Type 2	Dulaglutide
NCT01077362	Janssen	2010-03-01	Phase 3	Arthritis, Psoriatic	Ustekinumab
NCT01081834	Janssen	2010-03-01	Phase 3	Diabetes Mellitus, Type 2	Canagliflozin
NCT01106625	Janssen	2010-05-01	Phase 3	Diabetes Mellitus, Type 2	Canagliflozin
NCT01106651	Janssen	2010-06-01	Phase 3	Diabetes Mellitus, Type 2	Canagliflozin
NCT01106677	Janssen	2010-05-01	Phase 3	Diabetes Mellitus, Type 2	Canagliflozin
NCT01106690	Janssen	2010-06-01	Phase 3	Diabetes Mellitus, Type 2	Canagliflozin
NCT01119859	Roche	2010-05-01	Phase 4	Arthritis, Rheumatoid	Tocilizumab
NCT01126580	Lilly	2010-05-01	Phase 3	Diabetes Mellitus, Type 2	Dulaglutide

NCT01137812	Janssen	2010-07-01	Phase 3	Diabetes Mellitus, Type 2	Canagliflozin
NCT01159912	GSK	2010-06-30	Phase 3	Asthma	fluticasone furoate
NCT01164501	Boehringer Ingelheim	2010-07-01	Phase 3	Diabetes Mellitus, Type 2	empagliflozin
NCT01181895	GSK	2010-09-01	Phase 3	Asthma	vilanterol
NCT01191268	Lilly	2010-11-01	Phase 3	Diabetes Mellitus, Type 2	Dulaglutide
NCT01209702	Roche	2010-09-01	Phase 3	Spondylitis, Ankylosing	Tocilizumab
NCT01224171	Takeda	2010-11-01	Phase 3	Crohn Disease	vedolizumab
NCT01232569	Roche	2011-03-01	Phase 3	Arthritis, Rheumatoid	Tocilizumab
NCT01316900	GSK	2011-03-01	Phase 3	Pulmonary Disease, Chronic Obstructive	umeclidinium bromide
NCT01316913	GSK	2011-03-01	Phase 3	Pulmonary Disease, Chronic Obstructive	umeclidinium bromide
NCT01335464	Boehringer Ingelheim	2011-04-01	Phase 3	Idiopathic Interstitial Pneumonias	nintedanib
NCT01335477	Boehringer Ingelheim	2011-05-01	Phase 3	Idiopathic Interstitial Pneumonias	nintedanib
NCT01358578	Novartis	2011-06-01	Phase 3	Psoriasis	secukinumab
NCT01365455	Novartis	2011-06-01	Phase 3	Psoriasis	secukinumab
NCT01369329	Janssen	2011-07-01	Phase 3	Crohn Disease	Ustekinumab
NCT01369342	Janssen	2011-07-01	Phase 3	Crohn Disease	Ustekinumab
NCT01369355	Janssen	2011-09-13	Phase 3	Crohn Disease	Ustekinumab
NCT01370005	Boehringer Ingelheim	2011-06-01	Phase 3	Diabetes Mellitus, Type 2, Hypertension	empagliflozin
NCT01436110	GSK	2011-09-01	Phase 3	Asthma	fluticasone furoate
NCT01474512	Lilly	2011-11-01	Phase 3	Psoriasis	Ixekizumab
NCT01597245	Lilly	2012-05-01	Phase 3	Psoriasis	Ixekizumab
NCT01624259	Lilly	2012-06-	Phase 3	Diabetes Mellitus,	Dulaglutide

		01		Type 2	
NCT01646177	Lilly	2012-07-01	Phase 3	Psoriasis	Ixekizumab
NCT01691521	GSK	2012-10-08	Phase 3	Asthma	mepolizumab
NCT01719003	Boehringer Ingelheim	2012-10-01	Phase 3	Diabetes Mellitus, Type 2	empagliflozin
NCT01769378	Lilly	2013-01-01	Phase 3	Diabetes Mellitus, Type 2	Dulaglutide
NCT01772134	GSK	2013-01-01	Phase 3	Pulmonary Disease, Chronic Obstructive	umeclidinium bromide
NCT01957163	GSK	2013-10-01	Phase 3	Pulmonary Disease, Chronic Obstructive	umeclidinium bromide
NCT02119286	GSK	2013-10-01	Phase 3	Pulmonary Disease, Chronic Obstructive	umeclidinium bromide
NCT02172586	Boehringer Ingelheim	2000-01-01	Phase 4	Hypertension	telmisartan
NCT02175355	Boehringer Ingelheim	1999-10-01	Phase 3	Hypertension	telmisartan
NCT02177344	Boehringer Ingelheim	1998-08-01	Phase 3	Pulmonary Disease, Chronic Obstructive	ipratropium bromide
NCT02177396	Boehringer Ingelheim	1998-04-01	Phase 3	Hypertension	telmisartan
NCT02177461	Boehringer Ingelheim	2000-04-01	Phase 4	Hypertension	telmisartan
NCT02183064	Boehringer Ingelheim	1998-10-01	Phase 3	Osteoarthritis	meloxicam
NCT02236611	GSK	2014-09-26	Phase 4	Pulmonary Disease, Chronic Obstructive	umeclidinium bromide
NCT02242318	Boehringer Ingelheim	2001-09-01	Phase 4	Hypertension	telmisartan

6.2 Age and sex distribution

Table S6.2 shows the number of participants of each sex and the distribution of age by sex for each trial. The age is summarised as the mean, standard deviation, and the range for eligibility criteria. For two trials (NCT00036439 and NCT00096655) the age was redacted from the IPD, and so the mean and standard deviation were taken from the published reports, under the assumption that men and women had the same mean age. Where the maximum age was not specified in the trial, we assumed for estimating the central tendency of the truncated normal distribution that this was 100 years old.

Table S6.2: Age distribution by sex

nct_id	male	female
NCT00036439	N = 364; 41.9 (14.3); [18-100]	N = 364; 41.9 (14.3); [18-100]

NCT00046254	N = 508; 72.7 (11); [50-100]	N = 1619; 75.3 (10); [50-100]
NCT00049829		N = 7765; 73.2 (5.4); [65-89]
NCT00051558	N = 84; 56.8 (14); [21-100]	N = 345; 56.1 (13.7); [21-100]
NCT00094458	N = 262; 36 (13); [21-99]	N = 246; 37 (13); [21-99]
NCT00096655	N = 364; 40 (13.3); [18-100]	N = 364; 40 (13.3); [18-100]
NCT00100620	N = 265; 56.3 (14.4); [18-85]	N = 568; 53.6 (14.5); [18-85]
NCT00106535	N = 192; 53.7 (11.5); [18-100]	N = 957; 51.6 (12.3); [18-100]
NCT00125918	N = 88; 56.8 (15.8); [12-100]	N = 318; 52.2 (15.3); [12-100]
NCT00133198	N = 129; 50.2 (14.1); [18-80]	N = 216; 52 (12.2); [18-80]
NCT00144508	N = 59; 55.6 (10); [20-100]	N = 247; 52.2 (12.6); [20-100]
NCT00152971	N = 1099; 65.7 (9.5); [18-100]	N = 1494; 66.4 (9.5); [18-100]
NCT00153023	N = 567; 61 (9.2); [30-80]	N = 318; 61.5 (9.1); [30-80]
NCT00153088	N = 385; 61.3 (7.7); [30-74]	N = 142; 60.7 (8.2); [30-74]
NCT00168792	N = 1290; 58.9 (11.6); [18-100]	N = 386; 65.6 (11.7); [18-100]
NCT00168818	N = 1509; 61.9 (10.8); [18-100]	N = 1953; 65.5 (10.5); [18-100]
NCT00207662	N = 239; 37 (12); [18-100]	N = 334; 37 (12); [18-100]
NCT00207766	N = 144; 39 (11); [18-100]	N = 138; 39 (11); [18-100]
NCT00210912	N = 48; 40 (13); [18-100]	N = 280; 37 (12); [18-100]
NCT00212810	N = 148; 41 (11); [18-65]	N = 1066; 38 (11); [18-65]
NCT00216593	N = 81; 82 (6); [40-100]	N = 334; 83 (6); [40-100]
NCT00231595	N = 63; 35 (15); [12-65]	N = 420; 39 (13); [12-65]
NCT00236028	N = 303; 52 (12); [18-75]	N = 746; 49 (13); [18-75]
NCT00236431	N = 462; 69 (9); [50-100]	N = 533; 70 (9); [50-100]
NCT00236509	N = 55; 41 (13); [12-65]	N = 429; 40 (11); [12-65]
NCT00236561	N = 112; 40 (12); [12-65]	N = 448; 40 (12); [12-65]
NCT00236574	N = 456; 71 (8); [50-100]	N = 606; 70 (9); [50-100]
NCT00262600	N = 11479; 70.6 (8.8); [18-100]	N = 6554; 72.8 (8); [18-100]
NCT00264537	N = 109; 51 (12); [18-100]	N = 528; 49 (12); [18-100]
NCT00264550	N = 86; 54 (12); [18-100]	N = 358; 50 (11); [18-100]
NCT00265083	N = 255; 38 (12); [18-100]	N = 101; 42 (11); [18-100]
NCT00265096	N = 244; 47 (11); [18-100]	N = 160; 47 (11); [18-100]
NCT00267969	N = 531; 46 (12); [18-100]	N = 235; 44 (12); [18-100]
NCT00274599	N = 543; 52.9 (9.9); [19-100]	N = 269; 52.1 (9.7); [19-100]
NCT00274612	N = 479; 53.4 (10.3); [18-100]	N = 322; 54 (10.9); [18-100]
NCT00291330	N = 1493; 55.5 (14.4); [18-100]	N = 1068; 53.4 (17.8); [18-100]
NCT00299546	N = 94; 55 (12); [18-100]	N = 367; 54 (12); [18-100]

NCT00307437	N = 841; 46 (12); [18-100]	N = 389; 46 (13); [18-100]
NCT00329238	N = 1746; 55.5 (13.9); [18-100]	N = 1118; 53.3 (16.8); [18-100]
NCT00348140	N = 645; 72.8 (8.3); [50-90]	N = 808; 73.4 (8); [50-90]
NCT00348309	N = 578; 73.5 (7.9); [50-90]	N = 882; 74.6 (7.7); [50-90]
NCT00361335	N = 126; 53 (12); [18-100]	N = 517; 49 (12); [18-100]
NCT00384930	N = 427; 62.2 (8.3); [45-100]	
NCT00402233	N = 207; 62.9 (10.2); [31-100]	N = 104; 61 (10.2); [31-100]
NCT00410384	N = 57; 42 (12.9); [18-100]	N = 769; 40.1 (11.4); [18-100]
NCT00423085	N = 92; 74 (7.3); [50-85]	N = 196; 74.9 (7.4); [50-85]
NCT00424476	N = 44; 33.6 (9.6); [18-100]	N = 823; 35.6 (11.1); [18-100]
NCT00428090	N = 216; 72.2 (7.7); [50-90]	N = 364; 72.5 (8.6); [50-90]
NCT00439244		N = 412; 65.1 (9.1); [45-89]
NCT00439647	N = 1199; 65.8 (8.6); [50-85]	
NCT00464269	N = 198; 37.4 (12.4); [16-70]	N = 202; 37.9 (12.5); [16-70]
NCT00466167	N = 285; 61.3 (10); [32-100]	N = 233; 61.8 (9.9); [32-100]
NCT00472199	N = 133; 55.7 (12.8); [18-85]	N = 198; 57.7 (11.5); [18-85]
NCT00479401	N = 299; 61.5 (9.9); [30-100]	N = 240; 61.7 (9.5); [30-100]
NCT00487539	N = 596; 40 (11); [18-100]	N = 469; 40 (13); [18-100]
NCT00488631	N = 700; 40 (14); [18-100]	N = 528; 40 (13); [18-100]
NCT00490035	N = 228; 36.9 (12.9); [16-70]	N = 171; 36.5 (13.3); [16-70]
NCT00552058	N = 195; 36.7 (12.7); [18-75]	N = 244; 38 (12); [18-75]
NCT00558259	N = 752; 56.2 (14.2); [18-100]	N = 600; 55.4 (16.5); [18-100]
NCT00623623	N = 1509; 57.9 (11.8); [18-100]	N = 402; 65.9 (12.5); [18-100]
NCT00657150	N = 987; 60.3 (11.8); [18-100]	N = 1068; 63.8 (10.8); [18-100]
NCT00670501		N = 1732; 68.9 (7); [30-85]
NCT00680186	N = 1568; 54.3 (15.4); [18-100]	N = 1017; 55.7 (17.3); [18-100]
NCT00694382	N = 1930; 60.3 (10.4); [18-100]	N = 1282; 58.5 (10.8); [18-100]
NCT00734474	N = 559; 53.5 (9.8); [18-75]	N = 643; 53.7 (9.9); [18-75]
NCT00783718	N = 525; 39.8 (13.1); [18-80]	N = 370; 39.8 (13.1); [18-80]
NCT00827242	N = 325; 64.1 (9.2); [45-100]	
NCT00848081	N = 318; 66.6 (9.2); [45-100]	
NCT00855582	N = 606; 62.1 (8.1); [45-100]	
NCT00856284	N = 1312; 55.5 (9.8); [18-80]	N = 1327; 55.3 (9.6); [18-80]
NCT00861757	N = 629; 62.5 (7.8); [45-100]	
NCT00926289	N = 479; 55.1 (11.5); [18-100]	N = 413; 59.2 (11.1); [18-100]
NCT00968708	N = 3674; 59.8 (9.7); [18-100]	N = 1736; 63.3 (9.9); [18-100]

NCT00968812	N = 757; 56 (9); [18-80]	N = 695; 56 (9); [18-80]
NCT00970632	N = 511; 63 (8.2); [45-100]	
NCT00973479	N = 109; 53 (12); [18-100]	N = 483; 52 (12); [18-100]
NCT01007435	N = 294; 53 (12.8); [18-100]	N = 1105; 49.3 (13.4); [18-100]
NCT01009086	N = 330; 46 (11); [18-100]	N = 285; 49 (13); [18-100]
NCT01064687	N = 571; 55.5 (9.8); [18-100]	N = 407; 54.6 (9.8); [18-100]
NCT01075282	N = 421; 56.7 (9.2); [18-100]	N = 408; 55.7 (9.7); [18-100]
NCT01077362	N = 148; 47 (11); [18-100]	N = 164; 49 (12); [18-100]
NCT01081834	N = 302; 54 (11); [18-80]	N = 376; 54 (11); [18-80]
NCT01087762	N = 200; 40.1 (11.6); [18-100]	N = 125; 38.9 (11.7); [18-100]
NCT01087788	N = 183; 45.9 (10.2); [18-100]	N = 226; 48.3 (11.3); [18-100]
NCT01106625	N = 239; 57 (9); [18-80]	N = 230; 56 (9); [18-80]
NCT01106651	N = 396; 64 (6); [55-80]	N = 320; 63 (6); [55-80]
NCT01106677	N = 605; 55 (10); [18-80]	N = 679; 55 (9); [18-80]
NCT01106690	N = 217; 58 (10); [18-80]	N = 127; 55 (10); [18-80]
NCT01119859	N = 63; 55.6 (12.1); [18-100]	N = 263; 53.4 (12.8); [18-100]
NCT01126580	N = 353; 55.7 (9.9); [18-100]	N = 454; 54.6 (10.7); [18-100]
NCT01137812	N = 422; 56 (9); [18-100]	N = 333; 57 (10); [18-100]
NCT01159912	N = 144; 37.5 (16.9); [12-100]	N = 205; 42.6 (15.9); [12-100]
NCT01164501	N = 432; 64.1 (8.8); [18-100]	N = 309; 63.6 (8.9); [18-100]
NCT01181895	N = 143; 39 (17.1); [12-100]	N = 204; 42.9 (16.9); [12-100]
NCT01191268	N = 475; 59.1 (9.6); [18-100]	N = 417; 58.1 (8.7); [18-100]
NCT01209702	N = 76; 41.6 (12); [18-100]	N = 26; 43.8 (11.8); [18-100]
NCT01224171	N = 180; 36.8 (12.7); [18-80]	N = 236; 37.9 (12.7); [18-80]
NCT01232569	N = 100; 54.7 (11.2); [18-100]	N = 556; 51.6 (11.5); [18-100]
NCT01264939	N = 94; 44.6 (14.3); [12-75]	N = 242; 42.6 (14.1); [12-75]
NCT01287117	N = 87; 39.4 (14.9); [12-75]	N = 232; 42 (14.4); [12-75]
NCT01292473	N = 78; 45.1 (13.4); [12-75]	N = 245; 41.7 (13.7); [12-75]
NCT01316900	N = 585; 63.5 (8.7); [40-100]	N = 261; 61.6 (9.6); [40-100]
NCT01316913	N = 592; 65.6 (8.2); [40-100]	N = 280; 62.7 (8.6); [40-100]
NCT01335464	N = 415; 66.7 (8.2); [40-100]	N = 99; 67.7 (8.9); [40-100]
NCT01335477	N = 428; 66.8 (7.8); [40-100]	N = 121; 66.2 (7.6); [40-100]
NCT01358578	N = 722; 44.3 (12.6); [18-100]	N = 298; 43.6 (14.1); [18-100]
NCT01365455	N = 474; 44.7 (13); [18-100]	N = 220; 45.5 (13.7); [18-100]
NCT01369329	N = 329; 38 (13); [18-100]	N = 440; 37 (12); [18-100]
NCT01369342	N = 298; 38 (13); [18-99]	N = 342; 41 (14); [18-99]

NCT01369355	N = 627; 38 (13); [18-99]	N = 782; 39 (13); [18-99]
NCT01370005	N = 496; 60.3 (8.6); [18-100]	N = 329; 60.1 (9.6); [18-100]
NCT01436110	N = 118; 32 (17.2); [12-100]	N = 233; 39 (15.9); [12-100]
NCT01474512	N = 883; 45.7 (12.7); [18-100]	N = 413; 45.7 (13.4); [18-100]
NCT01597245	N = 819; 45.1 (12.8); [18-100]	N = 402; 44.8 (13.6); [18-100]
NCT01624259	N = 287; 56.3 (9.2); [18-100]	N = 312; 56.2 (10); [18-100]
NCT01646177	N = 914; 45.6 (12.9); [18-100]	N = 427; 46 (13.4); [18-100]
NCT01691521	N = 247; 50.2 (14.1); [12-100]	N = 329; 50.1 (14.5); [12-100]
NCT01719003	N = 811; 53 (10.7); [18-100]	N = 607; 52 (11.2); [18-100]
NCT01769378	N = 133; 56.8 (9.8); [18-100]	N = 167; 57.7 (9.6); [18-100]
NCT01772134	N = 409; 64.3 (8.4); [40-100]	N = 208; 61 (8); [40-100]
NCT01957163	N = 407; 64.6 (8.4); [40-100]	N = 212; 64 (7.6); [40-100]
NCT02119286	N = 391; 63.7 (8.3); [40-100]	N = 229; 61.6 (8); [40-100]
NCT02172586	N = 256; 51.4 (10); [18-100]	N = 106; 54.7 (9); [18-100]
NCT02175355	N = 449; 62 (11.2); [35-84]	N = 590; 63.8 (10.6); [35-84]
NCT02177344	N = 370; 66.1 (8.6); [40-100]	N = 275; 65.1 (8.4); [40-100]
NCT02177396	N = 290; 53.8 (10.1); [18-100]	N = 136; 52.4 (9.5); [18-100]
NCT02177461	N = 181; 70.9 (5.6); [65-100]	N = 192; 70.5 (4.7); [65-100]
NCT02181985	N = 4667; 59 (11.8); [18-100]	N = 1424; 66.1 (11.7); [18-100]
NCT02181998	N = 1258; 60.1 (12.1); [18-100]	N = 372; 68.9 (12.1); [18-100]
NCT02183064	N = 432; 63.5 (11.8); [18-100]	N = 888; 63.9 (11.5); [18-100]
NCT02236611	N = 707; 63.8 (8.1); [40-100]	N = 330; 64.7 (8.7); [40-100]
NCT02242318	N = 272; 53.4 (9.6); [18-100]	N = 174; 53.4 (10.6); [18-100]

6.3 Eligibility criteria

Trial eligibility criteria can be obtained from clinicaltrials.gov. As exemplars, criteria for the first 3 trials is shown below (Table S6.3).

Table S6.3: Eligibility criteria

nct_id **criteria**

NCT02242318Inclusion Criteria:

```
      1. Mild-to-moderate hypertension defined as a mean seated diastolic blood pressur
e of â‰¥

         95 mmHg and â‰¤ 109 mmHg, measured by manual cuff sphygmomanometer, at Visit 2


      2. 24-hour mean DBP of â‰¥ 85 mmHg at Visit 3 as measured by ABPM
```

```
      3. Age 18 years or older


      4. Ability to stop any current antihypertensive therapy without risk to the patie
nt

         (investigator's discretion)


      5. Patient's written informed consent in accordance with Good Clinical Practice (
GCP) and

         local legislation


    Exclusion Criteria:


      1. Pre-menopausal women (last menstruation â‰¤ 1 year prior to start of run-in pe
riod) who


           1. are not surgically sterile,


           2. are nursing,


           3. are of child-bearing potential and are NOT practising acceptable methods
of birth

              control, or do NOT plan to continue practising an acceptable method throu
ghout

              the study. Acceptable methods of birth control include oral, implantable
or

              injectable contraceptives and Intra Uterine Devices (IUD)


      2. Known or suspected secondary hypertension


      3. Mean sitting SBP â‰¥180 mmHg or mean sitting DBP â‰¥110 mmHg during any visit
of the

         placebo run-in period


      4. Hepatic and/or renal dysfunction as defined by the following laboratory parame
ters:


           1. Serum Glutamate-Pyruvate-Transaminase (Alanine Aminotransferase) (SGPT (A
LT)) or

              Serum Glutamate-Oxaloacetate-Transaminase (Aspartate Aminotransferase) (S
GOT

              (AST)) > than 2 times the upper limit of normal range,


           2. Serum creatinine > 2.3 mg/dL (or > 203 Î¼mol/l)
```

5. Bilateral renal artery stenosis, renal artery stenosis in a solitary kidney, patients postrenal transplant or with only one kidney
6. Clinically relevant sodium depletion, hypokalaemia or hyperkalaemia
7. Uncorrected volume depletion
8. Primary aldosteronism
9. Hereditary fructose intolerance
10. Biliary obstructive disorders
11. Patients who have previously experienced symptoms characteristic of angioedema during treatment with ACE inhibitors or angiotensin II receptor antagonists
12. History of drug or alcohol dependency within six months prior to start of run-in period
13. Concomitant administration of any medications known to affect blood pressure, except medication allowed by the protocol
14. Any investigational therapy within one month of signing the informed consent form
15. Congestive heart failure (New York Heart Association (NYHA) functional class Congestive Heart Failure (CHF III-IV))
16. Unstable angina within the past three months prior to start of run-in period
17. Stroke within the past six months prior to start of run-in period
18. Myocardial infarction or cardiac surgery within the past three months prior to start of run-in period

```
     19. Percutaneous Transluminal Coronary Angioplasty (PTCA) within the past three mo
nths

         prior to start of run-in period


     20. Sustained ventricular tachycardia, atrial fibrillation, atrial flutter or othe
r

         clinically relevant cardiac arrhythmias as determined by the investigator


     21. Hypertrophic obstructive cardiomyopathy, aortic stenosis, hemodynamically rele
vant

         stenosis of the aortic or mitral valve


     22. Patients with insulin-dependent diabetes mellitus whose diabetes has not been
stable

         and controlled for at least the past three months as defined by an HbA1C â‰¥ 1
0%


     23. Night shift workers who routinely sleep during the daytime and whose work hour
s

         include midnight to 4:00 Ante Meridiem (AM)


     24. Known hypersensitivity to any component of the formulations


     25. Any clinical condition which, in the opinion of the investigator would not all
ow safe

         completion of the protocol and safe administration of trial medication


     26. Inability to comply with the protocol
```

NCT02236611 Inclusion Criteria:

```
       -  Type of subject: outpatient


       -  Informed Consent: a signed and dated written informed consent prior to study

          participation


       -  Age: subjects 40 years of age or older at Visit 1.


       -  Gender: male and female subjects are eligible to participate in the study. A f
emale is

          eligible to enter and participate in the study if she is of: Non-child bearing

          potential i.e., physiologically incapable of becoming pregnant, including any
female
```

```
        who is post-menopausal or surgically sterile. Surgically sterile females are d
efined
        as those with a documented hysterectomy and/or bilateral oophorectomy or tubal
        ligation. Post-menopausal females are defined as being amenorrhoeic for greate
r than 1
        year with an appropriate clinical profile, eg, age appropriate, > 45 years, in
the
        absence of hormone replacement therapy OR child bearing potential, has a negat
ive
        pregnancy test at screening, and agrees to one of the acceptable contraceptive
methods
        used consistently and correctly i.e., in accordance with the approved product
label
        and the instructions of the physician for the duration of the study - screenin
g to
        follow-up contact.

      - Diagnosis: an established clinical history of COPD in accordance with the defi
nition
        by the American Thoracic Society/European Respiratory Society (ERS)

      - Smoking history: current or former cigarette smokers with a history of cigaret
te
        smoking of >= 10 pack-years [number of pack years = (number of cigarettes per
day /
        20) x number of years smoked (eg. 20 cigarettes per day for 10 years, or 10 ci
garettes
        per day for 20 years both equal 10 pack-years)]. Former smokers are defined as
those
        who have stopped smoking for at least 6 months prior to Visit 1. Pipe and/or c
igar use
        cannot be used to calculate pack-year history

      - Severity of Disease: A pre and post-albuterol/salbutamol forced expiratory vol
ume in
        one second/ forced vital capacity (FEV1/FVC ratio of <0.70 and a
        post-albuterol/salbutamol FEV1 of >=30% and =<70% of predicted normal values a
t Visit
        1. Predicted values will be based upon the ERS Global Lung Function Initiative

      - Dyspnea: A score of >=2 on the modified medical research council dyspnea scale
(mMRC)
        at Visit 1

    Exclusion Criteria:
```

- Pregnancy: women who are pregnant or lactating or are planning on becoming pregnant during the study.

- Asthma: a current diagnosis of asthma.

- Other respiratory disorders: known alpha-1 antitrypsin deficiency, active lung infections (such as tuberculosis), and lung cancer are absolute exclusionary conditions. A subject who, in the opinion of the investigator, has any other significant respiratory conditions in addition to COPD should be excluded. Examples may include clinically significant bronchiectasis, pulmonary hypertension, sarcoidosis, or interstitial lung disease.

- Other diseases/abnormalities: any subject who is considered unlikely to survive the duration of the study period or has any rapidly progressing disease or immediate life-threatening illness (e.g. cancer). In addition, any subject who has any condition (e.g. neurological condition) that is likely to affect respiratory function should not be included in the study.

- Severe hepatic impairment: patients with severe hepatic impairment (Child-Pugh class C) should be excluded unless, in the opinion of the investigator, the benefit is likely to outweigh the risk.

- Severe renal impairment: patients with severe renal impairment (e.g., end-stage renal disease requiring dialysis) should be excluded, unless in the opinion of the investigator, the benefit is likely to outweigh the risk.

- Unstable or life threatening cardiac disease: long-acting muscarinic antagonists (LAMA) should be used with caution in subjects with severe cardiovascular disease. In the opinion of the investigator, use should only be considered if the benefit is likely to outweigh the risk in conditions such as: Myocardial infarction or unstable angina in the last 6 months, Unstable or life threatening cardiac arrhythmia requiring

intervention in the last 3 months, New York Heart Association (NYHA) Class IV heart failure

- Contraindications: Any history of allergy or hypersensitivity to any anticholinergic/muscarinic receptor antagonist, sympathomimetic, lactose/milk protein or magnesium stearate.

- Antimuscarinic effects: Subjects with medical conditions such as narrow-angle glaucoma, urinary retention, prostatic hypertrophy, or bladder neck obstruction should only be included if, in the opinion of the study physician, the benefit outweighs the risk.

- Hospitalization: hospitalization for COPD or pneumonia within 12 weeks prior to Visit 1.

- Lung resection: lung volume reduction surgery within the 12 months prior to Visit 1.

- 12-Lead electrocardiogram (ECG): Investigators will be provided with ECG reviews conducted by a centralized independent cardiologist to assist in evaluation of subject eligibility. The Investigator will determine the clinical significance of each abnormal ECG finding in relation to the subject's medical history and exclude subjects who would be at undue risk by participating in the trial. Subjects with the following abnormalities are excluded from participation in the study: Atrial fibrillation with rapid ventricular rate >120 beats per minute; sustained or nonsustained ventricular tachycardia; second degree heart block Mobitz type II or third degree heart block (unless pacemaker or defibrillator had been inserted)

- Medication prior to spirometry: unable to withhold albuterol/salbutamol for the 4 hour period required prior to spirometry testing at each study visit.

- Medications prior to screening: use of the following medications according to the following defined time intervals prior to Visit 1: depot corticosteroids 12 weeks, systemic, oral or parenteral corticosteroids 6 weeks, antibiotics (for lower respiratory tract infection) 6 weeks, inhaled long acting beta2 agonists/ inhaled corticosteroid (LABA/ICS) combination products if LABA/ICS therapy is discontinued completely 30 days; LABA/ICS combination products only If discontinuing ICS/ LABA therapy and switching to ICS monotherapy 48 hours for the salmeterol or formoterol component 14 days for the vilanterol component (note: the dose of ICS must be a dose of fluticasone propionate (FP) or equivalent but not to exceed 1000 mcg/day), use of ICS at a dose >1000 microgram (mcg)/day of FP or equivalent 30 days (note: use of ICS is permitted provided the dose does not exceed 1000 mcg of FP or equivalent; ICS use not to be initiated or discontinued within 30 days prior to Visit 1, except for subjects on LABA/ICS therapy who may discontinue the ICS/LABA product as indicated in the table above and switch to ICS monotherapy); initiation or discontinuation of ICS use 30 days (note: use of ICS is permitted provided the dose does not exceed 1000 mcg of FP or equivalent; ICS use not to be initiated or discontinued within 30 days prior to Visit 1, except for subjects on LABA/ICS therapy who may discontinue the ICS/LABA product as indicated in the table above and switch to ICS monotherapy); phosphodiesterase 4 (PDE4) Inhibitor (roflumilast) 14 days; LABA: salmeterol and formoterol 48 hours; olodaterol, indacaterol, and vilanterol 14 days; LAMA: tiotropium, aclidinium, glycopyrronium, umeclidinium 7 days; LAMA/LABA combination products if LAMA/LABA therapy is discontinued completely then apply whichever mono component has the longest washout; theophyllines 48 hours; Oral beta2-agonists: long-acting 48 hours, short-acting 12 hours; inhaled short acting beta2-agonists 4 hours (note: use of study provided albuterol/salbutamol is permitted during the study, except in the 4-hour period prior to spirometry testing); inhaled short-acting

```
        anticholinergics 4 hours; inhaled short-acting anticholinergic/short-acting
        beta2-agonist combination products 4 hours; any other investigational medicati
on 30
        days or within 5 drug half-lives (whichever is longer).

      - Oxygen: use of long-term oxygen therapy (LTOT) described as oxygen therapy pre
scribed
        for greater than 12 hours a day. As-needed oxygen use (i.e. =<12 hours per day
) is not
        exclusionary.

      - Nebulized therapy: regular use (prescribed for use every day, not for as-neede
d use)
        of short-acting bronchodilators (e.g. albuterol/salbutamol) via nebulized ther
apy.

      - Pulmonary rehabilitation program: participation in the acute phase of a pulmon
ary
        rehabilitation program within 4 weeks prior to Visit 1. Subjects who are in th
e
        maintenance phase of a pulmonary rehabilitation program are not excluded.

      - Drug or alcohol abuse: A known or suspected history of alcohol or drug abuse w
ithin 2
        years prior to Visit 1.

      - Affiliation with investigator site: is an investigator, sub-investigator, stud
y
        coordinator, employee of a participating investigator or study site, or immedi
ate
        family member of the aforementioned that is involved in this study.

      - Inability to read: in the opinion of the investigator, any subject who is unab
le to
        read and/or would not be able to complete a questionnaire
```

NCT02183064 Inclusion Criteria:

```
      - Males and females over the age of 18

      - The patient, if female and of reproductive potential (i.e. neither surgically
        sterilized nor post-menopausal), must be practicing adequate contraception (e.
g.
        intrauterine device, contraceptive pills, Depo-Provera, or implant or double b
arrier
```

```
         device) for at least three months prior to and for the duration of their trial
         participation and must have a negative pregnancy test at screening. Abstinence
is not
         considered to be an acceptable method of contraception. (It should be noted th
at
         NSAIDs might interfere with the effectiveness of intrauterine devices)

      -  The patient must have a documented diagnosis of at least one of the following:

           -  Osteoarthritis of the hip

           -  Osteoarthritis of the knee

           -  Osteoarthritis of the hand or

           -  Osteoarthritis of the spine - Patients must have radiographic confirmatio
n of the
              diagnosis

      -  The patient is willing to change or requires a change in current prescription
NSAID
         therapy or requires initiation of prescription NSAID therapy for treatment of
OA of
         the hip, knee, hand or spine

      -  The patient intends to remain a member of their present Managed Care Organizat
ion
         (MCO) for the duration of the trial

      -  The patient is willing to comply with instructions and to provide written info
rmed
         consent

    Exclusion Criteria:

      -  The patient has a known or suspected hypersensitivity to the trial drugs or th
eir
         excipients, analgesics, antipyretics or NSAIDS (prescription or over-the-count
er)

      -  The patient has received an investigational drug or used an investigational de
vice
         within 30 days prior to entering the trial
```

- In the opinion of the investigator the patient has any disease or condition that may result in altered absorption, excess accumulation or impaired metabolism or excretion of the trial medication
- The patient has a history of recurrent peptic ulcer or history (within the past 6 months) of gastrointestinal perforation, peptic ulceration documented by endoscopy or radiography, symptomatic hiatal hernia requiring daily treatment or any history of a gastrointestinal tract hemorrhage, except simple hemorrhoidal bleeding
- The patient is currently on coumadin or might be placed on coumadin during the course of the clinical trial
- Patients with dementia, i.e. incapable of following directions or complying with the study protocol
- Patients with co-existing rheumatological disorders including rheumatoid arthritis
- The patient has previously participated in this trial
- Patients with coexisting fibromyalgia or ankylosing spondylitis
- Patient is pregnant or lactating
- Patient has severe hepatic failure
- Patient has non-dialysed renal failure
- Patient has history of GI bleed within the past 6 months
- Patient has history of cerebrovascular bleeding or other bleeding disorders
- Patient is receiving concomitant lithium, heparin or ticlopidine therapy

```
      -  Patient has a history of leukopenia (White Blood Cell count < 3500/mmÂ³)


      -  The patient has a history of platelet count below the lower limit of normal or
has a
         documented abnormal prothrombin time (PT) or partial thromboplastin time (PTT)
```
