# Supplementary material for: Representation of people with comorbidity and multimorbidity in clinical trials of novel drug therapies: an individual-level participant data analysis
Source: BMC Med. 2019 Nov 12;17:201. doi: 10.1186/s12916-019-1427-1 (PMC6849229; doi:10.1186/s12916-019-1427-1)
Supplement: Supplementary file 7 — Additional file 7. Characteristics-of-primary-care-populations-with-each-of-the-trial-indications.pdf: Summary statistics of community sample for each index condition. [file 12916_2019_1427_MOESM7_ESM.pdf]

7 Characteristics of primary care populations with each of the trial indications

Mean age (and standard deviation) of primary care patients identified as having each of the trial indications is shown in Table S7.1.

Table S7.1: Primary Care mean age and standard deviation

index_condition	n	mean	sd
Alzheimer's Disease	5144	81.6	8.5
Asthma	191160	45.6	22.9
Atrial Fibrillation	43330	74.7	11.9
Axial Spondyloarthritis	1982	52.4	15.3
Benign Prostatic Hyperplasia	19906	72.0	10.0
Dementia (any)	13871	82.1	9.0
Diabetes Mellitus, Type 2	82473	65.3	13.0
Epilepsy	29554	45.8	21.0
Erectile dysfunction	65736	60.0	13.1
Hip arthroplasty	3084	68.2	11.0
Hypertension	310691	67.0	12.9
Hypertension, Pulmonary	759	60.5	27.0
Inflammatory bowel disease	12514	52.3	17.8
Knee arthroplasty	3433	68.7	9.3
Migraine	19562	43.5	15.6
Myocardial infarction	3510	70.7	14.1
Osteoarthritis	124521	67.6	12.7
Osteoporosis	38212	72.8	12.2
Parkinson's disease (all)	4998	74.9	10.4
Parkinson's disease (excluding secondary)	4727	75.0	10.2
Psoriasis	52810	49.1	19.0
Psoriatic arthropathy	3523	54.1	14.0
Pulmonary Disease, Chronic Obstructive	57378	69.1	11.6
Pulmonary fibrosis	1465	73.3	10.9
Restless legs syndrome	11480	63.7	15.5
Rheumatoid arthritis	13809	62.2	15.5
Systemic Lupus Erythematosus	1033	52.8	15.5
Thromboembolism	9162	66.1	15.7

Previous Next
