# Supplementary material for: Representation of people with comorbidity and multimorbidity in clinical trials of novel drug therapies: an individual-level participant data analysis
Source: BMC Med. 2019 Nov 12;17:201. doi: 10.1186/s12916-019-1427-1 (PMC6849229; doi:10.1186/s12916-019-1427-1)
Supplement: Supplementary file 8 — Additional file 8. Summary-Statistics-Comparing-Ipd-Trials-To-Wider-Body-Of-Trials-From-Clinicaltrials.Gov.Pdf: Comparison of included trials with registered trials on clinicaltrials.gov for which individual participant data were not available. [file 12916_2019_1427_MOESM8_ESM.pdf]

8 Summary statistics comparing IPD trials to wider body of trials from clincialtrials.gov

All of the following data were obtained from clincialtrials.gov, either directly or having been processed as described in the Specific interventions section.

8.1 Indications

The indications for the IPD trials were broadly similar to those where we do not have IPD. However, inflammatory bowel disease and arthritis were more frequent indications in the IPD trials Table S8.1.

Table S8.1: Number (%) of trials with selected indications for IPD and non-IPD trials

Level	Non-IPD	IPD
Number of trials	2126	114
Arrhythmias, Cardiac (C14.280.067)	38 (1.8%)	1 (0.9%)
Arthritis (C05.550.114)	205 (9.6%)	17 (14.9%)
Atherosclerosis (C14.907.137.126.307)	13 (0.6%)	0
Brain Ischemia (C14.907.253.092)	9 (0.4%)	0
Death, Sudden, Cardiac (C14.280.383.220)	1 (0%)	0
Dementia (C10.228.140.380)	30 (1.4%)	7 (6.1%)
Diabetes Mellitus (C18.452.394.750)	38 (1.8%)	0
Diabetes Mellitus, Type 1 (C18.452.394.750.124)	37 (1.7%)	0
Diabetes Mellitus, Type 2 (C18.452.394.750.149)	422 (19.8%)	17 (14.9%)
Diabetic Nephropathies (C19.246.099.875)	3 (0.1%)	1 (0.9%)
Embolism and Thrombosis (C14.907.355)	66 (3.1%)	4 (3.5%)
Enuresis (C12.777.934.284)	8 (0.4%)	0
Epilepsy (C10.228.140.490)	40 (1.9%)	0
Esophageal Diseases (C06.405.117)	40 (1.9%)	0
Gastritis (C06.405.205.697)	1 (0%)	0
Heart Failure (C14.280.434)	46 (2.2%)	0
Hyperlipidemias (C18.452.584.500.500)	75 (3.5%)	0
Hypertension (C14.907.489)	216 (10.2%)	9 (7.9%)
Hypertension, Pulmonary (C08.381.423)	9 (0.4%)	1 (0.9%)
Idiopathic Interstitial Pneumonias (C08.381.483.487)	6 (0.3%)	2 (1.8%)
Inflammatory Bowel Diseases (C06.405.469.432)	22 (1%)	11 (9.6%)

Level	Non-IPD	IPD
Intermittent Claudication (C14.907.137.126.669)	3 (0.1%)	0
Lung Diseases, Obstructive (C08.381.495)	323 (15.2%)	11 (9.6%)
Lupus Erythematosus, Systemic (C20.111.590)	10 (0.5%)	2 (1.8%)
Migraine Disorders (C10.228.140.546.399.750)	18 (0.8%)	5 (4.4%)
Multiple Sclerosis (C10.314.350.500)	8 (0.4%)	0
Myocardial Ischemia (C14.280.647)	2 (0.1%)	0
Myocardial Ischemia (C14.280.647Y)	60 (2.8%)	0
Myocardial Ischemia, acute (C14.280.647X)	91 (4.3%)	1 (0.9%)
Myocardial Ischemia, chronic (C14.280.647Y)	10 (0.5%)	0
Ocular Hypertension (C11.525)	10 (0.5%)	0
Osteoporosis (C05.116.198.579)	37 (1.7%)	7 (6.1%)
Parapsoriasis (C17.800.859.575)	1 (0%)	0
Parkinson Disease (C10.574.812)	35 (1.6%)	3 (2.6%)
Peripheral Vascular Diseases (C14.907.617)	5 (0.2%)	0
Prostatic Hyperplasia (C12.294.565.500)	26 (1.2%)	6 (5.3%)
Psoriasis (C17.800.859.675)	41 (1.9%)	7 (6.1%)
Raynaud Disease (C14.907.617.812)	1 (0%)	0
Restless Legs Syndrome (C10.803)	15 (0.7%)	2 (1.8%)
Rhinitis (C08.460.799)	41 (1.9%)	0
Stroke (C14.907.253.855)	28 (1.3%)	0
Urinary Bladder, Overactive (C23.888.942.343.780)	33 (1.6%)	0
Urticaria (C17.800.862.945)	3 (0.1%)	0

8.2 Drug classes

There was a consistent pattern for drug classes, with immunosuppressant drugs being over-represented (Table S8.2).

Table S8.2: Number (%) of trials including drug classes for IPD and non-IPD trials

Level	Non-IPD	IPD
Number of trials	2198	117
A02 DRUGS FOR ACID RELATED DISORDERS except A02BB	49 (2.2%)	0
A10A INSULINS AND ANALOGUES	159 (7.2%)	0

Level	Non-IPD	IPD
A10B BLOOD GLUCOSE LOWERING DRUGS, EXCL. INSULINS	344 (15.7%)	22 (18.8%)
B01 ANTITHROMBOTIC AGENTS	198 (9%)	5 (4.3%)
C01 CARDIAC THERAPY DRUGS	59 (2.7%)	0
C02 ANTIHYPERTENSIVES	11 (0.5%)	1 (0.9%)
C03 DIURETICS	34 (1.5%)	2 (1.7%)
C04 PERIPHERAL VASODILATORS	4 (0.2%)	0
C05 VASOPROTECTIVES	23 (1%)	0
C07 beta-Adrenergic Blocking Agents	28 (1.3%)	1 (0.9%)
C08 CALCIUM CHANNEL BLOCKERS	19 (0.9%)	0
C09 AGENTS ACTING ON THE RENIN-ANGIOTENSIN SYSTEM	171 (7.8%)	6 (5.1%)
C10 LIPID MODIFYING AGENTS	164 (7.5%)	0
G04BD Drugs for urinary frequency and incontinence	42 (1.9%)	0
G04BE Drugs used in erectile dysfunction	11 (0.5%)	7 (6%)
G04C DRUGS USED IN BENIGN PROSTATIC HYPERTROPHY	20 (0.9%)	0
H05 CALCIUM HOMEOSTASIS	3 (0.1%)	3 (2.6%)
L01 ANTINEOPLASTIC AGENTS	44 (2%)	2 (1.7%)
L04 IMMUNOSUPPRESSANTS	196 (8.9%)	37 (31.6%)
M01A ANTIINFLAMMATORY AND ANTIRHEUMATIC PRODUCTS, NON-STEROIDS	68 (3.1%)	1 (0.9%)
M04 ANTIGOUT PREPARATIONS	14 (0.6%)	0
M05 DRUGS FOR TREATMENT OF BONE DISEASES	25 (1.1%)	4 (3.4%)
N03 ANTIEPILEPTICS	53 (2.4%)	4 (3.4%)
N04 ANTI-PARKINSON DRUGS	41 (1.9%)	5 (4.3%)
N06D ANTI-DEMENTIA DRUGS	22 (1%)	4 (3.4%)
R03DX Other systemic drugs for obstructive airway diseases in ATC	78 (3.5%)	1 (0.9%)
R06 ANTIHISTAMINES FOR SYSTEMIC USE	5 (0.2%)	0
SELECTED DRUGS FOR OBSTRUCTIVE AIRWAY DISEASES bronchodilator	94 (4.3%)	4 (3.4%)
SELECTED DRUGS FOR OBSTRUCTIVE AIRWAY DISEASES glucocorticosteroid	219 (10%)	8 (6.8%)

8.3 Phase and number of arms

Trials with and without IPD were broadly similar as regards phase and number of arms, although Phase 4 trials were under-represented (Table 8.3).

Table S8.3: Number (%) of trials according to phase and number of arms for IPD and non-IPD trials

Measure	Level	Non-IPD	IPD
Number of Arms	Number of trials	2058	114
Number of Arms	1	9 (0.4%)	1 (0.9%)
Number of Arms	2	1334 (64.8%)	45 (39.5%)
Number of Arms	3	419 (20.4%)	42 (36.8%)
Number of Arms	4 or above	296 (14.4%)	26 (22.8%)
Phase	Number of trials	2058	114
Phase	Phase 2/Phase 3	38 (1.8%)	3 (2.6%)
Phase	Phase 3	1589 (77.2%)	99 (86.8%)
Phase	Phase 4	431 (20.9%)	12 (10.5%)

8.4 Trial enrolment

Figure S8.1 shows the relationship between the number of enrolled participants and whether or not we had access to IPD. The IPD trials covered a similar range, but both smaller and, especially larger trials were under-represented, consistent with the observation that Phase 4 trials were less common among the IPD trials.

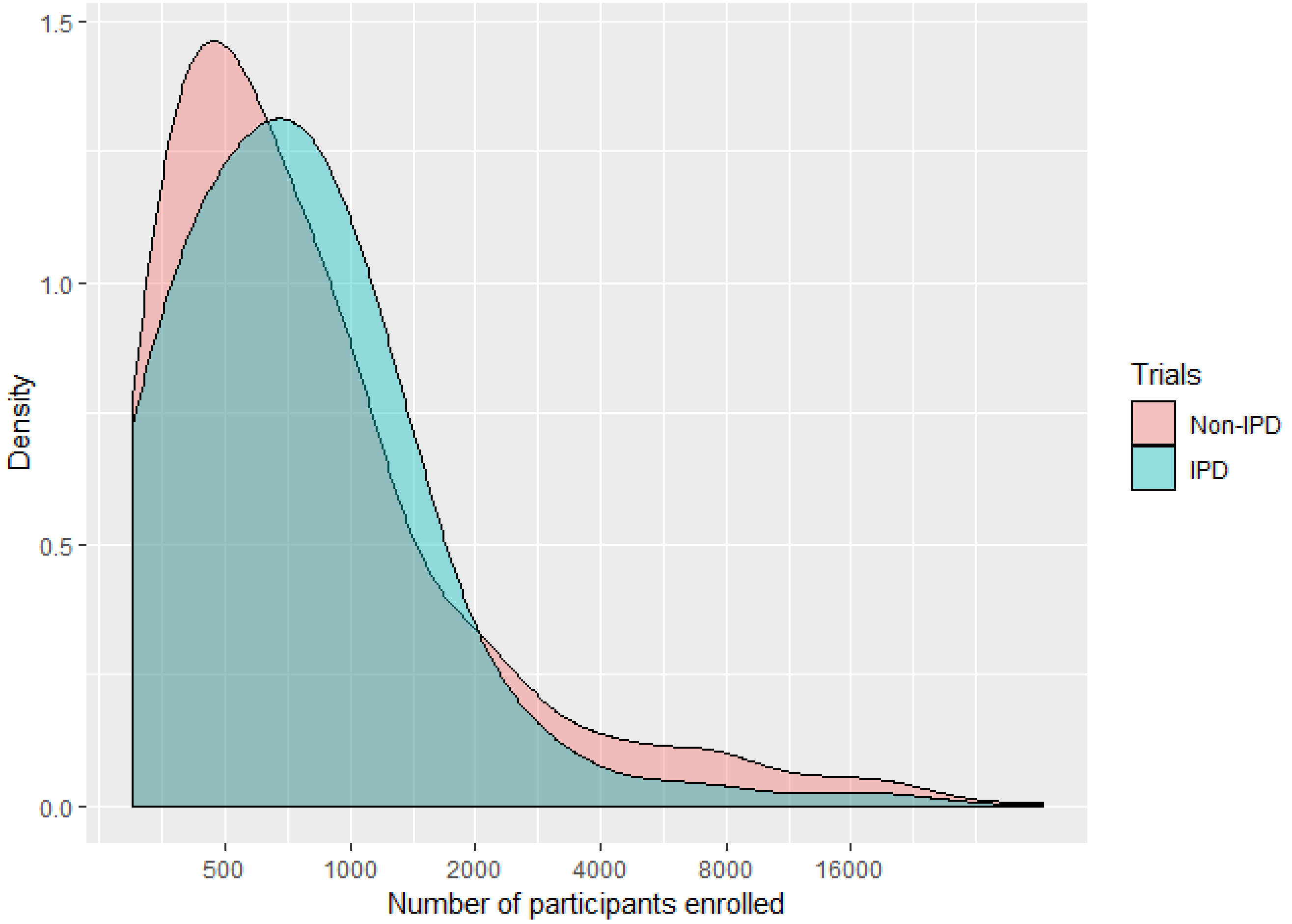

Figure S8.1: Distribution of number of participants enrolled for IPD and non-IPD trials

8.5 Year trial started

Figure S8.2 shows the relationsip between the year the trial was started and whether or not we had access to IPD. The start date was similar for both.

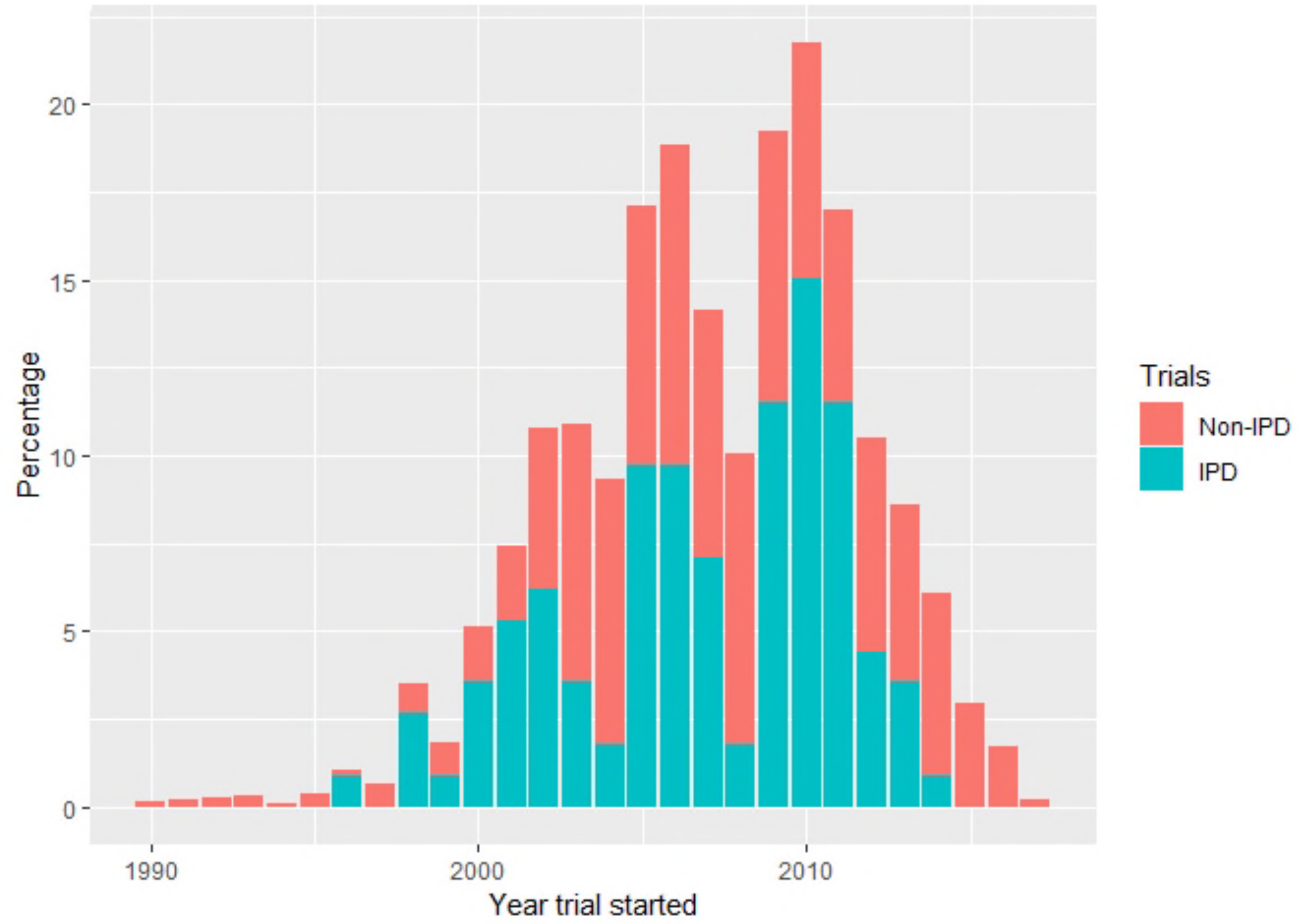

Figure S8.2: Distribution of number of participants enrolled for IPD and non-IPD trials

8.6 Trial eligibility criteria

Table S8.4 shows the number of trials with one or more exclusion for the following groups of conditions. This summary was produced by parsiing the eligibility criterial shown in section Eligibility Criteria using using the EliXR, [7] software to parse the free text and apply the standard vocabulary.
Briefly, the software parses the free-text and maps it across to a standard terminology - the unified medical language systems (UMLS) terminology (which includes Medical Subject Headings (MeSH) as used in Pubmed. We then mapped these terms to their position in the MeSH subject heading tree, then moved further up the MeSH tree to obtain the broader classification within which the terms was situated. For example, if the term “Arthritis, Rheumatoid [C05.799.114]” was identified it was assigned to “Musculoskeletal Diseases [C05]”. MeSH terms can correspond to more than one position on the MeSH tree. Where this was the case, for simplicity, the first term was chosen on the basis of alphabetical/numerical order. The exclusion criteria appear to be broadly similar in the IPD and in the non-IPD trials.

Table S8.4: Comparison of excluded conditions for trials with and without IPD

Exclusion condition - MeSH Term	IPD	No IPD
Number of Trials	113	2054
Bacterial Infections and Mycoses (C01)	28 (24.8%)	269 (13.1%)
Virus Diseases (C02)	6 (5.3%)	103 (5%)
Parasitic Diseases (C03)	3 (2.7%)	14 (0.7%)
Neoplasms (C04)	17 (15%)	325 (15.8%)

Exclusion condition - MeSH Term	IPD	No IPD
Musculoskeletal Diseases (C05)	18 (15.9%)	185 (9%)
Digestive System Diseases (C06)	29 (25.7%)	564 (27.5%)
Stomatognathic Diseases (C07)	1 (0.9%)	34 (1.7%)
Respiratory Tract Diseases (C08)	12 (10.6%)	400 (19.5%)
Otorhinolaryngologic Diseases (C09)	1 (0.9%)	92 (4.5%)
Nervous System Diseases (C10)	26 (23%)	612 (29.8%)
Eye Diseases (C11)	8 (7.1%)	151 (7.4%)
Male Urogenital Diseases (C12)	23 (20.4%)	304 (14.8%)
Female Urogenital Diseases and Pregnancy Complications (C13)	23 (20.4%)	315 (15.3%)
Cardiovascular Diseases (C14)	38 (33.6%)	840 (40.9%)
Hemic and Lymphatic Diseases (C15)	11 (9.7%)	240 (11.7%)
Congenital, Hereditary, and Neonatal Diseases and Abnormalities (C16)	15 (13.3%)	339 (16.5%)
Skin and Connective Tissue Diseases (C17)	30 (26.5%)	224 (10.9%)
Nutritional and Metabolic Diseases (C18)	32 (28.3%)	524 (25.5%)
Endocrine System Diseases (C19)	30 (26.5%)	521 (25.4%)
Immune System Diseases (C20)	50 (44.2%)	752 (36.6%)
Animal Diseases (C22)		7 (0.3%)
Pathological Conditions, Signs and Symptoms (C23)	30 (26.5%)	726 (35.3%)
Occupational Diseases (C24)		3 (0.1%)
Chemically-Induced Disorders (C25)	20 (17.7%)	329 (16%)
Wounds and Injuries (C26)	5 (4.4%)	47 (2.3%)
Behavior and Behavior Mechanisms (F01)	22 (19.5%)	188 (9.2%)
Psychological Phenomena and Processes (F02)		5 (0.2%)
Mental Disorders (F03)	31 (27.4%)	422 (20.5%)

Note - the total number of IPD trials differs slightly across characteristics because of incompleteness in the reporting of these characteristics in clinicaltrials.gov.
