# Supplementary material for: Representation of people with comorbidity and multimorbidity in clinical trials of novel drug therapies: an individual-level participant data analysis
Source: BMC Med. 2019 Nov 12;17:201. doi: 10.1186/s12916-019-1427-1 (PMC6849229; doi:10.1186/s12916-019-1427-1)
Supplement: Supplementary file 9 — Additional file 9. Proportion-with-each-comorbidity-for-trials-and-sail.pdf: Analysis of the prevalence of each comorbidity, within each index condition, in trial participants and the community sample. [file 12916_2019_1427_MOESM9_ESM.pdf]

9 Proportion with each comorbidity for Trials and SAIL

Table S9.1 shows the proportion with each comorbidity. It corresponds to Figure 1 in the main manuscript.

Table S9.1: Proportion of participants/patients with each drug-defined comorbidity

Cohort	Type of condition	nct_id	Indication	Pain	CV	Arthritis	Affective	Acid-related	Airways	Diabetes	Osteoporosis	Thyroid	[illegible]oemboli	Inflammatory	BPH	Gout	Glaucoma	Urological	ED	Antipsychotics	Epilepsy	Migraine	Parkinso	Dementia
TRIAL	Cardiovascular	NCT00262600	Atrial Fibrillation	11.9		11.5	13.7	5.2	9.7	19.7	3.7	9.9		5.8	4.4	6.8	2.7	1.6	1.4	0.8	1.1	0.3	1.3	0.9
SAIL	Cardiovascular		Atrial Fibrillation	53.2		23.3	18.4	14.0	21.0	14.8	9.8	12.5		8.2	9.1	9.2	5.9	4.0	3.1	2.0	1.9	0.7	1.4	0.4
TRIAL	Cardiovascular	NCT00734474	Diabetes Mellitus, Type 2	12.6	64.3	15.9	11.1	11.6	3.7		0.5	9.2	0.3	4.1	2.9	2.6	1.4	0.3	2.7	0.7	1.1	0.4	0.7	0.9
TRIAL	Cardiovascular	NCT00856284	Diabetes Mellitus, Type 2	7.4	31.0	7.4	4.8	4.9	2.6		0.7	2.0	0.4	1.1	1.1	0.8	0.5	0.3	0.8	0.3	0.2	0.1	0.1	0.0
TRIAL	Cardiovascular	NCT00968708	Diabetes Mellitus, Type 2	30.5	93.7	9.5	28.3	1.9	7.4		0.2	2.7	7.6	7.0	3.3	2.7	18.5	0.5	0.6	1.2	0.9	1.2	1.5	0.4
TRIAL	Cardiovascular	NCT00968812	Diabetes Mellitus, Type 2	31.0	22.0	27.0	25.0	32.0	7.0		4.0	15.0	4.0	6.0	10.0	7.0	8.0	1.0	11.0	5.0	8.0	2.0	3.0	1.0
TRIAL	Cardiovascular	NCT01064687	Diabetes Mellitus, Type 2	22.1	74.0	27.8	19.0	11.3	10.6		0.7	9.5	2.1	6.6	3.1	2.1	3.2	1.1	6.7	0.6	1.2	1.6	1.0	0.2
TRIAL	Cardiovascular	NCT01075282	Diabetes Mellitus, Type 2	21.6	66.9	22.2	9.2	7.2	3.3		1.0	7.5	0.8	2.7	1.9	2.3	3.1	0.5	0.8	0.6	0.7	0.1	0.5	0.2
TRIAL	Cardiovascular	NCT01081834	Diabetes Mellitus, Type 2	4.0	27.0	6.0	26.0	28.0	10.0		6.0	13.0	1.0	7.0	12.0	1.0	8.0	6.0	6.0	1.0	2.0	1.0	1.0	1.0
TRIAL	Cardiovascular	NCT01106625	Diabetes Mellitus, Type 2	3.0	16.0	26.0	29.0	24.0	5.0		1.0	12.0	7.0	4.0	3.0	7.0	4.0	1.0	12.0	1.0	1.0	3.0	4.0	1.0
TRIAL	Cardiovascular	NCT01106651	Diabetes Mellitus, Type 2	30.0	23.0	19.0	28.0	26.0	11.0		2.0	16.0	7.0	5.0	15.0	11.0	9.0	7.0	16.0	1.0	9.0	2.0	5.0	1.0
TRIAL	Cardiovascular	NCT01106677	Diabetes Mellitus, Type 2	29.0	20.0	24.0	24.0	22.0	5.0		2.0	10.0	5.0	5.0	6.0	11.0	4.0	4.0	11.0	2.0	10.0	2.0	3.0	1.0
TRIAL	Cardiovascular	NCT01106690	Diabetes Mellitus, Type 2	2.0	21.0	3.0	32.0	14.0	14.0		3.0	22.0	4.0	3.0	13.0	7.0	7.0	4.0	15.0	4.0	3.0	1.0	1.0	1.0

Table S9.1: Proportion of participants/patients with each drug-defined comorbidity

Cohort	Type of condition	nct_id	Indication	Pain	CV	Arthritis	Affective	Acid-related	Airways	Diabetes	Osteoporosis	Thyroid	[illegible]oemboli	Inflammatory	BPH	Gout	Glaucoma	Urological	ED	Antipsychotics	Epilepsy	Migraine	Parkinso	Dement[illegible]a
TRIAL	Cardiovascular	NCT01126580	Diabetes Mellitus, Type 2	18.2	62.7	18.2	12.3	8.1	6.8		1.1	8.4	1.5	2.6	1.2	3.6	2.9	0.9	0.6	1.7	0.6	1.0	1.5	0.2
TRIAL	Cardiovascular	NCT01137812	Diabetes Mellitus, Type 2	29.0	29.0	25.0	27.0	33.0	7.0		2.0	14.0	3.0	2.0	9.0	12.0	5.0	3.0	11.0	6.0	13.0	1.0	6.0	1.0
TRIAL	Cardiovascular	NCT01191268	Diabetes Mellitus, Type 2	25.6	79.7	25.6	16.5	10.9	6.6		0.8	10.3	2.4	3.5	2.4	3.4	5.0	1.0	3.0	1.7	1.6	0.8	0.6	0.2
TRIAL	Cardiovascular	NCT01370005	Diabetes Mellitus, Type 2	15.4		11.8	7.6	8.7	7.0		0.6	9.3	3.5	2.9	2.1	3.8	5.0	1.1	2.2	0.4	0.8	0.4	1.0	0.0
TRIAL	Cardiovascular	NCT01624259	Diabetes Mellitus, Type 2	19.5	69.8	20.9	8.8	7.3	5.7		0.2	7.8	2.0	3.2	2.5	4.7	3.3	0.3	1.3	0.7	0.7	1.3	1.2	0.0
TRIAL	Cardiovascular	NCT01719003	Diabetes Mellitus, Type 2	3.7	17.0	4.1	1.1	2.0	1.8		0.2	0.7	0.0	1.3	0.7	0.6	0.4	0.2	0.4	0.1	0.3	0.1	0.1	0.0
TRIAL	Cardiovascular	NCT01769378	Diabetes Mellitus, Type 2	7.0	65.7	12.0	6.0	4.3	2.7		0.0	6.7	2.7	1.7	2.0	2.7	2.3	0.0	0.0	0.0	0.3	0.3	0.7	0.0
SAIL	Cardiovascular		Diabetes Mellitus, Type 2	50.5	80.9	29.1	23.0	25.0	19.7		4.5	10.8	7.0	8.7	5.9	5.5	4.3	4.0	8.7	2.6	1.8	1.2	1.2	0.2
TRIAL	Cardiovascular	NCT00153023	Diabetes Mellitus, Type 2 (with renal disease)	12.3	0.0	4.4	8.0	9.3	2.7		0.1	4.7	4.1	2.1	15.7	21.2	13.9	0.7	0.8	0.7	1.5	3.6	0.3	1.5
TRIAL	Cardiovascular	NCT00153088	Diabetes Mellitus, Type 2 (with renal disease)	5.5	66.0	21.1	7.2	11.2	3.8		0.6	1.3	0.2	1.7	8.7	6.5	7.4	1.1	2.1	0.8	0.8	0.4	0.8	0.0
TRIAL	Cardiovascular	NCT01164501	Diabetes Mellitus, Type 2 (with renal disease)	4.5	13.5	2.4	0.8	2.6	0.8		0.1	0.1	0.4	0.7	0.8	0.7	0.8	0.1	0.0	0.1	0.0	0.3	0.1	0.0
TRIAL	Cardiovascular	NCT00274599	Hypertension	23.8		28.4	12.4	7.6	9.7	6.8	1.5	3.6	0.1	5.3	2.6	2.5	1.1	0.5	1.4	0.9	1.2	1.4	0.1	0.4
TRIAL	Cardiovascular	NCT00274612	Hypertension	16.4		17.0	10.2	8.2	4.7	3.5	0.4	3.6	0.4	5.5	2.7	3.9	0.9	0.1	0.2	0.4	0.1	1.0	0.1	0.7
TRIAL	Cardiovascular	NCT00926289	Hypertension	7.7		5.6	4.7	3.3	3.3	9.5	0.8	2.1	0.7	0.6	1.3	1.5	2.7	0.3	0.4	0.1	0.2	1.6	0.1	0.2
TRIAL	Cardiovascular	NCT02172586	Hypertension	0.0		3.0	6.6	1.1	0.3	1.4	0.0	1.1	0.0	0.3	3.9	1.1	0.6	0.0	0.0	0.0	0.0	0.0	0.0	0.0
TRIAL	Cardiovascular	NCT02175355	Hypertension	8.8		13.5	7.6	5.9	4.6	5.4	1.3	2.6	0.8	3.8	1.8	1.8	1.3	0.3	0.0	0.5	0.2	0.4	0.4	0.2

Table S9.1: Proportion of participants/patients with each drug-defined comorbidity

Cohort	Type of condition	nct_id	Indication	Pain	CV	Arthritis	Affective	Acid-related	Airways	Diabetes	Osteoporosis	Thyroid	[illegible]oemboli	Inflammatory	BPH	Gout	Glaucoma	Urological	ED	Antipsychotics	Epilepsy	Migraine	Parkinson	Dementia
TRIAL	Cardiovascular	NCT02177396	Hypertension	14.8		18.1	9.4	5.9	4.7	7.5	0.5	3.5	0.0	2.1	4.5	1.2	0.9	0.2	0.5	0.0	0.5	0.2	0.2	1.4
TRIAL	Cardiovascular	NCT02177461	Hypertension	2.4		2.9	6.1	1.9	1.6	1.6	0.5	1.1	0.0	0.3	3.2	1.6	0.5	0.0	0.0	1.3	0.3	0.3	0.5	0.0
TRIAL	Cardiovascular	NCT02242318	Hypertension	10.8		11.0	9.6	4.3	6.5	5.4	0.4	2.7	0.0	4.9	1.1	4.3	0.9	0.0	0.0	0.2	0.4	1.6	0.7	0.2
SAIL	Cardiovascular		Hypertension	46.0		30.2	19.1	22.9	17.9	16.5	6.2	9.8	6.5	7.0	5.0	5.1	4.4	3.4	3.9	1.6	1.7	1.4	0.9	0.2
TRIAL	Cardiovascular	NCT00291330	Thromboembolism	34.5	44.5	25.8	15.4	0.0	9.4	6.9	2.3	6.1		6.4	3.0	2.6	3.0	0.7	0.9	1.7	2.0	0.6	1.2	0.4
TRIAL	Cardiovascular	NCT00329238	Thromboembolism	11.3	38.7	6.1	10.1	5.0	5.5	7.1	1.7	5.1		4.1	3.5	3.6	2.7	0.5	0.9	1.3	1.8	0.6	0.7	0.2
TRIAL	Cardiovascular	NCT00558259	Thromboembolism	7.7	39.2	6.2	9.8	13.0	6.4	6.1	1.9	5.8		4.1	4.2	3.7	2.7	0.6	0.3	1.0	1.6	1.0	0.8	0.6
TRIAL	Cardiovascular	NCT00680186	Thromboembolism	29.0	42.0	23.2	13.6	0.1	8.7	8.8	1.7	5.4		5.4	3.6	2.2	4.0	0.5	1.0	1.0	1.2	0.6	1.3	1.5
SAIL	Cardiovascular		Thromboembolism	61.7	61.6	26.1	23.5	0.0	24.9	12.6	10.8	10.5		11.6	6.2	6.6	4.0	4.6	3.0	2.5	3.3	1.6	1.1	0.3
TRIAL	Inflammatory	NCT00265096	Arthritis, Psoriatic	18.0	13.0		9.0	21.0	25.0	26.0	16.0	27.0	9.0		5.0	14.0	6.0	4.0	7.0	4.0	11.0	3.0	1.0	1.0
TRIAL	Inflammatory	NCT01009086	Arthritis, Psoriatic	32.0	37.0		1.0	23.0	3.0	12.0	9.0	19.0	5.0		4.0	8.0	1.0	1.0	5.0	1.0	4.0	1.0	1.0	1.0
TRIAL	Inflammatory	NCT01077362	Arthritis, Psoriatic	12.0	2.0		4.0	31.0	8.0	18.0	17.0	4.0	7.0		1.0	13.0	7.0	1.0	1.0	7.0	11.0	1.0	1.0	1.0
SAIL	Inflammatory		Arthritis, Psoriatic	55.0	45.4		21.6	15.8	16.5	9.1	5.6	7.8	3.0		2.4	3.7	1.9	2.4	2.4	1.4	1.8	2.4	0.5	0.1
TRIAL	Inflammatory	NCT00106535	Arthritis, Rheumatoid	37.4	32.6		13.4	0.0	4.3	7.3	13.2	12.4	0.9		1.0	0.8	1.7	0.7	0.0	0.1	0.9	0.9	0.7	0.5
TRIAL	Inflammatory	NCT00144508	Arthritis, Rheumatoid	8.2	21.9		6.2	7.5	2.3	5.2	8.5	2.3	0.0		0.7	1.0	0.0	0.7	0.0	0.3	0.3	0.3	0.0	0.0
TRIAL	Inflammatory	NCT00236028	Arthritis, Rheumatoid	16.0	14.0		4.0	34.0	17.0	20.0	16.0	8.0	9.0		9.0	13.0	3.0	3.0	5.0	8.0	8.0	5.0	2.0	1.0
TRIAL	Inflammatory	NCT00264537	Arthritis, Rheumatoid	20.0	12.0		3.0	30.0	22.0	17.0	22.0	32.0	1.0		3.0	9.0	6.0	3.0	3.0	10.0	16.0	2.0	4.0	1.0
TRIAL	Inflammatory	NCT00264550	Arthritis, Rheumatoid	22.0	15.0		6.0	35.0	14.0	17.0	29.0	34.0	11.0		2.0	8.0	8.0	3.0	3.0	3.0	10.0	6.0	3.0	1.0
TRIAL	Inflammatory	NCT00299546	Arthritis, Rheumatoid	28.0	33.0		21.0	5.0	26.0	28.0	15.0	17.0	13.0		7.0	10.0	11.0	10.0	6.0	10.0	14.0	14.0	8.0	1.0
TRIAL	Inflammatory	NCT00361335	Arthritis, Rheumatoid	27.0	19.0		8.0	36.0	20.0	23.0	12.0	9.0	9.0		8.0	6.0	13.0	3.0	3.0	6.0	18.0	8.0	10.0	1.0
TRIAL	Inflammatory	NCT00973479	Arthritis, Rheumatoid	6.0	10.0		19.0	29.0	8.0	11.0	25.0	21.0	5.0		2.0	12.0	3.0	1.0	1.0	3.0	4.0	1.0	1.0	1.0
TRIAL	Inflammatory	NCT01007435	Arthritis,	31.4	23.9		9.0	0.1	5.3	5.5	2.5	9.4	0.8		0.7	1.3	0.7	0.4	0.4	0.2	0.5	0.6	0.1	0.1

Table S9.1: Proportion of participants/patients with each drug-defined comorbidity

Cohort	Type of condition	nct_id	Indication	Pain	CV	Arthritis	Affective	Acid-related	Airways	Diabetes	Osteoporosis	Thyroid	[illegible]oemboli	Inflammatory	BPH	Gout	Glaucoma	Urological	ED	Antipsychotics	Epilepsy	Migraine	Parkinso	Dementia
TRIAL	Inflammatory	NCT01119859	Rheumatoid Arthritis, Rheumatoid	46.9	41.4		19.3	0.0	7.7	10.1	7.7	12.9	1.5		1.8	0.3	0.9	1.5	0.3	0.9	0.6	0.6	0.3	0.3
TRIAL	Inflammatory	NCT01232569	Arthritis, Rheumatoid	32.0	33.1		14.3	12.0	4.4	8.1	11.0	8.5	0.5		0.2	0.2	1.1	0.3	0.2	0.6	0.6	0.5	0.2	0.9
SAIL	Inflammatory		Arthritis, Rheumatoid	66.0	51.9		20.7	22.0	21.3	8.9	20.0	11.7	4.9		2.8	2.5	3.2	3.8	1.6	1.5	1.5	2.4	0.9	0.2
TRIAL	Inflammatory	NCT00036439	Inflammatory bowel disease	17.0	6.0	14.0	7.0	17.0	14.0	21.0	25.0	29.0	1.0		14.0	7.0	9.0	4.0	4.0	1.0	3.0	1.0	1.0	1.0
TRIAL	Inflammatory	NCT00094458	Inflammatory bowel disease	19.0	36.0	2.0	31.0	13.0	1.0	3.0	6.0	5.0	6.0		5.0	3.0	1.0	3.0	3.0	8.0	9.0	1.0	1.0	1.0
TRIAL	Inflammatory	NCT00096655	Inflammatory bowel disease	21.0	3.0	21.0	10.0	8.0	6.0	22.0	26.0	33.0	12.0		14.0	7.0	11.0	4.0	10.0	7.0	3.0	9.0	1.0	1.0
TRIAL	Inflammatory	NCT00207662	Inflammatory bowel disease	25.0	4.0	16.0	16.0	16.0	12.0	5.0	19.0	31.0	9.0		4.0	9.0	5.0	3.0	1.0	7.0	12.0	13.0	1.0	1.0
TRIAL	Inflammatory	NCT00207766	Inflammatory bowel disease	23.0	5.0	23.0	12.0	11.0	21.0	12.0	24.0	18.0	5.0		1.0	10.0	5.0	5.0	1.0	5.0	4.0	8.0	1.0	1.0
TRIAL	Inflammatory	NCT00487539	Inflammatory bowel disease	14.0	9.0	22.0	2.0	18.0	15.0	15.0	13.0	26.0	7.0		11.0	4.0	9.0	2.0	2.0	4.0	10.0	10.0	1.0	1.0
TRIAL	Inflammatory	NCT00488631	Inflammatory bowel disease	10.0	8.0	20.0	2.0	19.0	13.0	16.0	13.0	11.0	8.0		6.0	6.0	8.0	2.0	2.0	5.0	15.0	6.0	1.0	1.0
TRIAL	Inflammatory	NCT00783718	Inflammatory bowel disease	20.8	12.1	4.9	11.3	23.7	2.7	4.2	3.6	5.1	0.7		0.9	0.8	0.4	0.2	0.1	1.0	0.2	0.4	0.9	0.0
TRIAL	Inflammatory	NCT01224171	Inflammatory bowel disease	33.7	10.8	6.0	16.6	33.7	4.1	1.4	4.1	3.4	1.0		0.5	0.5	1.2	0.0	0.2	1.7	1.0	1.9	0.7	0.0
TRIAL	Inflammatory	NCT01369329	Inflammatory bowel disease	15.0	24.0	15.0	4.0	7.0	2.0	2.0	14.0	3.0	4.0		1.0	6.0	3.0	1.0	3.0	8.0	2.0	3.0	1.0	1.0
TRIAL	Inflammatory	NCT01369342	Inflammatory bowel disease	11.0	26.0	18.0	30.0	10.0	5.0	7.0	7.0	1.0	5.0		6.0	9.0	1.0	3.0	3.0	3.0	9.0	4.0	1.0	1.0
TRIAL	Inflammatory	NCT01369355	Inflammatory bowel	13.0	25.0	16.0	34.0	9.0	4.0	4.0	11.0	2.0	5.0		3.0	7.0	2.0	2.0	3.0	6.0	5.0	4.0	1.0	1.0

Table S9.1: Proportion of participants/patients with each drug-defined comorbidity

Cohort	Type of condition	nct_id	Indication	Pain	CV	Arthritis	Affective	Acid-related	Airways	Diabetes	Osteoporosis	Thyroid	[illegible]oemboli	Inflammatory	BPH	Gout	Glaucoma	Urological	ED	Antipsychotics	Epilepsy	Migraine	Parkinso	Dement[illegible]a
			disease																					
SAIL	Inflammatory		Inflammatory bowel disease	40.3	34.6	23.1	20.5	23.5	16.5	6.8	7.7	6.2	3.1		3.0	2.2	2.2	2.2	2.6	1.5	1.5	2.5	0.7	0.1
TRIAL	Inflammatory	NCT00267969	Psoriasis	9.0	17.0	8.0	5.0	25.0	16.0	8.0	3.0	23.0	4.0		13.0	8.0	12.0	1.0	9.0	1.0	17.0	11.0	1.0	1.0
TRIAL	Inflammatory	NCT00307437	Psoriasis	8.0	18.0	7.0	14.0	27.0	19.0	9.0	5.0	28.0	10.0		16.0	17.0	5.0	5.0	8.0	10.0	11.0	5.0	9.0	1.0
TRIAL	Inflammatory	NCT01358578	Psoriasis	2.1	1.3	3.1	0.8	0.7	0.1	0.2	0.0	0.1	0.1		0.1	0.2	0.0	0.0	0.0	0.0	0.0	0.0	0.0	0.0
TRIAL	Inflammatory	NCT01365455	Psoriasis	3.4	2.4	5.7	1.4	0.3	11.5	0.5	0.0	0.1	0.1		0.3	0.3	0.0	0.0	0.0	0.3	0.1	0.3	0.1	0.0
TRIAL	Inflammatory	NCT01474512	Psoriasis	33.6	33.7	36.2	14.4	6.9	8.7	10.8	0.5	7.1	1.2		1.6	1.5	0.8	0.5	0.5	1.5	0.9	0.8	0.8	0.0
TRIAL	Inflammatory	NCT01597245	Psoriasis	37.1	28.1	38.2	14.1	5.6	8.6	9.2	0.3	5.5	1.1		1.4	2.1	0.5	0.4	0.9	2.4	1.3	0.7	0.2	0.0
TRIAL	Inflammatory	NCT01646177	Psoriasis	21.3	29.3	23.9	10.0	6.9	5.6	10.2	0.2	5.6	1.5		1.3	2.5	0.6	0.1	0.4	1.3	1.0	0.5	0.4	0.0
SAIL	Inflammatory		Psoriasis	32.5	35.7	27.1	18.6	14.0	15.0	6.9	3.0	6.0	2.4		2.0	2.1	1.7	1.9	2.0	1.6	1.4	1.9	0.6	0.1
TRIAL	Inflammatory	NCT00410384	Systemic Lupus Erythematosus	69.0	45.0	57.5	34.9	14.6	13.7	5.0	19.6	12.5	10.8		0.2	2.4	2.9	1.3	0.5	1.6	0.0	5.4	1.5	0.1
TRIAL	Inflammatory	NCT00424476	Systemic Lupus Erythematosus	55.9	41.9	46.0	19.1	17.1	7.8	2.8	7.5	8.4	5.2		0.1	2.2	1.7	0.3	0.2	1.5	0.0	1.5	0.1	0.0
SAIL	Inflammatory		Systemic Lupus Erythematosus	57.6	51.9	38.1	28.6	24.5	22.1	5.0	21.2	14.5	9.8		0.8	1.3	2.0	3.8	1.4	1.5	3.5	5.0	0.9	0.3
TRIAL	Musculoskeletal	NCT00265083	Axial Spondyloarthritis	26.0	7.0		11.0	20.0	23.0	13.0	21.0	20.0	7.0		6.0	16.0	10.0	4.0	13.0	7.0	12.0	12.0	4.0	1.0
TRIAL	Musculoskeletal	NCT01209702	Axial Spondyloarthritis	13.7	20.6		12.7	0.0	6.9	2.9	3.9	4.9	0.0		1.0	0.0	2.0	0.0	0.0	1.0	2.0	0.0	0.0	1.0
SAIL	Musculoskeletal		Axial Spondyloarthritis	55.0	41.8		19.4	13.9	14.7	7.1	5.3	4.0	2.8		5.4	2.7	2.2	2.3	3.6	1.1	1.2	1.7	0.4	0.2
TRIAL	Musculoskeletal	NCT02183064	Osteoarthritis		52.2		28.7	24.1	15.1	8.0	6.1	14.1	1.1	8.9	4.8	2.2	3.3	2.0	0.2	0.6	1.4	2.1	1.4	1.9
SAIL	Musculoskeletal		Osteoarthritis		60.3		22.0	23.4	20.1	10.5	7.9	10.7	5.7	7.7	5.1	3.8	4.0	4.5	2.9	1.7	1.8	2.1	1.3	0.3
TRIAL	Musculoskeletal	NCT00046254	Osteoporosis	42.8	58.4	17.8	30.9	15.0	9.9	13.7		8.8	9.9	7.4	1.9	1.7	4.9	2.5	0.1	5.5	2.4	0.7	3.0	4.0
TRIAL	Musculoskeletal	NCT00049829	Osteoporosis	16.2	49.9	24.8	15.4	13.1	6.9	4.9		11.2	1.7	4.7	0.6	0.7	3.5	1.3	0.1	1.0	1.2	1.0	1.1	1.8
TRIAL	Musculoskeletal	NCT00051558	Osteoporosis	30.8	51.7	55.9	21.0	38.7	16.1	5.8		14.0	2.6	90.9	1.9	1.2	1.6	0.5	0.7	1.9	3.5	1.2	0.7	2.1
TRIAL	Musculoskeletal	NCT00100620	Osteoporosis	21.1	47.5	47.3	14.2	32.9	11.0	6.6		5.6	3.7	77.2	2.2	2.5	4.9	0.7	0.2	1.1	1.8	0.8	1.0	1.0
TRIAL	Musculoskeletal	NCT00439244	Osteoporosis	34.0	49.5	30.8	19.7	16.3	10.4	4.9		16.0	2.2	7.0	0.0	1.2	5.3	3.2	0.0	2.9	1.0	2.4	1.5	1.5

Table S9.1: Proportion of participants/patients with each drug-defined comorbidity

Cohort	Type of condition	nct_id	Indication	Pain	CV	Arthritis	Affective	Acid-related	Airways	Diabetes	Osteoporosis	Thyroid	Thromboemboli	Inflammatory	BPH	Gout	Glaucoma	Urological	ED	Antipsychotics	Epilepsy	Migraine	Parkinson	Dementia
TRIAL	Musculoskeletal	NCT00439647	Osteoporosis	15.0	45.1	17.1	10.6	13.5	12.5	7.1		3.2	5.4	7.8	8.8	3.1	3.1	1.3	0.4	1.0	1.4	0.4	1.6	0.4
TRIAL	Musculoskeletal	NCT00670501	Osteoporosis	26.0	47.2	20.2	16.4	77.3	10.2	3.3		15.0	0.1	4.9	1.0	0.3	8.3	0.6	0.1	0.8	1.8	1.3	1.6	0.6
SAIL	Musculoskeletal		Osteoporosis	59.7	57.5	32.4	24.3	30.9	24.1	6.7		14.4	6.5	11.7	2.0	1.9	5.2	5.5	0.8	2.4	2.8	2.0	1.7	0.6
TRIAL	Neurological	NCT00216593	Dementia	24.0	32.0	17.0	33.0	15.0	18.0	27.0	18.0	34.0	15.0	8.0	5.0	14.0	14.0	13.0	1.0		21.0	1.0	11.0	
TRIAL	Neurological	NCT00236431	Dementia	7.0	30.0	9.0	18.0	3.0	21.0	24.0	27.0	6.0	14.0	10.0	18.0	15.0	15.0	12.0	14.0		7.0	7.0	4.0	
TRIAL	Neurological	NCT00236574	Dementia	5.0	31.0	10.0	22.0	6.0	24.0	25.0	28.0	7.0	16.0	9.0	17.0	16.0	16.0	11.0	9.0		5.0	4.0	6.0	
TRIAL	Neurological	NCT00348140	Dementia	30.4	45.6	29.4	31.4	6.9	1.8	2.1	5.5	8.3	2.8	2.1	3.5	2.0	1.8	2.0	0.5		0.0	0.3	1.3	
TRIAL	Neurological	NCT00348309	Dementia	27.7	45.7	25.7	36.3	5.5	1.7	2.1	5.5	7.5	2.7	2.0	4.6	1.9	2.3	3.1	0.1		0.0	0.5	1.9	
TRIAL	Neurological	NCT00423085	Dementia	3.1	46.2	14.2	19.8	19.4	2.4	9.0	1.7	1.0	0.7	3.1	3.8	2.4	2.4	5.2	0.0		2.1	0.0	2.1	
TRIAL	Neurological	NCT00428090	Dementia	18.4	38.2	19.4	16.4	3.1	0.7	2.1	1.9	3.6	0.9	1.2	2.9	0.9	1.2	0.9	0.2		0.0	1.0	0.3	
SAIL	Neurological		Dementia	50.9	50.7	18.3	48.4	27.3	12.0	9.7	14.7	13.0	3.9	7.5	5.4	1.9	6.4	5.3	0.5		3.2	0.4	1.8	
TRIAL	Neurological	NCT00210912	Migraine		11.0	4.0	23.0	12.0	6.0	10.0	8.0	25.0	1.0	11.0	9.0	5.0	1.0	8.0	5.0	5.0	19.0		5.0	1.0
TRIAL	Neurological	NCT00212810	Migraine		35.0	5.0	20.0	4.0	27.0	14.0	10.0	34.0	2.0	9.0	2.0	2.0	2.0	9.0	2.0	9.0	6.0		7.0	1.0
TRIAL	Neurological	NCT00231595	Migraine		1.0	12.0	15.0	1.0	28.0	19.0	23.0	30.0	1.0	11.0	1.0	19.0	1.0	15.0	1.0	1.0	22.0		13.0	1.0
TRIAL	Neurological	NCT00236509	Migraine		34.0	13.0	17.0	2.0	9.0	20.0	20.0	26.0	1.0	12.0	1.0	18.0	1.0	14.0	1.0	1.0	16.0		12.0	1.0
TRIAL	Neurological	NCT00236561	Migraine		28.0	11.0	13.0	26.0	4.0	6.0	1.0	24.0	1.0	1.0	1.0	1.0	12.0	11.0	1.0	11.0	20.0		1.0	1.0
SAIL	Neurological		Migraine		40.8	39.3	28.3	14.5	18.7	2.6	1.9	7.3	0.9	6.1	0.8	0.5	0.8	2.6	0.9	1.7	4.7		0.5	0.0
TRIAL	Neurological	NCT00402233	Parkinson’s disease	15.4	61.7	27.0	22.8	18.6	5.5	15.4	7.7	11.9	2.6	2.9	10.9	3.2	4.8	3.9	2.9	0.6	4.2	2.3		1.0
TRIAL	Neurological	NCT00466167	Parkinson’s disease	3.5	29.5	6.2	22.8	8.9	1.0	6.2	0.6	2.5	0.4	0.8	3.7	0.8	3.7	1.0	0.2	1.0	0.8	0.4		1.0
TRIAL	Neurological	NCT00479401	Parkinson’s disease	4.8	46.2	9.8	17.6	10.8	2.8	8.5	1.5	5.9	0.9	1.7	3.0	1.9	2.8	2.4	0.4	0.4	0.9	0.2		0.7
SAIL	Neurological		Parkinson’s disease	57.0	58.0	25.4	35.3	30.0	14.4	11.0	12.1	10.1	6.2	7.7	11.3	3.0	5.9	9.9	5.1	8.0	6.3	0.6		3.9
TRIAL	Neurological	NCT00133198	Restless legs syndrome	30.4	44.3	44.1	7.8	14.2	9.3	2.0	4.3	18.8	0.9	7.5	2.6	0.9	1.2	0.6	1.4	0.0	0.6	3.2		0.6
TRIAL	Neurological	NCT00472199	Restless legs syndrome	20.2	40.5	21.8	10.0	10.6	12.4	4.5	5.1	8.5	1.8	7.3	5.1	2.1	2.1	0.9	0.6	0.6	1.2	1.5		0.6
SAIL	Neurological		Restless legs syndrome	62.2	57.2	41.6	35.1	27.5	23.2	10.1	8.7	12.9	5.0	9.3	4.5	2.7	3.6	5.6	2.3	2.3	6.1	4.0		0.1
TRIAL	Respiratory	NCT01159912	Asthma	20.3	13.2	17.5	4.3	6.0		4.3	0.6	2.3	0.3	2.9	0.0	0.9	0.6	0.0	0.0	0.9	0.0	1.1	0.0	0.0
TRIAL	Respiratory	NCT01181895	Asthma	10.4	17.3	9.2	3.2	4.0		2.9	0.6	3.7	0.0	1.2	1.2	0.6	0.0	0.6	0.0	0.0	0.0	1.2	0.0	0.0
TRIAL	Respiratory	NCT01436110	Asthma	6.8	7.7	5.7	2.3	3.1		3.4	0.3	1.7	0.0	0.6	0.6	0.0	0.0	0.3	0.0	0.3	0.0	0.0	0.0	0.0

Table S9.1: Proportion of participants/patients with each drug-defined comorbidity

Cohort	Type of condition	nct_id	Indication	Pain	CV	Arthritis	Affective	Acid-related	Airways	Diabetes	Osteoporosis	Thyroid	[illegible]oemboli	Inflammatory	BPH	Gout	Glaucoma	Urological	ED	Antipsychotics	Epilepsy	Migraine	Parkinso	Dementia
TRIAL	Respiratory	NCT01691521	Asthma	13.6	23.8	8.6	11.6	23.6		8.6	7.4	5.5	0.2	23.6	2.6	1.2	1.0	0.7	0.2	1.0	0.5	0.7	0.5	0.0
SAIL	Respiratory		Asthma	39.5	35.5	25.4	21.0	18.3		6.5	4.5	6.4	2.8	6.6	2.4	2.0	1.7	2.3	1.7	1.7	1.7	2.6	0.6	0.1
TRIAL	Respiratory	NCT00125918	Hypertension, Pulmonary	22.2	39.7	13.8	26.8	27.6	17.5	9.4	6.9	19.0		17.7	2.2	7.6	2.7	1.0	0.2	2.0	2.2	2.0	1.0	0.2
SAIL	Respiratory		Hypertension, Pulmonary	57.2	70.9	22.7	21.1	14.6	30.7	11.7	12.1	11.6		9.9	3.2	10.4	3.8	2.4	2.9	1.8	2.6	0.4	1.2	0.4
TRIAL	Respiratory	NCT01316900	Pulmonary Disease, Chronic Obstructive	11.0	48.6	10.5	10.2	8.9		8.0	2.1	7.0	0.8	1.7	5.0	2.4	2.7	0.5	0.9	1.1	0.4	0.9	0.8	0.2
TRIAL	Respiratory	NCT01316913	Pulmonary Disease, Chronic Obstructive	14.0	47.1	15.4	17.4	14.6		11.8	2.9	8.3	1.9	2.2	5.5	2.2	1.6	0.7	1.0	1.7	0.8	0.5	1.1	0.7
TRIAL	Respiratory	NCT01772134	Pulmonary Disease, Chronic Obstructive	19.8	51.4	15.2	16.7	14.1		14.3	2.6	11.7	2.4	1.6	6.6	4.5	2.1	1.0	1.0	2.6	0.6	0.6	1.0	0.2
TRIAL	Respiratory	NCT01957163	Pulmonary Disease, Chronic Obstructive	18.4	54.6	15.7	21.2	12.4		12.9	2.1	7.9	2.4	1.1	4.7	1.1	1.6	0.6	1.5	2.6	0.8	0.5	1.3	0.6
TRIAL	Respiratory	NCT02119286	Pulmonary Disease, Chronic Obstructive	14.0	52.4	15.6	16.5	12.9		15.3	2.4	9.5	3.1	1.6	6.0	5.2	1.0	1.1	1.9	1.5	1.0	0.6	1.5	0.3
TRIAL	Respiratory	NCT02177344	Pulmonary Disease, Chronic Obstructive	24.8	30.5	11.3	23.1	17.0		4.3	3.1	8.2	1.2	19.7	2.0	1.9	1.1	0.5	0.2	1.2	1.4	0.5	0.2	0.6
TRIAL	Respiratory	NCT02236611	Pulmonary Disease, Chronic Obstructive	8.5	59.1	8.0	9.9	9.3		12.5	1.1	6.3	2.9	0.6	2.2	2.8	0.9	0.4	0.4	1.3	0.9	0.1	0.6	0.1
SAIL	Respiratory		Pulmonary Disease, Chronic Obstructive	58.4	61.2	29.1	26.8	30.3		12.5	11.4	10.0	8.0	10.7	7.1	4.2	4.1	3.8	3.2	2.6	2.3	1.5	1.2	0.3
TRIAL	Respiratory	NCT01335464	Pulmonary fibrosis	21.6	50.8	15.2	14.6	30.9	18.5	16.7	6.0	9.3	1.0	0.0	8.4	5.8	3.7	1.2	2.3	0.8	2.1	0.2	0.6	0.2
TRIAL	Respiratory	NCT01335477	Pulmonary fibrosis	19.9	48.3	15.5	14.8	29.5	24.0	19.3	4.6	9.1	0.2	0.0	6.9	4.4	3.5	1.1	3.1	2.2	0.7	0.2	1.6	1.1
SAIL	Respiratory		Pulmonary fibrosis	60.5	66.2	30.8	22.0	38.6	44.8	14.0	25.7	12.4	12.6	0.0	9.8	6.3	4.9	4.2	3.2	1.8	1.8	0.8	1.5	0.2
TRIAL	Urological	NCT00384930	Benign Prostatic Hyperplasia	12.4	44.5	24.8	8.2	9.6	5.2	7.7	0.2	4.0	0.7	6.1		1.9	2.8		32.1	0.5	0.7	0.9	0.9	0.0
TRIAL	Urological	NCT00827242	Benign Prostatic Hyperplasia	2.2	4.3	2.8	0.3	1.8	1.2	2.5	0.3	0.0	1.5	1.5		0.0	0.3		15.7	0.3	0.3	0.0	0.0	0.0
TRIAL	Urological	NCT00848081	Benign Prostatic	13.5	86.5	27.0	11.9	16.0	8.8	14.2	0.9	11.3	3.1	6.3		6.3	4.7		30.8	0.3	0.9	1.9	0.6	2.2

Table S9.1: Proportion of participants/patients with each drug-defined comorbidity

Cohort	Type of condition	nct_id	Indication	Pain	CV	Arthritis	Affective	Acid-related	Airways	Diabetes	Osteoporosis	Thyroid	[illegible]oemboli	Inflammatory	BPH	Gout	Glaucoma	Urological	ED	Antipsychotics	Epilepsy	Migraine	Parkinso	Demen[illegible]
TRIAL	Urological	NCT00861757	Benign Prostatic Hyperplasia	3.0	53.3	11.3	5.7	10.3	4.5	9.2	0.0	1.9	0.0	3.8		5.4	1.4		0.0	0.6	0.0	0.0	0.0	0.5
TRIAL	Urological	NCT00970632	Benign Prostatic Hyperplasia	2.5	50.9	4.3	1.4	5.7	2.3	11.4	0.6	2.3	2.0	2.2		4.5	0.8		12.7	0.2	0.2	0.2	0.0	0.6
SAIL	Urological		Benign Prostatic Hyperplasia	43.3	64.1	29.5	15.1	25.2	18.9	12.5	3.5	4.6	8.5	7.1		5.9	5.5		8.3	1.6	2.1	0.9	1.9	0.4
TRIAL	Urological	NCT00855582	Erectile dysfunction	7.8	44.1	12.4	5.4	7.8	5.1	11.4	1.2	5.1	0.8	2.6		2.5	2.6			0.5	0.3	0.8	0.5	0.8
SAIL	Urological		Erectile dysfunction	37.3	56.5	28.9	18.3	19.9	15.8	20.2	1.7	3.5	4.9	6.2		5.3	2.8			1.9	1.8	0.9	1.1	0.1
