# Supplementary material for: Representation of people with comorbidity and multimorbidity in clinical trials of novel drug therapies: an individual-level participant data analysis
Source: BMC Med. 2019 Nov 12;17:201. doi: 10.1186/s12916-019-1427-1 (PMC6849229; doi:10.1186/s12916-019-1427-1)
Supplement: Supplementary file 10 — Additional file 10. Comorbidity-counts-for-trials-and-primary-care.pdf: Summary comorbidity counts. [file 12916_2019_1427_MOESM10_ESM.zip › 10-comorbidity-counts-for-trials-and-primary-careR2.pdf]

10 Comorbidity counts for trials and primary care

The counts for Trials are shown in S10.1.

10.1 Unstandardised counts

Table S10.1: Count of number of participants with each comorbidity count in trials

nct_id	00	01	02	03	04	05	06	07	08	09	10	condition_grp
NCT00036439	145	107	68	32	6	5	1	0	0	0	0	Inflammatory_bowel_disease
NCT00046254	272	483	515	391	272	111	62	17	4	0	0	Osteoporosis
NCT00049829	1708	2469	2065	1033	349	109	23	8	0	0	0	Osteoporosis
NCT00051558	1	68	148	109	53	38	10	2	0	0	0	Osteoporosis
NCT00094458	224	209	56	17	0	1	0	0	0	0	0	Inflammatory_bowel_disease
NCT00096655	145	109	65	27	8	8	1	0	0	0	0	Inflammatory_bowel_disease
NCT00100620	5	230	274	185	82	35	15	5	0	2	0	Osteoporosis
NCT00106535	388	356	208	123	52	15	3	2	1	0	0	rheumatoid_arthritis
NCT00125918	89	83	87	69	37	20	16	3	2	0	0	Hypertension_Pulmonary
NCT00133198	69	75	113	61	20	5	2	0	0	0	0	Restless_Legs_Syndrome
NCT00144508	156	109	33	8	0	0	0	0	0	0	0	rheumatoid_arthritis
NCT00152971	371	535	454	452	410	205	106	47	16	3	0	Arthroplasty_Replacement_Knee
NCT00153023	358	274	161	62	26	4	0	0	0	0	0	Diabetes_Mellitus_Type_2_plus_renal
NCT00153088	110	197	133	63	17	5	2	0	0	0	0	Diabetes_Mellitus_Type_2_plus_renal

NCT00168818	1922	523	481	305	166	52	7	3	0	0	0	Arthroplasty_Replacement_Hip
NCT00207662	180	216	117	46	17	3	1	0	0	0	0	Inflammatory_bowel_disease
NCT00207766	92	127	64	19	3	0	1	0	0	0	0	Inflammatory_bowel_disease
NCT00210912	108	122	74	20	4	0	0	0	0	0	0	Migraine
NCT00212810	165	148	56	10	5	0	0	0	0	0	0	Migraine
NCT00216593	23	81	134	114	42	15	6	0	0	0	0	Alzheimers_Disease
NCT00231595	347	306	65	12	0	0	0	0	0	25	12	Migraine
NCT00236028	214	225	101	50	13	2	1	0	0	0	0	rheumatoid_arthritis
NCT00236431	153	420	271	138	61	15	4	0	0	0	0	Alzheimers_Disease
NCT00236509	337	298	74	14	5	0	0	0	0	30	4	Migraine
NCT00236561	209	498	79	0	0	0	0	0	0	0	0	Migraine
NCT00236574	105	359	270	160	59	17	3	1	0	0	0	Alzheimers_Disease
NCT00262600	7173	5615	3097	1431	562	163	54	18	0	0	0	Atrial_Fibrillation
NCT00264537	241	217	111	56	8	3	1	0	0	0	0	rheumatoid_arthritis
NCT00264550	153	157	76	34	17	7	0	0	0	0	0	rheumatoid_arthritis
NCT00265083	146	120	54	25	7	2	1	0	0	0	0	Axial_Spondyloarthritis
NCT00265096	151	142	74	26	8	3	1	1	0	0	0	Psoriatic_arthritis
NCT00267969	213	309	175	58	10	0	0	0	0	0	0	Psoriasis
NCT00274599	288	321	152	38	11	1	1	0	0	0	0	Hypertension
NCT00274612	392	271	107	24	6	1	0	0	0	0	0	Hypertension
NCT00289198	181	82	26	9	3	1	0	0	0	0	0	Rhinitis_Allergic_Perennial
NCT00291330	613	764	672	313	131	51	13	5	3	0	0	Thromboembolism

NCT00299546	58	109	129	85	43	23	12	2	0	0	0	rheumatoid_arthritis
NCT00307437	315	514	244	105	44	5	2	0	0	0	0	Psoriasis
NCT00329238	1212	805	479	226	92	43	9	3	0	0	0	Thromboembolism
NCT00348140	359	431	386	214	79	16	0	0	0	0	0	Alzheimers_Disease
NCT00348309	361	414	417	206	73	22	3	0	0	0	0	Alzheimers_Disease
NCT00361335	165	224	131	68	36	16	3	0	0	0	0	rheumatoid_arthritis
NCT00384930	101	112	122	68	13	9	1	1	0	0	0	Benign_Prostatic_Hyperplasia
NCT00402233	45	66	83	58	42	10	6	1	0	0	0	Parkinson_Disease
NCT00410384	23	194	245	173	104	61	19	4	3	0	0	Systemic_Lupus_Erythematosus
NCT00423085	91	82	60	37	14	4	0	0	0	0	0	Alzheimers_Disease
NCT00424476	86	274	261	176	46	19	4	1	0	0	0	Systemic_Lupus_Erythematosus
NCT00428090	261	161	102	44	10	2	1	0	0	0	0	Alzheimers_Disease
NCT00439244	66	107	118	67	35	11	6	2	0	0	0	Osteoporosis
NCT00439647	309	353	277	165	68	20	5	1	1	0	0	Osteoporosis
NCT00466167	233	155	82	32	13	2	1	0	0	0	0	Parkinson_Disease
NCT00472199	81	111	76	43	16	1	2	0	0	1	0	Restless_Legs_Syndrome
NCT00479401	187	164	117	40	24	4	2	1	0	0	0	Parkinson_Disease
NCT00487539	428	357	169	81	22	6	1	0	0	0	0	Inflammatory_bowel_disease
NCT00488631	513	403	204	85	18	4	1	0	0	0	0	Inflammatory_bowel_disease
NCT00558259	584	329	229	129	51	22	8	1	0	0	0	Thromboembolism
NCT00657150	865	265	304	312	175	92	33	6	2	0	0	Arthroplasty_Replacement_Hip
NCT00670501	3	528	565	383	175	55	21	2	2	0	0	Osteoporosis

NCT00680186	676	782	681	308	88	39	18	0	0	0	0	Thromboembolism
NCT00694382	3207	6	0	0	0	0	0	0	0	0	0	Solid_tumor
NCT00734474	282	440	292	118	47	18	4	0	0	0	0	Diabetes_Mellitus_Type_2
NCT00783718	414	267	130	59	19	4	2	0	0	0	0	Inflammatory_bowel_disease
NCT00827242	233	74	14	4	0	0	0	0	0	0	0	Benign_Prostatic_Hyperplasia
NCT00848081	19	63	94	95	22	21	3	1	0	0	0	Benign_Prostatic_Hyperplasia
NCT00855582	236	175	137	43	8	4	3	0	0	0	0	Erectile_Dysfunction_Benign_Prostatic_Hyperplasia
NCT00856284	1470	808	248	79	26	5	0	0	0	0	0	Diabetes_Mellitus_Type_2
NCT00861757	215	235	110	52	12	5	0	0	0	0	0	Benign_Prostatic_Hyperplasia
NCT00926289	642	162	53	25	9	0	1	0	0	0	0	Hypertension
NCT00968708	249	1796	1585	1006	503	179	70	22	0	0	0	Diabetes_Mellitus_Type_2
NCT00968812	659	552	179	44	16	3	0	0	0	0	0	Diabetes_Mellitus_Type_2
NCT00970632	203	152	107	34	14	1	0	0	0	0	0	Benign_Prostatic_Hyperplasia
NCT00973479	334	178	58	17	6	0	0	0	0	0	0	rheumatoid_arthritis
NCT01007435	655	399	208	90	29	17	1	0	0	0	0	rheumatoid_arthritis
NCT01009086	453	126	30	2	2	0	2	0	0	0	0	Psoriatic_arthritis
NCT01064687	125	290	267	190	73	26	6	1	0	0	0	Diabetes_Mellitus_Type_2
NCT01075282	179	328	217	75	17	12	1	0	0	0	0	Diabetes_Mellitus_Type_2
NCT01077362	173	84	40	8	4	4	0	0	0	0	0	Psoriatic_arthritis
NCT01081834	222	334	106	14	1	0	0	0	0	0	0	Diabetes_Mellitus_Type_2
NCT01106625	228	182	39	12	6	1	0	0	0	0	0	Diabetes_Mellitus_Type_2
NCT01106651	329	254	97	28	4	3	0	0	0	0	0	Diabetes_Mellitus_Type_2

NCT01106677	641	473	126	36	8	3	0	0	0	0	0	Diabetes_Mellitus_Type_2
NCT01106690	149	127	57	11	1	0	0	0	0	0	0	Diabetes_Mellitus_Type_2
NCT01119859	64	118	78	45	12	7	2	0	0	0	0	rheumatoid_arthritis
NCT01126580	192	292	184	95	30	9	4	1	0	0	0	Diabetes_Mellitus_Type_2
NCT01137812	307	311	93	39	3	2	2	0	0	0	0	Diabetes_Mellitus_Type_2
NCT01159912	204	102	32	7	4	0	0	0	0	0	0	Asthma
NCT01164501	587	109	39	1	5	0	0	0	0	0	0	Diabetes_Mellitus_Type_2_plus_renal
NCT01181895	231	85	21	6	3	1	0	0	0	0	0	Asthma
NCT01191268	103	292	274	142	47	23	8	2	1	0	0	Diabetes_Mellitus_Type_2
NCT01209702	58	24	13	5	1	1	0	0	0	0	0	Axial_Spondyloarthritis
NCT01224171	134	153	75	34	14	3	2	1	0	0	0	Inflammatory_bowel_disease
NCT01232569	205	210	135	75	21	9	1	0	0	0	0	rheumatoid_arthritis
NCT01264939	4	80	123	79	25	20	4	1	0	0	0	Chronic_Idiopathic_Urticaria_(CIU)
NCT01287117	117	89	62	30	14	3	2	2	0	0	0	Chronic_Idiopathic_Urticaria_(CIU)
NCT01292473	117	95	54	38	13	5	1	0	0	0	0	Chronic_Idiopathic_Urticaria_(CIU)
NCT01316900	316	262	143	70	38	12	5	0	0	0	0	Pulmonary_Disease_Chronic_Obstructive
NCT01316913	240	266	189	103	55	17	1	0	1	0	0	Pulmonary_Disease_Chronic_Obstructive
NCT01335464	98	105	123	98	55	25	9	0	1	0	0	Pulmonary_Fibrosis
NCT01335477	100	123	134	101	49	26	11	4	1	0	0	Pulmonary_Fibrosis
NCT01358578	1211	82	13	0	0	0	0	0	0	0	0	Psoriasis
NCT01365455	582	132	23	0	1	0	0	0	0	0	0	Psoriasis
NCT01369329	375	295	69	25	5	0	0	0	0	0	0	Inflammatory_bowel_disease

NCT01369342	342	224	56	14	4	1	0	0	0	0	0	Inflammatory_bowel_disease
NCT01369355	651	467	119	36	8	1	0	0	0	0	0	Inflammatory_bowel_disease
NCT01370005	389	273	122	32	7	2	0	0	0	0	0	Diabetes_Mellitus_Type_2
NCT01436110	278	54	15	3	1	0	0	0	0	0	0	Asthma
NCT01474512	327	442	295	137	64	19	10	1	0	0	0	Psoriasis
NCT01597245	300	488	244	114	50	20	9	0	0	0	0	Psoriasis
NCT01624259	126	211	161	73	18	6	3	1	0	0	0	Diabetes_Mellitus_Type_2
NCT01646177	510	446	202	118	48	16	1	1	1	0	0	Psoriasis
NCT01691521	198	176	107	59	25	8	5	2	0	0	0	Asthma
NCT01719003	1046	282	72	11	1	1	0	0	0	0	0	Diabetes_Mellitus_Type_2
NCT01769378	84	131	62	14	7	2	0	0	0	0	0	Diabetes_Mellitus_Type_2
NCT01772134	145	176	151	78	46	16	3	1	1	0	0	Pulmonary_Disease_Chronic_Obstructive
NCT01957163	146	183	155	83	34	13	5	0	0	0	0	Pulmonary_Disease_Chronic_Obstructive
NCT02119286	137	194	151	85	35	13	4	1	0	0	0	Pulmonary_Disease_Chronic_Obstructive
NCT02172586	298	59	4	1	0	0	0	0	0	0	0	Hypertension
NCT02175355	627	277	108	22	4	1	0	0	0	0	0	Hypertension
NCT02177344	144	229	160	69	32	10	2	0	0	0	0	Pulmonary_Disease_Chronic_Obstructive
NCT02177396	211	150	45	18	0	1	1	0	0	0	0	Hypertension
NCT02177461	292	70	11	1	0	0	0	0	0	0	0	Hypertension
NCT02183064	230	384	336	240	91	30	7	3	1	0	0	Osteoarthritis
NCT02236611	290	389	228	89	33	5	2	1	0	0	0	Pulmonary_Disease_Chronic_Obstructive
NCT02242318	268	111	46	20	0	1	0	0	0	0	0	Hypertension

10.2 Percentage of patients/participants with given comorbidity counts

The proportion with each count (or in the case of primary care) standardised count is shown in Table S10.2. This corresponds directly to the results shown in Figure 2 of the main manuscript. It has been rounded to 1 decimal place, and cells with <= 0.05% have been left blank to ease readability.

Table S10.2: Proportion with each comorbidity count for trialsand Primary Care

Indication	Population	0	1	2	3	4	5	6	7	8	9	10	11	12
Atrial Fibrillation	Primary (stnd. trials)	13.7	22.9	24.4	19	11.3	5.6	2.2	0.7	0.2				
Atrial Fibrillation	Trials	39.6	31	17.1	7.9	3.1	0.9	0.3	0.1					
Diabetes Mellitus, Type 2	Primary (stnd. trials)	5.6	16.4	23.1	22.3	16.5	9.5	4.4	1.6	0.5	0.1			
Diabetes Mellitus, Type 2	Trials	36.8	36.8	18.4	6.1	1.5	0.3	0.1						
Diabetes Mellitus, Type 2 (renal)	Primary (stnd. trials)	4.2	15.4	23.1	23	17.3	10	4.6	1.7	0.5	0.1			
Diabetes Mellitus, Type 2 (renal)	Trials	47	35.5	13.4	3.4	0.6	0.1							
Hypertension	Primary (stnd. trials)	18.7	27.2	23.4	15.6	8.8	4	1.6	0.5	0.1				
Hypertension	Trials	60.8	30.3	7.5	1.3	0.2								
Thromboembolism	Primary (stnd. trials)	12.5	19.2	21.6	20.4	12.8	8	3.7	1.2	0.4	0.2			
Thromboembolism	Trials	27.4	35.5	23	9.9	3.2	0.8	0.2						
Axial Spondyloarthritis	Primary (stnd. trials)	32.1	28.6	22.6	10.6	3.7	1.6	0.6	0.2					
Axial Spondyloarthritis	Trials	43.3	36.2	15.2	4.2	0.9	0.1							

Inflammatory bowel disease	Primary (stnd. trials)	32.1	24.5	19.4	12.7	6.6	3	1.2	0.4	0.1		
Inflammatory bowel disease	Trials	40.4	36.6	16.6	5	1.1	0.2					
Psoriasis	Primary (stnd. trials)	37.6	22.9	19.7	11.1	5.4	2.2	0.8	0.2			
Psoriasis	Trials	50.7	34.4	11.7	2.7	0.5	0.1					
Psoriatic arthropathy	Primary (stnd. trials)	21.7	27.3	23.2	13.5	8.7	3.3	1.8	0.4	0.2		
Psoriatic arthropathy	Trials	52.6	33.8	10.8	2.3	0.4						
Rheumatoid arthritis	Primary (stnd. trials)	17.1	24.2	24.1	17.3	9.5	4.8	2	0.8	0.1		
Rheumatoid arthritis	Trials	32.3	36.5	20.6	7.8	2.2	0.5	0.1				
Systemic Lupus Erythematosus	Primary (stnd. trials)	16.5	15.2	24.2	19.6	11.2	9.3	2.8	1.2		0.1	
Systemic Lupus Erythematosus	Trials	11.4	24.7	26.9	19.5	10.6	4.6	1.7	0.5			
Hip arthroplasty	Primary (stnd. trials)	8.3	17.7	28.8	21.7	13.9	5.6	2.6	0.8	0.4	0.1	
Hip arthroplasty	Trials	28.4	35.8	22.5	9.4	3	0.7	0.2				
Knee arthroplasty	Primary (stnd. trials)	4.6	13.7	27.2	23.5	16.8	8.9	3.5	1.3	0.3		
Knee arthroplasty	Trials	14.3	20.6	17.5	17.4	15.8	7.9	4.1	1.8	0.6	0.1	
Osteoarthritis	Primary (stnd. trials)	15.8	23.1	23.9	17.9	10.9	5.3	2.1	0.7	0.2		
Osteoarthritis	Trials	17.4	29	25.4	18.2	6.9	2.3	0.5	0.2	0.1		
Osteoporosis	Primary (stnd.	8.1	12.8	19.9	21.5	17.8	11.3	5.7	2.1	0.6	0.2	0.1

	trials)											
Osteoporosis	Trials	13	26.5	27.1	18.4	9.4	3.8	1.3	0.4	0.1		
Alzheimerâ€™s Disease	Primary (stnd. trials)	10.1	12.6	18.7	20.1	15.3	12.5	7.2	2.7	0.8	0.1	
Alzheimerâ€™s Disease	Trials	21.5	33	25.4	13	5	1.5	0.4	0.1			
Dementia (any)	Primary (stnd. trials)	7.9	9.6	15.8	19.2	17.7	14.5	8.7	4.6	1.4	0.4	0.1
Dementia (any)	Trials	21.5	33	25.4	13	5	1.5	0.4	0.1			
Migraine	Primary (stnd. trials)	0.9	29	32.1	21.4	10.5	3.9	1.5	0.5	0.1		
Migraine	Trials	38.5	36.7	17.5	5.6	1.3	0.3					
Parkinsonâ€™s disease (all)	Primary (stnd. trials)	11.3	13.9	18.1	20.5	14.9	11	6	3	0.9	0.3	0.1
Parkinsonâ€™s disease (all)	Trials	26.9	35.3	23.2	10.2	3.3	0.9	0.2				
Parkinsonâ€™s disease (excluding secondary)	Primary (stnd. trials)	12.1	14.7	18.2	20.5	14.8	10.2	5.3	2.9	0.8	0.3	0.1
Parkinsonâ€™s disease (excluding secondary)	Trials	26.9	35.3	23.2	10.2	3.3	0.9	0.2				
Restless legs syndrome	Primary (stnd. trials)	12.3	14.1	19.4	19.4	15.5	10.3	5.2	2.6	0.8	0.3	0.1
Restless legs syndrome	Trials	20.4	32.4	25.8	13.7	5.4	1.7	0.5	0.1			
Asthma	Primary (stnd. trials)	26.1	23.4	20.3	14.5	8.7	4.3	1.8	0.6	0.2		
Asthma	Trials	57	32.1	9	1.7	0.2						
Hypertension, Pulmonary	Primary (stnd.	6.1	12.8	20	20.7	19.5	11.9	3.3	2.8	0.3	0.9	

	trials)										
Hypertension, Pulmonary	Trials	21.9	20.4	21.4	17	9.1	4.9	3.9	0.7	0.5	
Pulmonary Disease, Chronic Obstructive	Primary (stnd. trials)	9.8	15.4	21.3	21.4	16.2	9.3	4.5	1.6	0.4	0.1
Pulmonary Disease, Chronic Obstructive	Trials	23.8	34.1	24.5	11.8	4.2	1.2	0.3	0.1		
Pulmonary fibrosis	Primary (stnd. trials)	4.2	7.6	14.8	22.7	21.5	15.7	8.2	4.4	0.6	0.3
Pulmonary fibrosis	Trials	12.9	26.4	27	18.5	9.5	3.9	1.3	0.4	0.1	
Benign Prostatic Hyperplasia	Primary (stnd. trials)	14.4	19.2	23.7	19.3	11.8	6.8	3.3	1.1	0.3	0.1
Benign Prostatic Hyperplasia	Trials	33.7	36.7	19.9	7.2	2	0.4	0.1			
Erectile dysfunction	Primary (stnd. trials)	13.3	17.6	23.6	20.4	13.5	7.1	2.9	1.1	0.3	0.1
Erectile dysfunction	Trials	38.9	28.9	22.6	7.1	1.3	0.7	0.5			

**stnd. trials refers to proportions which have been directly age-sex standardised to trials.
