# Supplementary material for: Representation of people with comorbidity and multimorbidity in clinical trials of novel drug therapies: an individual-level participant data analysis
Source: BMC Med. 2019 Nov 12;17:201. doi: 10.1186/s12916-019-1427-1 (PMC6849229; doi:10.1186/s12916-019-1427-1)
Supplement: Supplementary file 10 — Additional file 10. Comorbidity-counts-for-trials-and-primary-care.pdf: Summary comorbidity counts. [file 12916_2019_1427_MOESM10_ESM.zip › 10-comorbidity-counts-for-trials-and-primary-care.2R2.pdf]

10.3 Unstandardised and standardised co[illegible] compared to trials

Figure 10.1 adds the distribution for comorbidity counts unstandardised, to the co[illegible] distributions of comorbidity counts standardised by age and sex adn trials shown [illegible] in the main manuscript.

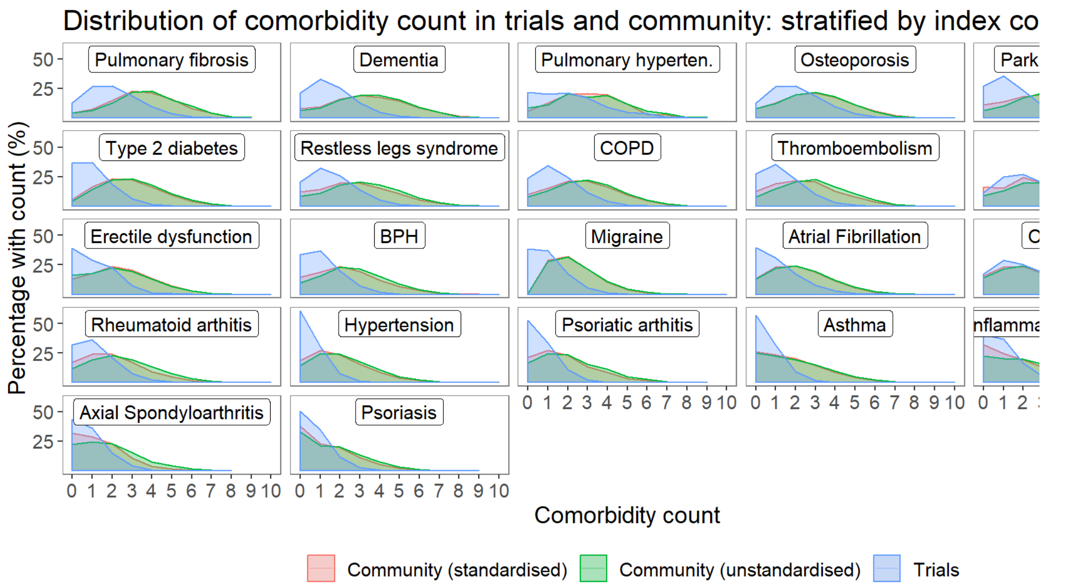

Figure S10.1: Comorbidity count comparisons

Previous Next
