# Supplementary material for: Representation of people with comorbidity and multimorbidity in clinical trials of novel drug therapies: an individual-level participant data analysis
Source: BMC Med. 2019 Nov 12;17:201. doi: 10.1186/s12916-019-1427-1 (PMC6849229; doi:10.1186/s12916-019-1427-1)
Supplement: Supplementary file 11 — Additional file 11. Explore-relationship-of-trial-mean-comorbidity-counts-to-trial-characteristics.pdf: Analysis of characteristics based on trial meta-data and relationship to comorbidity counts. [file 12916_2019_1427_MOESM11_ESM.pdf]

11 Explore relationship of trial mean comorbidity counts to trial characteristics

We noticed that the ratio between trial and primary care comorbidity counts showed substantial variation. This was true even within the same indication. We therefore performed an exploratory analysis to identify if any trial-characteristics were associated with the trial comorbidity count.

As the following plots show, there was no visual evidence of associations between the comorbidity count and any of the following characteristics : -

- Start date of the trial
- number of trial participants
- sponsor
- drug class
- number of conditions featured in the inclusion criteria
- number of conditions featured in the exclusion criteria

The number of conditions was defined using the MeSH tree as per section Trial eligibility criteria section.

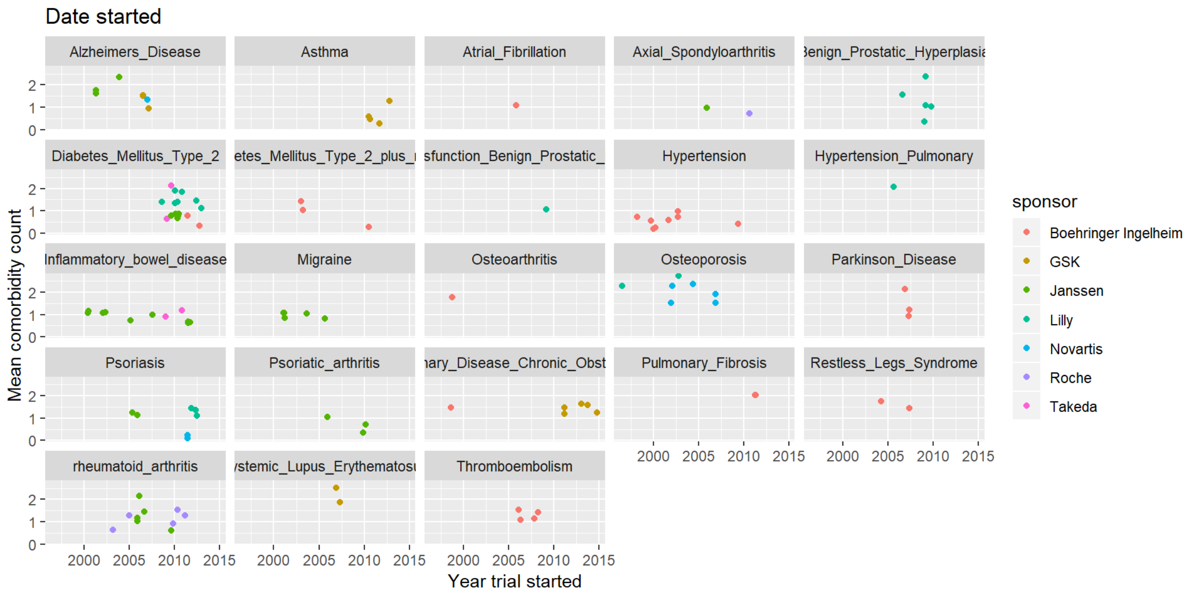

Figure S11.1: Sponsors and number of participants

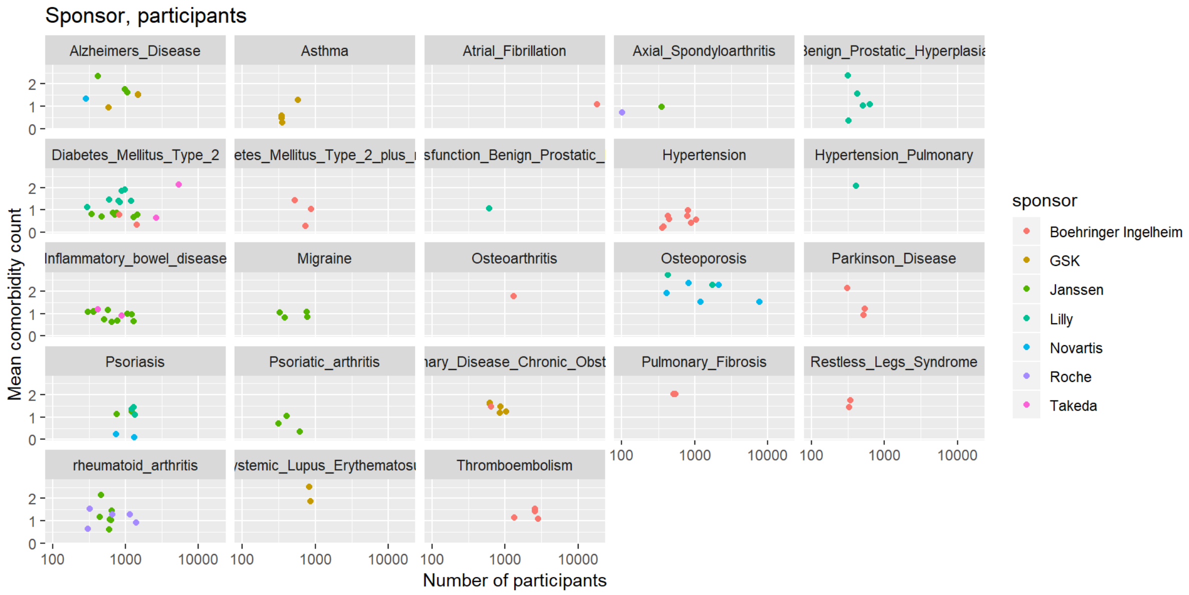

Figure S11.2: Sponsors and number of participants

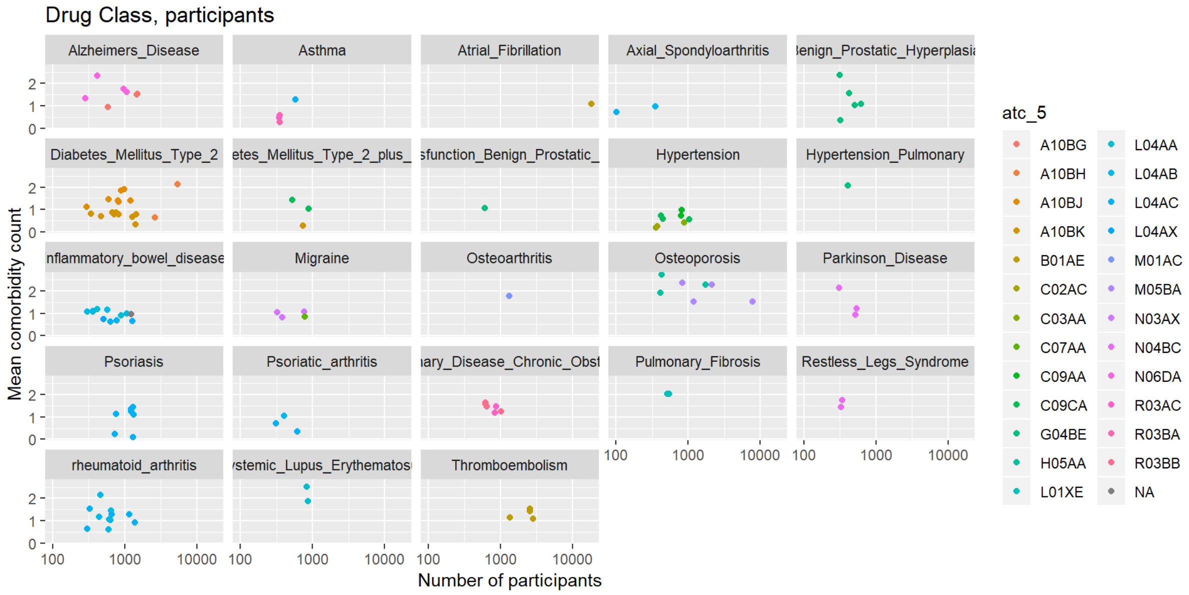

Figure S11.3: Drug class and number of participants

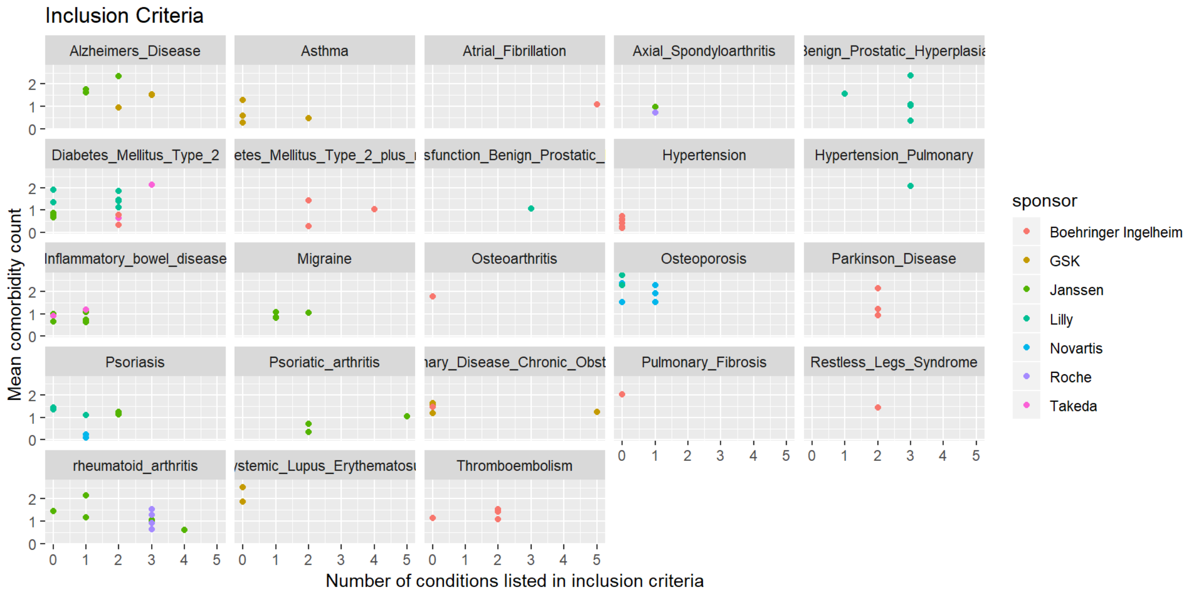

Figure S11.4: Inclusion Criteria and number of participants

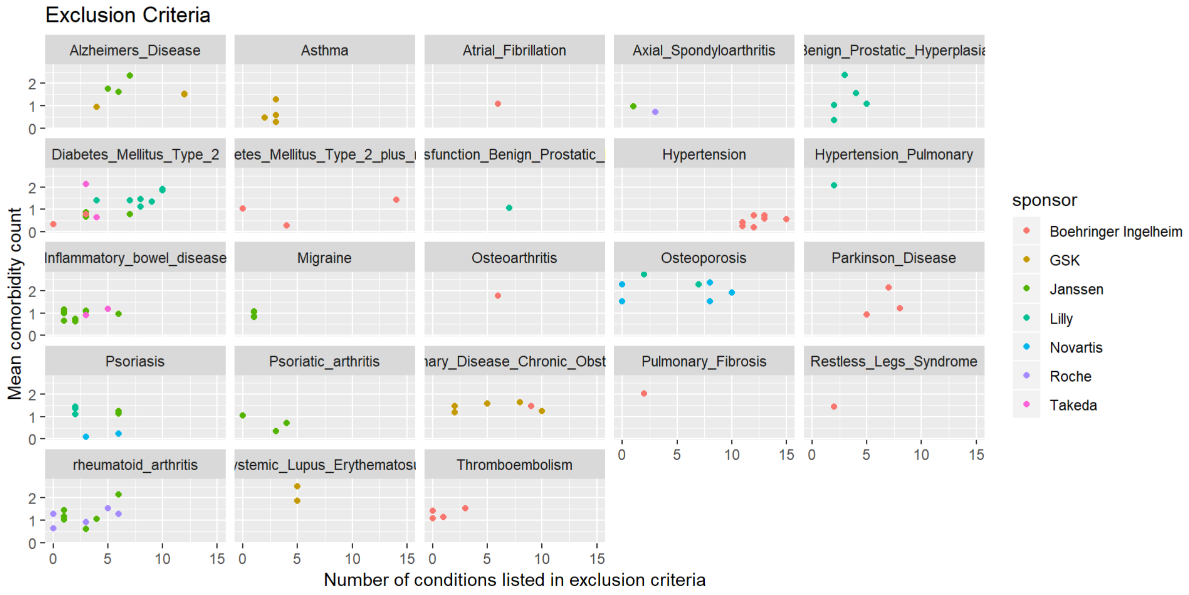

Figure S11.5: Exclusion Criteria and number of participants

Previous Next
